# Reevaluating *Emx* gene phylogeny: homopolymeric amino acid tracts as a potential factor obscuring orthology signals in cyclostome genes

**DOI:** 10.1186/s12862-015-0351-z

**Published:** 2015-05-04

**Authors:** Miyuki Noro, Fumiaki Sugahara, Shigehiro Kuraku

**Affiliations:** Genome Resource and Analysis Unit, RIKEN Center for Developmental Biology, 2-2-3 Minatojima-minami, Kobe, 650-0047 Japan; Division of Biology, Hyogo College of Medicine, 1-1 Mukogawa-cho, Nishinomiya, 663-8501 Japan; Evolutionary Morphology Laboratory, RIKEN, 2-2-3 Minatojima-minami, Kobe, 650-0047 Japan; Phyloinformatics Unit, RIKEN Center for Life Science Technologies, 2-2-3 Minatojima-minami, Kobe, 650-0047 Japan

**Keywords:** *Emx*, Gene duplication, Cyclostome, Lamprey, Hagfish, Conserved synteny, Homopolymeric amino acid (HPAA) tracts

## Abstract

**Background:**

Vertebrate *Emx* genes, retained as multiple copies, are expressed in a nested pattern in the early embryonic forebrain and required for its regionalization. This pattern seems to have originated in a vertebrate common ancestor; however, a previous analysis, reporting two lamprey *Emx* genes, claimed independent *Emx* gene duplications in both cyclostome (extant jawless fish) and gnathostome (jawed vertebrate) lineages after their divergence. This scenario is neither parsimonious nor consistent with the hypothesis that genome expansion occurred before the cyclostome-gnathostome split, which is supported by recent genome-wide analyses.

**Results:**

We isolated and sequenced cDNA of two hagfish *Emx* genes and performed intensive molecular phylogenetic analyses, including the hagfish and/or lamprey *Emx* genes. The lamprey genes tended to attract each other in inferred phylogenetic trees, an effect that tended to be relaxed on inclusion of the hagfish genes. The results of these analyses suggest that cyclostome *EmxB* is orthologous to gnathostome *Emx2*, which was also supported by conserved synteny. Homopolymeric amino acid (HPAA) tracts represent a remarkable feature of the lamprey Emx sequences, and a comparative genome-wide scan revealed that lamprey proteins exhibit a unique pattern of HPAA tract accumulation.

**Conclusions:**

Our analysis, including hagfish *Emx* genes, suggests that gene duplications gave rise to *Emx1*, *-2* and *-3* before the cyclostome-gnathostome split. We propose that independent HPAA tract accumulations in multiple ancient duplicates, as identified in lamprey *Emx* gene products, may have led to erroneous identification of gene duplication in the lamprey lineage. Overall, our reanalysis favors the scenario that the nested *Emx* expression pattern in mouse and lamprey shares a common origin.

**Electronic supplementary material:**

The online version of this article (doi:10.1186/s12862-015-0351-z) contains supplementary material, which is available to authorized users.

## Background

Homeobox-containing *Emx* genes of vertebrates play pivotal roles in the regionalization of the telencephalon [1-3]. In the early embryonic forebrain, *Emx2* is expressed widely in the pallium, which develops into the cerebral cortex, while the expression of *Emx1* is observed in the dorsal, medial and lateral pallium, but not the ventral pallium which later differentiates to form a part of the claustroamygdaloid complex in mammals [4]. This nested pattern has been reported commonly in jawed vertebrates, including the mouse [5,6], zebrafish [7,8], and small spotted catshark [9].

**Figure 1:**
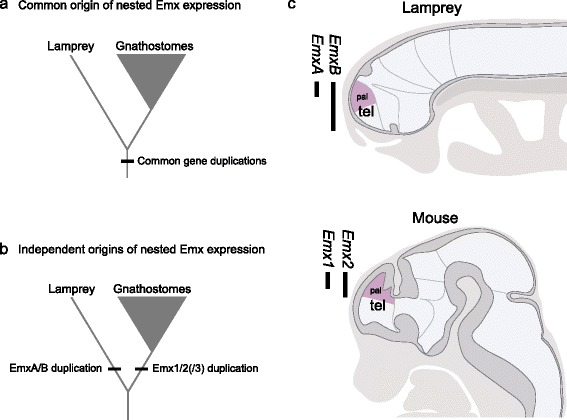
Alternative scenarios for timing of *Emx* gene duplication. **(a)** Scenario supporting the common Emx duplication before the cyclostome-gnathostome split. In this scenario, the establishment of the nested Emx expression could have occurred once before the cyclostome-gnathostome split just after the gene duplication. **(b)** Scenario supporting independent Emx duplications in both of the lamprey and gnathostome lineages. In this scenario, the nested *Emx* gene expression (see Background) is assumed to have been established independently in the two lineages, as a result of convergence. **(c)** Nested expression patterns of *Emx* genes in the embryonic brains of sea lamprey and mouse. The ranges of the gene expressions including the pallium (pal) in the telencephalon (tel) along the anteroposterior axis are indicated with the lengths of the thickened vertical bars.

Tank et al. reported two *Emx* genes of sea lamprey, a jawless fish, which are also expressed in a nested pattern, as in gnathostomes (jawed vertebrates) [10]. They performed molecular phylogenetic analyses including these two sea lamprey *Emx* genes, and suggested that they duplicated uniquely in the lineage leading to lampreys, independently from the gene duplication that gave rise to *Emx1* and *-2* of jawed vertebrates [10] (Figure 1b). This suggests that the nested expression patterns in cyclostome (extant jawless fish) and gnathostome lineages converged from independent origins; however, principle of parsimony suggests that these expression patterns in these diverse vertebrates is more likely to share a common ancestry (Figure 1a).

A previous study showed that the genome expansion that accompanied massive gene duplications (‘two-round whole genome duplications’ abbreviated in 2R-WGD) occurred before the cyclostome-gnathostome split [11] scenario finding that was also supported by later studies [12,13]. Nonetheless, assigning orthology of jawless fish genes to gnathostome counterparts is often not straightforward [14], possibly due to the high GC content and biased amino acid composition of lamprey genes [12,15,16].

In this study, we reexamined the molecular phylogeny of *Emx* genes and investigated lamprey-specific sequence characteristics affecting molecular phylogenetic analyses. We sought to address the question of whether the nested *Emx* gene expressions were established a single time in the vertebrate common ancestor or independently in both gnathostome and cyclostome lineages.

## Methods

### Isolation and sequencing of cDNA for cyclostome *Emx* genes

**Figure 2:**
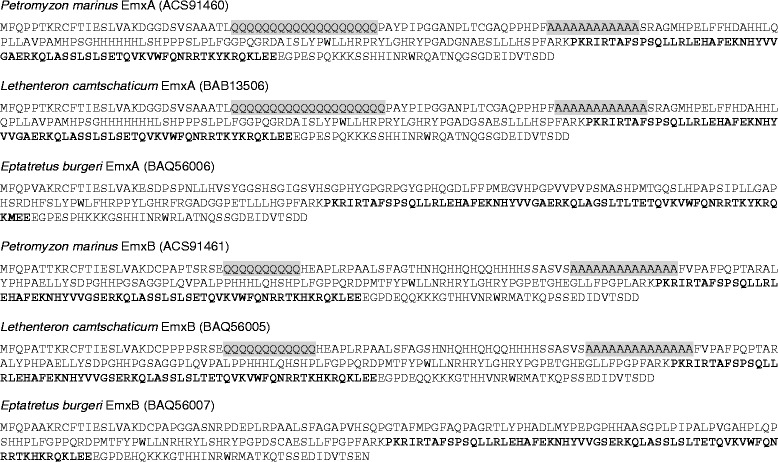
Deduced amino acid sequences of the cyclostome Emx genes. The putative full-length amino acid sequences deduced from the nucleotide cDNA sequences are shown for *EmxA* and *-B* of the sea lamprey (*P. marinus*), Japanese lamprey (*L. camtschaticum*) and inshore hagfish (*E. burgeri*). Homeodomains are indicated with bold letters. HPAA tracts of size 10 or larger are indicated with gray background.

Total RNAs were extracted from the adult liver of the inshore hagfish (*Eptatretus burgeri*) and embryos of the Japanese lamprey (*Lethenteron japonicum* or *L. camtschaticum*). These were used as templates for cDNA synthesis with the 3ˊRACE System (Invitrogen). For each of these two species, the prepared cDNA was used as templates for PCR with the forward degenerate primer 5ˊ-CGN GCN TTY GAR AAR AAY CAY TAY GT-3ˊ corresponding to the conserved amino acid stretch (H/R)AFEKNHYV and the AUAP primer supplied in the 3ˊRACE System (Life Technologies). The product of this amplification was used in the nested PCR with the forward degenerate primer 5ˊ-C GAR AAR AAY CAY TAY GTN GTN GG-3ˊ corresponding to the conserved amino acid stretch EKNHYVVG and the AUAP primer. cDNA cloning and sequencing were performed as described previously [17], to identify cDNA sequences of the inshore hagfish *EmxA* and *EmxB* as well as that of the Japanese lamprey *EmxB*. The upstream sequences of the cDNAs were amplified and sequenced using the GeneRacer kit (Life Technologies). These sequences were deposited in DDBJ under the accession numbers, AB935430-AB935432, with their deduced amino acid sequences (Figure 2). The experiment was conducted according to the institutional and national guideline for animal ethics.

### Sequence retrieval

Protein sequences showing significant similarity to the sea lamprey genes [accession IDs: *Emx*, ACS91460 and ACS91461; *Pdzd8* (PDZ domain containing 8); ENSPMAP00000007079; ENSPMAP00000006415] were retrieved from the Ensembl Genome Browser [18] using aLeaves [19] (Additional files 1 and 2). The *Emx* and *Pdzd8* sequences of the sea lamprey (*Petromyzon marinus*), the spotted gar (*Lepisosteus oculatus*) and the little skate (*Leucoraja erinacea*), which are not available as full-length sequences in Ensembl, were retrieved manually from their respective genome assemblies (version LepOcu1 available at Ensembl *Pre!* for spotted gar and version 1 available at SkateBase for little skate; [20]). The curated nucleotide sequences and their deduced amino acid sequences are included in (Additional files 3 and 4).

### Phylogenetic analysis

Protein sequences collected as described above were aligned using MAFFT version 7.215 [21] and trimmed manually based on the results of TrimAl v1.2 [22] with no gap allowed and a similarity threshold (st) of 0.0005. For every data set, the optimal model for amino acid substitutions was chosen according to the results of ProtTest 3 [23]. Heuristic maximum-likelihood (ML) tree inference was performed using PhyML v3.0 [24]. Exhaustive ML analysis with constraints of relationships inside particular taxa was performed using TreePuzzle 5.2 [25], by inputting all possible tree topologies in a ‘user defined tree’ mode. Statistical tests of tree topologies and probabilistic counts of gene duplications were performed using CONSEL version 0.20 [26], as previously reported [11]. Preliminary neighbor-joining (NJ) trees were inferred using ClustalW version 2.1 [27] at default settings.

Numbers of synonymous substitutions (*K*_s_) per site were computed with the program yn00 at the online tool Phylemon 2 (http://phylemon.bioinfo.cipf.es; [28]) in which PAML version 4.4c [29] is implemented, using nucleotide sequence alignment of the relevant gene pair for protein-coding regions.

### Synteny analysis

We compared genomic scaffolds containing gnathostome and cyclostome *Emx* genes using the Ensembl genome browser (release 74). For sea lamprey and Japanese lamprey, Emx-containing scaffolds were retrieved from Ensembl or from Japanese lamprey Genome Project (genome assembly LetJap1.0) ([30]; http://jlampreygenome.imcb.a-star.edu.sg/), and prediction of protein-coding genes by using Augustus with the species parameter set to ‘*Petromyzon marinus*’ ([31]; http://augustus.gobics.de/).

### Evaluation of multiple sequence alignment methods

The two lamprey *Emx* genes and 24 other *Emx* genes used in the ML analysis describe above were employed in this *in silico* deletion analysis. Their full-length sequences before and after the deletion of the Q-tracts and A-tracts in the two lamprey Emx peptide sequences were aligned using MAFFT version 7.215 [21], T-COFFEE version 9.03 [32], and ClustalW 2.1 [33] with default settings. The resultant multiple alignments were trimmed with TrimAl v1.2 [22] with five different settings: (1) no gap allowed, (2) no gap allowed and the similarity threshold ‘st’ of 0.00005, (3) no gap allowed and st = 0.0001, (4) no gap allowed and st = 0.0005, and (5) no gap allowed and st = 0.001. Using the selected amino acid sites, the datasets were subjected to substitution model selection with ProtTest 3 [23]. As JTT + Γ4 was chosen as the optimal model for most of the datasets, we uniformly applied this model to compare the results between the different datasets. Maximum-likelihood analysis was performed with TreePuzzle 5.2 [25] by inputting all possible tree topologies consisting of six OTUs (gnathostome *Emx1-3*, lamprey *EmxA*, lamprey *EmxB* and outgroup) in ‘user defined tree’ mode. We computed approximate bootstrap probabilities (BP) for individual tree topologies with resampling of estimated log-likelihoods (RELL) [34] with CONSEL [26]. To evaluate variable alignment results, the degrees of support for the exclusive grouping of lamprey *EmxA* and *EmxB* were quantified as the sum of RELL BP for the tree topologies supporting this relationship.

### Detection of homopolymeric amino acid (HPAA) tracts

We analyzed the protein datasets for three representative vertebrates (i.e., human, zebrafish, sea lamprey). In order to ensure the collection of protein-coding sequences with evidence of transcription, NCBI RefSeq Proteins ([35]; http://www.ncbi.nlm.nih.gov/refseq/) were used for the analyses of human and zebrafish datasets (downloaded on July 16, 2013). For the sea lamprey, a sequence dataset without splicing variants (n = 24,271), provided by the Genome Consortium ([12]; http://genome.wustl.edu/genomes/detail/petromyzon-marinus/), was adopted. To validate the results based on this Genome Consortium dataset, for the sea lamprey, we used ‘all and known proteins’ sequences (n = 11,442) available at Ensembl release 72 (ftp://ftp.ensembl.org/pub/release-72/fasta/petromyzon_marinus/pep/Petromyzon_marinus.Pmarinus_7.0.72.pep.all.fa.gz). mRNA-derived protein sequences (n = 1,088) available at NCBI Proteins (http://www.ncbi.nlm.nih.gov/protein; downloaded on July 16, 2013), which presumably do not exhibit false-positive identification of HPAA tracts in non-coding sequences (e.g., introns), were also collected and analyzed. For these datasets, we selected one representative peptide sequence with the largest length among multiple alternative splicing variants for each gene.

To exclude species-specific sequences that might represent false-positive gene prediction, for each of the three species above, we prepared a set of sequences for which homologs are present in the other two species. The threshold for this cross-species matching was a bit score of no less than 200 in reciprocal BLASTP searches [36] with the options ‘-seg yes -soft masking true’.

In the datasets for these three species prepared as above, we identified and counted homopolymeric amino acid (HPAA) tracts with stretches occupied by identical amino acids for no less than six consecutive residues. To validate the results with this criterion, we performed the same count under other criteria with eight and ten consecutive residues. Chi-square test and two-tailed test of population proportion with R 3.1.2 (http://www.r-project.org) were employed in the statistical evaluation of significance of differences in the proportions of HPAA tract-containing peptides and in the proportions of amino acids contained in HPAA tracts between species. Likewise, the frequencies of HPAA tracts consisting of no less than 12 amino acids among those consisting of no less than six amino acids were compared between species and assessed with two-tailed test of population proportion.

### Gene Ontology analysis

Overrepresentation of Gene Ontology (GO) terms was analyzed using FatiGO ([37]; http://babelomics.bioinfo.cipf.es/). After obtaining the set of HPAA tract-containing sequences for each species, we performed BLASTP searches and identified the most similar human Ensembl peptide for each of the sequences. Human Ensembl gene IDs associated with the identified Ensembl peptides were submitted for overrepresentation analysis using the default settings in the FatiGO web server.

## Results

### Emx gene repertoire in diverse vertebrates including cyclostomes

To make a comprehensive comparison of vertebrate *Emx* genes, we collected a wide range of vertebrate Emx sequences, including cyclostomes. We newly determined the nucleotide sequences of hagfish *EmxA*, *EmxB*, and the Japanese lamprey *EmxB* (*LjEmxB*). Previously, two *Emx* sequences were identified in *Petromyzon marinus* (*PmEmxA* and *PmEmxB*), while only one was reported for *Lethenteron japonicum* (*LjEmx*). We computed the number of synonymous substitutions (*K*_s_) per site for the two pairs of sequences (*LjEmx*-*PmEmxA* and *LjEmxB*-*PmEmxB*) between these species. The *EmxA* pair resulted in the *K*_s_ of 0.089, and the *EmxB* pair 0.113. According to the previous report of the standard *K*_s_ value for this species pair (0.15 ± 0.09; [15]), we confirmed the orthologies of *EmxA* and *EmxB* in these two species, and here designate the previously reported *LjEmx* as *LjEmxA*.

As reported above, two *Emx* homologs were identified in all the three cyclostome species analyzed in this study (Figure 2; Additional file 1). Orthology between hagfish and lamprey was assessed with molecular phylogenetic analysis based on exhaustive maximum-likelihood (ML) method (Additional file 5). In particular, the orthology of hagfish *EmxB* to lamprey *EmxB* was supported in all tree topologies showing a smaller **Δ**log*L* than its standard error (Additional file 5). The orthology between hagfish *EmxA* and lamprey *EmxA* was also supported in most of those tree topologies (Additional file 5).

Intensive analyses show that the *Emx3* ortholog has been retained in the genomes of marsupials, amphibians, bony fishes (e.g., coelacanth) and cartilaginous fishes (e.g., little skate), but not in eutherian mammals, birds or reptiles (Additional file 1), consistent with previous reports ([2,7]; reviewed in [38]).

### Phylogeny of cyclostome Emx genes

**Figure 3:**
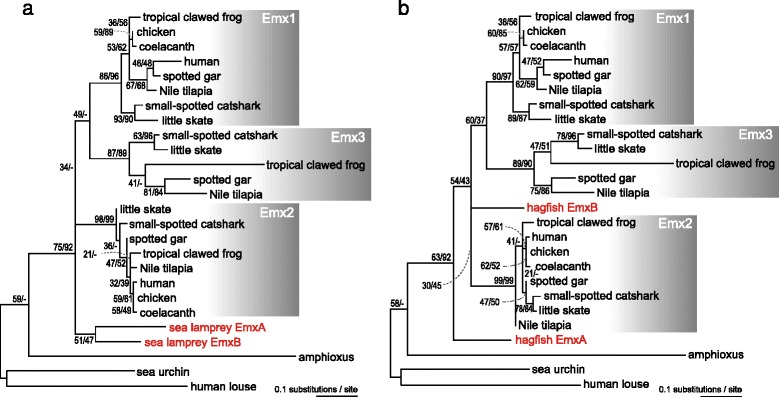
Maximum-likelihood tree for vertebrate *Emx* genes with either cyclostome species. These ML trees were reconstructed using 132 amino acid sites in the multiple alignment in Additional file 7 under the JTT model [49] with among-site rate heterogeneity taken into account by assuming the gamma distribution [50] with four rate categories (JTT + Γ_4_ model). To evaluate the effect of the choice of cyclostome sequences, we performed the analysis including sea lamprey **(a)** or hagfish **(b)**, with a shape parameter of the gamma distribution α of 0.37 and 0.38, respectively. The numbers at nodes indicate bootstrap probabilities with 100 replicates for the ML and NJ methods, in order.

In order to test alternative hypotheses concerning the origin of the nested expression pattern of *Emx* genes (Figure 1), molecular phylogeny of vertebrate *Emx* genes was examined with a more operational strategy. We first performed heuristic ML analysis including both hagfish and lamprey without any *a priori* constraint of phylogenetic relationship. This analysis did not result in exclusive clustering of cyclostome *EmxA* and *-B* (Additional file 6). To focus on relationships between cyclostome genes and the three gnathostome *Emx* genes (*Emx1*, *-2* and *-3*), we constrained relationships among gnathostome species within the three subtypes. The dataset employed in this analysis corresponded to six operational taxonomic units (OTUs), and all possible tree topologies with six OTUs (105 tree topologies) showed *P* values of no less than 0.05 in KH and SH tests (Table 1). When hagfish *Emx* genes were excluded, two lamprey *Emx* genes clustered together, but with low support values (bootstrap probabilities in the NJ and ML methods, 47 and 51; Figure 3a; see Additional file 7 for the sequence alignment used). Importantly, when lamprey genes were excluded, hagfish *EmxB* clustered with gnathostome *Emx2* (Figure 3b, bootstrap probabilities in the NJ and ML methods, 45 and 30). When only one of the cyclostome genes was included, gnathostome *Emx2* clustered with lamprey *EmxB*, as well as with hagfish *EmxB* (Additional file 8), although bootstrap probabilities were not high in either case. Our phylogenetic trees supported a proximity of human to actinopterygian fishes in the *Emx1* subgroup (Figure 3; Additional files 6 and 8), and indicate its more complicated evolutionary history within this group. However, synteny conservation around Emx1 genes corroborates the orthology between them [2], and the relationship in our trees, which is inconsistent with generally accepted species phylogeny, may be caused by long branch attraction driven by rapid evolution of mammalian Emx1 sequences.

**Table 1:** **ML analysis of vertebrate** ***Emx*** **gene phylogeny**

**Rank**	**Tree topology**	**log** ***L***	**Δlog** ***L*** **± SE**	***P*** **value**	
				**1sKH**	**SH**
1	(((((g1,g3),g2),cB),cA),outgroup)	-1909.94	ML	1.00	1.00
2	(((((g1,g3),cB),g2),cA),outgroup)	-1910.93	0.99 ± 1.84	0.27	0.82
3	((((g1,g3),(g2,cB)),cA),outgroup)	-1910.93	0.99 ± 1.84	0.26	0.82
4	(((((g1,g3),g2),cA),cB),outgroup)	-1911.62	1.68 ± 2.27	0.22	0.74
5	((((g1,g3),g2),(cA,cB)),outgroup)	-1911.62	1.68 ± 2.27	0.20	0.74
6	((((g1,g3),(cA,cB)),g2),outgroup)	-1912.39	2.45 ± 2.56	0.17	0.63
7	(((g1,g3),((cA,cB),g2)),outgroup)	-1912.39	2.46 ± 2.55	0.17	0.63
8	((((g1,g3),(g2,cB)),cA),outgroup)	-1912.61	2.68 ± 2.91	0.17	0.60
9	((((g1,g3),cB),(g2,cA)),outgroup)	-1912.61	2.68 ± 2.91	0.16	0.60
10	(((((g1,g3),cA),g2),cB),outgroup)	-1912.61	2.68 ± 2.91	0.17	0.60
11	((((g1,g3),cA),(g2,cB)),outgroup)	-1912.61	2.68 ± 2.91	0.17	0.60
12	(((((g1,g3),cB),cA),g2),outgroup)	-1912.61	2.68 ± 2.91	0.19	0.60
13	(((g1,g3),((g2,cB),cA)),outgroup)	-1912.61	2.68 ± 2.91	0.18	0.60
14	(((g1,g3),((g2,cA),cB)),outgroup)	-1912.61	2.68 ± 2.91	0.18	0.60
15	(((((g1,g3),cA),cB),g2),outgroup)	-1912.61	2.68 ± 2.91	0.19	0.60

*Abbreviation:* *SE* standard error, *1sKH* One-sided Kishino-Hasegawa test, *SH* Shimodaira-Hasegawa test, *g1* gnathostome *Emx1*, *g2* gnathostome *Emx2*, *g3* gnathostome *Emx3*, *cA* cyclostome *EmxA*, *cB* cyclostome *EmxB.*

### Cyclostome-gnathostome orthology assessed with conserved synteny

**Figure 4:**
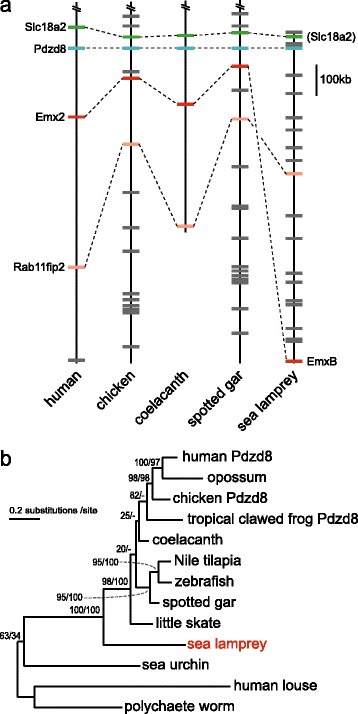
Evidence for orthology between cyclostome EmxB and gnathostome Emx2. **(a)** Synteny conservation between the genomic regions containing *Emx2* of gnathostomes and *EmxB* of sea lamprey. Assignment of gene names in this figure was based on orthology information in Ensembl except the one in a parenthesis (“Slc18a2”) which was assigned by using BLASTP. **(b)** Molecular phylogeny of vertebrates *Pdzd8* genes, included in the *Emx*-containing genomic regions in (a). This ML tree was inferred using 600 amino acid sites in the multiple alignment in Additional file 10, assuming the JTT model with amino acid frequencies taken into account (JTT + Γ_4_ + F model) (shape parameter α = 1.17). The numbers at nodes indicate bootstrap probabilities with 100 replicates for the ML and NJ methods, in order.

To confirm the orthology between cyclostome *EmxB* and gnathostome *Emx2* suggested above, we compared the synteny in genomic regions containing these genes. The sea lamprey scaffold containing *EmxB* (scaffold GL476962 in Ensembl), which is approximately 1.8 Mbp long, contains several genes homologous to those in the *Emx2*-containing regions in the gnathostome genomes (Figure 4a; Additional file 9). Among the neighboring genes is *Pdzd8*, identified as a single copy in all species analyzed. Molecular phylogeny of this gene is consistent with generally accepted species phylogeny (Figure 4b; see Additional file 10 for the sequence alignment used), suggesting one-to-one orthology between the lamprey and jawed vertebrate *Pdzd8* genes. This probable orthology, as a tier of genomic regions containing those genes, indicates orthology between the *EmxB*-containing region in the lamprey genome and *Emx2*-containing region in jawed vertebrates. This one-to-one orthology between the *EmxB*-containing genomic region in sea lamprey and the *Emx2*-containing genomic region, supported by the *Pdzd8* phylogeny, indicates orthology between the lamprey *EmxB* and gnathostome *Emx2*.

The sea lamprey genomic scaffold containing *EmxA* (Ensembl GL481279), which is less than 24 Kbp, does not contain any other genes, providing no clue for its orthology to jawed vertebrate homologs. In the genome of the Japanese lamprey, *EmxA* is located in a 5.1 Mbp-long scaffold (scaffold00019 in the assembly version LetJap1 available at http://jlampreygenome.imcb.a-star.edu.sg) and is surrounded by potential orthologs of the genes flanking the *Emx1* gene (*Rab11fip5*, *Dysferlin*, *Cyp26b1*, and *Exoc6b)* in gnathostome genomes. Although all of them have paralogs derived from 2R-WGD, this result suggests possible orthology of the cyclostome *EmxA*-containing genomic region to the gnathostome *Emx1*-containing genomic region.

### Timing of Emx gene duplications

While our synteny analysis supports common origin for multiple *Emx* genes (Figure 1a), the ML trees in our molecular phylogenetic analysis did not yield unequivocal results (Figure 3), especially because multiple tree topologies were supported with similar log-likelihood values (Table 1). In order to scrutinize overall trend in the result of our ML analysis, we performed probabilistic counts of gene duplication [11]. From an entire ML tree inference, considering all possible tree topologies, this analysis yields the number of gene duplication before the cyclostome-gnathostome split (*N*_*bef*_) as well as the number of gene duplication after the cyclostome-gnathostome split (*N*_*aft*_). We employed the data set used in Figure 3 for gnathostomes and outgroups, with variable sets of cyclostome sequences. As a result, *N*_*bef*_ was shown to be larger than *N*_*aft*_ in all the cases we analyzed (Table 2), suggesting the *Emx* gene duplication before the cyclostome-gnathostome split (Figure 1a). The number of gene duplications in the cyclostome lineage (*N*_*cyc*_) was shown to be markedly smaller than *N*_*bef*_ and *N*_*aft*_, (< 0.1) when hagfish sequences were included in the data set (Table 2).

**Table 2:** **Probabilistic count of** ***Emx*** **gene duplications based on the ML method**

**Cyclostome included**	**ML tree topology**	***N***_**bef**_	***N***_**aft**_	***N***_**cyc**_	***N***_**unk**_
Sea lamprey and hagfish	(((((g3,g1),g2),cB),cA),outgroup);	1.36	1.32	0.09	0.18
Sea lamprey	((((g3,g1),g2),(cB,cA)),outgroup);	1.21	0.93	0.63	0.21
Hagfish	((((cB,g2),(g3,g1)),cA),outgroup);	2.02	0.56	0.02	0.34

*Abbreviation:* *g1* gnathostome *Emx1*, *g2* gnathostome *Emx2*, *g3* gnathostome *Emx3*, *cA* cyclostome *EmxA*, *cB* cyclostome *EmxB*. See Kuraku et al. [11] for details of *N*_bef_, *N*_aft_, *N*_cyc_, and *N*_unk_.

According to the results of ProtTest3, JTT+I+Γ_4_ model was employed for the analysis with both sea lamprey and hagfish, while JTT+Γ_4_ model was employed for the analyses with either of sea lamprey and hagfish.

### Homopolymeric amino acid (HPAA) tracts: effect of lamprey-specific sequence characteristics

**Figure 5:**
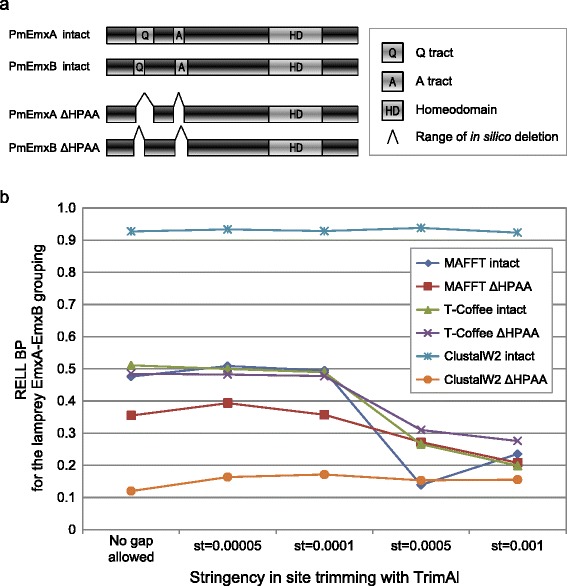
Examination on the effect of HPAA tracts in lamprey Emx sequences. **(a)** Schematics of lamprey Emx sequences used in this analysis. The A-tract (A) and Q-tract (Q) correspond to those indicated with gray background in Figure 2. The two intact or artificially modified (ΔHPAA) lamprey Emx sequences were aligned with their homologs by using ClustalW, MAFFT, or T-Coffee. The resultant multiple alignments were passed on to selection of amino acid sites using TrimAl with different settings (see Methods). **(b)** Comparison of degrees of the support for the lamprey EmxA-EmxB grouping. RELL bootstrap probabilities for this relationship were computed in a ML analysis by inputting datasets prepared with the variable stringencies in site trimming with TrimAl (no gap allowed and st of 0.00001 to 0.001), as well as variable choices of alignment programs and the presence or absence of the HPAA tracts (see Methods).

Our synteny analysis, based solely on gene locations, suggested one-to-one orthology between cyclostome *EmxB* and gnathostome *Emx2* (Figure 4). However, when hagfish genes were excluded, the molecular phylogenetic tree did not support such one-to-one orthology, and suggested instead a lamprey lineage-specific gene duplication between *EmxA* and *–B*, (Figure 3a). To investigate the cause of this inconsistency thoroughly, we analyzed the multiple alignment of amino acid sequences, and identified long tracts of alanine (A) and glutamine (Q) in the N-terminal domain of both sea lamprey EmxA and -B (Figure 2; Additional file 7). Since hagfish *Emx* genes have neither of the homopolymeric amino acid (HPAA) tracts (Additional file 7), the two lamprey *Emx* sequences are thought to have acquired these tracts independently after the separation of this lineage from the ancestral lineage leading to hagfishes. To examine possible effect of the HPAA tracts on phylogenetic analyses, we artificially deleted them (Figure 5a) and analyzed its impact on multiple sequence alignment with different alignment programs. Remarkably, when intact sequences with HPAA tracts were employed, alignment with ClustalW solely resulted in markedly high support for the exclusive clustering of lamprey *EmxA* and *–B* (Figure 5b). This effect was weakened when HPAA tracts were deleted before alignment (Figure 5b). Taken together, our analysis reproduces the result by Tank et al. [10] and indicates that the presence of HPAA tracts may be the source of the erroneous conclusion.

**Figure 6:**
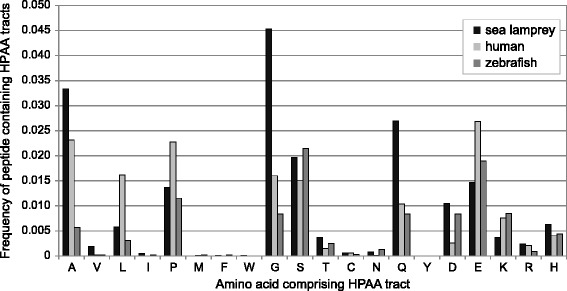
Genome-wide frequencies of homopolymeric amino acid tracts in the sea lamprey, human, and zebrafish. Peptide sequences with homopolymeric amino acid (HPAA) tracts (those with a stretch of no less than six consecutive residues) were extracted from public database and compared among the three vertebrates (see Methods for details). This figure shows the frequencies of HPAA tract-containing peptides. The results with the different criteria (stretches of no less than eight and ten consecutive residues) are shown in Additional file 12.

In order to identify any marked pattern of HPAA accumulation unique to lampreys, we compared the frequencies of HPAA tract-containing peptides for each amino acid among human, zebrafish, and sea lamprey. HPAA tracts were observed widely throughout the genomes of these three species (Additional file 11), as reported previously [39]. Between the three species, there were significant differences in the frequency of HPAA tract-containing peptides (Consortium dataset: p < 0.0001; NCBI mRNA dataset: p < 0.01). Moreover, the frequencies of peptides containing HPAA tracts for individual amino acids exhibited marked difference in these species (Figure 6; Additional file 12). In sea lamprey, peptides with tracts of Q and glycine (G) were observed nearly three-fold more frequently than in human and zebrafish (p < 0.0001). This tendency was also observed with the Ensembl peptide dataset (G: lamprey, 2.8%; human, 1.6%; zebrafish, 0.9%; p < 0.0001; Q: lamprey, 1.9%; human, 1.0%; zebrafish, 0.8%; p < 0.0001 for lamprey-zebrafish and lamprey-human differences) and mRNA-derived peptide sequences in NCBI Protein (G: lamprey, 8.2%; human, 2.9%; zebrafish, 1.4%; p < 0.05; Q: lamprey, 7.0%; human, 1.5%; zebrafish, 0.4%; p < 0.05).

We also compared the composition of HPAA tracts between the three species, taking the number of HPAAs in the tracts into consideration. The proportion of amino acids contained in HPAA tracts was significantly higher in sea lamprey (0.36%; p < 2.2e-16) than in human (0.18%) and zebrafish (0.13%) (Additional file 11). The proportion of A (alanine), as well as G and Q, contained in HPAA tracts were significantly higher in sea lamprey than in human and zebrafish (p < 0.00005). Also, we analyzed the frequency of the HPAA tracts consisting of no less than twelve amino acids in HPAA tracts consisting of no less than six amino acids. Overall, it was slightly higher in human (8.6%) than in sea lamprey (7.0%) and zebrafish (5.1%) but, the difference was not significant. Only the frequency of poly-Q tracts consisting of no less than twelve amino acids was significantly higher in human (23.1%; p < 0.05) than in sea lamprey (9.6%) and zebrafish (10.6%).

We analyzed functional properties of HPAA tract-containing peptides using Gene Ontology (GO) terms for molecular function. Additional file 13 shows GO terms overrepresented commonly in Genome Consortium dataset and Ensembl dataset (see Methods for the detail of these datasets). GO terms commonly overrepresented in both datasets were identified for peptides containing homopolymeric tracts of proline (P), Q, and G, but no GO term for molecular function was overrepresented for alanine (A) tract-containing peptides of the sea lamprey. Among the overrepresented terms, those associated with transcriptional regulation were dominant, regardless of species (Additional file 13). Lamprey-specific overrepresentations were detected for terms “amine binding” and “neurotransmitter binding” for Q tract-containing peptides, and “enzyme activator activity” for G tract-containing peptides (Additional file 13).

## Discussion

We reanalyzed the *Emx* gene phylogeny with sequence information from diverse species (hagfish, little skate, spotted gar and coelacanth) from statistical and genomic viewpoints. Our tree inference did not reproduce the extremely high support for the sea lamprey *EmxA*-*EmxB* clustering reported in a previous study (NJ bootstrap value, 99.4; ML quartet puzzling support value, 97; [10]). In our analysis, support values for the sea lamprey *EmxA*-*EmxB* clustering were only around 50, at the largest (Figure 3a). Moreover, the support for gene duplication in the cyclostome lineage decreased when hagfish sequences were included in the input data set (Additional file 6). This trend was also observed in a probabilistic count of gene duplications (Table 2). Overall, our phylogenetic analysis favored the *Emx* gene duplication before the cyclostome-gnathostome split (Figure 1a).

Notably, our scan of the multiple sequence alignment of *Emx* gene products detected homopolymeric amino acid (HPAA) tracts (also known as homopeptides or amino acid tandem repeats) co-occurring between the peptide sequences of sea lamprey *EmxA* and *-B*. In our analysis, ClustalW, adopted by the previous study by Tank et al. [10], aligned tightly the HPAA tracts in those sequences, whereas MAFFT and T-Coffee largely relaxed this effect (Additional file 14). The alignment produced by ClustalW, from which non-conserved sites including the HPAAs were deleted, lent strong support of an exclusive clustering of the two lamprey Emx genes (Figure 5b). When the HPAA tracts were excluded prior to alignment (Figure 5a), the support for the lamprey *EmxA*-*EmxB* grouping values became as low as that from the datasets prepared with the other alignment programs (Figure 5b). Interestingly, the HPAAs influenced to the alignment in neighboring regions and then to phylogenetic tree inference, only with ClustalW as far as we examined (Figure 5b). Thus, we concluded that the co-occurrence of those HPAA tracts in these two duplicates led to erroneously strong support for the *EmxA*-*EmxB* clustering in the previous study [10].

As no information was available for lampreys, we have performed genome-wide quantification of HPAA tracts. Among the three species examined, significant difference was observed in amino acid composition of HPAA tracts, as well as the frequency of HPAA tract-containing peptides (Figure 6). First, our analysis detected the high proportion of poly-Q tracts consisting of no less than twelve amino acids in human. This tendency is probably common among mammals including rodents, according to a previous study (see Figure 1 in [40]) and could have been driven by selection pressure unique to the poly-Q accumulation. Second, when HPAA tracts consisting of no less than six amino acids were quantified, sea lamprey exhibited a unique pattern of HPAA accumulation, with markedly high frequency of peptides containing HPAA tracts consisting of particular amino acids and their compositions with the number of contained homopolymeric amino acids taken into account (Figure 6; Additional files 11 and 12). In this analysis, we paid close attention to false-positive identification of HPAA caused by false prediction of protein-coding regions in the sea lamprey genome, and verified the result with three sequence data sets differently derived. HPAA accumulation should be recognized as a factor misleading lamprey gene phylogeny, together with other factors previously identified, such as high GC-content, codon usage bias and amino acid composition [12,16].

Consistent with the result of our molecular phylogenetic analysis, our synteny analysis supported the one-to-one orthology between cyclostome *EmxB* and gnathostome *Emx2* (Figure 4a). In general, synteny data involving cyclostome genes should be carefully interpreted because not only a certain gene of interest but also its flanking genes should exhibit ambiguous signals to multiple potential orthologs of gnathostomes (reviewed in [14]). In the present study, we identified the *Pdzd8* gene in the vicinity of the *Emx2* gene; *Pdzd8* is present as a single copy (without any paralog that arose in the vertebrate lineage) in all chordate genomes analyzed to date (Figure 4b). For this reason, we assumed that proximity to the *Pdzd8* gene could be regarded as a unique signal of orthology to *Emx2*. In the sea lamprey genome, *EmxB* and the *Pdzd8* ortholog were identified within 0.9 Mbp on the same scaffold (Figure 4a), suggesting orthology between *Emx2* and *EmxB*. We cannot completely rule out the possibility that the *Pdzd8* gene experienced an ancient duplication, and that subsequent differential gene loss resulted in paralogy between the gnathostome *Pdzd8* and the sea lamprey *Pdzd8*-like gene in the vicinity of *EmxB*. However, in addition to the possible *EmxB*-*Emx2* orthology, the *EmxA*-*Emx1* orhology was also suggested by our analysis (see Results). In conclusion, our study favored the common *Emx* gene duplication and one-to-one orthology of cyclostome *EmxA* and *-B* to gnathostome *Emx1* and *-2*, respectively.

We identified two or three *Emx* homologs in all jawed vertebrates. Although the third homolog has been lost in reptiles, birds, and eutherian mammals, other taxa, including marsupials, have retained *Emx3*. While mouse *Emx2* plays a major role in development of dorsal telencephalon [3], *Emx3* has been shown to be the most widely expressed and indispensable *Emx* homolog in zebrafish [2]. Its orthologs in opossum and *Xenopus*, which seem to be under less functional constraint as indicated by unique indels in their peptide sequences, remain poorly characterized. It would be intriguing to examine how Emx2 and Emx3 function in marsupials, as the *Emx1* ortholog was reported to be pseudogenized at least in opossum [2].

The expression domains of *EmxB* and *Emx2* encompass those of *EmxA* and *Emx1*, respectively [1,3,10]. Our results suggest that the similar expression pattern between cyclostome and gnathostome *Emx* is the consequence of shared ancestry, not convergence. We conclude that the nested expression pattern observed for the duplicated *Emx* genes was acquired in the common ancestor of all extant vertebrates (Figure 1a). Dorso-ventral subdivision of the pallium by the restricted expression of *Emx1/A* may thus have been acquired before the divergence between cyclostomes and gnathostomes, as proposed previously [41].

Some differences in expression domains of developmental regulatory genes, including *Emx*, are observed in developing brains in lamprey and gnathostomes. For instance, the expression of lamprey *EmxB* at early embryonic stages is expanded more ventrally than that of mouse *Emx2* [10], and the onset of FGF signaling in lamprey forebrain occurs at a later stage than in mice [42-44]. Analyses on knock-out mice show that FGF signaling is essential for proper induction of telencephalon and dorsal restriction of *Emx2* expression [45-48]. We thus suggest that ventral expansion of the early expression of lamprey *EmxB* may correspond to the later onset of FGF signaling in this animal. This hypothesis needs to be verified with further analyses, ideally involving hagfish, which represents the indispensable but currently missing link for reconstruction of the brain patterning program in the vertebrate ancestor.

## Conclusions

Our analysis, including hagfish *Emx* genes, suggests the occurrence of the gene duplications giving rise to *Emx1*, *-2* and *-3* before the cyclostome-gnathostome split. We propose that independent HPAA tract accumulations in multiple ancient duplicates may have led to erroneous identification of gene duplication in the lamprey lineage. Overall, our reanalysis concluded that the nested *Emx* expression pattern in mouse and lamprey shares the common origin before the split between the cyclostome and gnathostome lineages. A practical lesson from this study is that potential effect of unique properties of molecular sequences in lamprey can largely be relaxed thorough taxon sampling in other cyclostomes, such as hagfishes.

## Additional files

Additional file 1: Table S1.
*Emx* sequences used in this study. This table includes accession details of the sequences employed in phylogenetic tree inference for Figure 3 and Additional file 6: Figure S1. Additional file 2: Table S4.
*Pdzd8* sequences used in this study. This table includes accession details of the sequences employed in phylogenetic tree inference for Figure 4b. Additional file 3:
**Data S1.** Manually curated Emx sequences of spotted gar, coelacanth and little skate. Sources of the sequences before curation are shown in Additional file 1: Table S1. Additional file 4:
**Data S3. **Manually curated *Pdzd8* sequences of coelacanth, little skate and sea lamprey. Sources of the sequences before curation are shown in Additional file 2: Table S4. Additional file 5: Table S2. ML analysis for validation of hagfish-lamprey orthology of *EmxA* and *EmxB*. This analysis was performed with exhaustive ML method as described in Methods. Additional file 6: Figure S1. ML trees for vertebrate *Emx* genes including both sea lamprey and hagfish. (a) ML tree with the enriched dataset with more vertebrate sequences. This ML tree was inferred using 130 amino acid sites in the multiple alignment in Additional file 7: Data S2, assuming the JTT model with the proportion of invariable sites taken into account (JTT+Γ_4_+I model) (shape parameter α = 0.65). (b) ML tree inferred with selected sequences. This ML tree was inferred using 132 amino acid sites in the multiple alignment in Additional file 7: Data S2, assuming JTT +Γ_4_+I model (shape parameter α = 0.58). The numbers at nodes indicate bootstrap probabilities with 100. Additional file 7:
**Data S2.** Multiple sequence alignment of deduced amino acid sequences of Emx genes. The symbol ‘*’ indicates amino acid sites employed in the inference of the phylogenetic trees shown in Figure 3, Additional files 5: Table S2 and 6: Figure S1. The symbol ‘:’ indicates amino acid sites used in the inference of the phylogenetic trees shown in Figure 3, Additional 6: Figure S1b in Additional file 6: Figure S1, and Additional file 6: Figure S1 but not in that in Additional file 6: Figure S1a in Additional file 6: Figure S1. Accession numbers of the sequences included in this alignment are in Additional file 1: Table S1. Additional file 8: Figure S2. ML trees for vertebrate *Emx* genes with only one cyclostome *EmxB* gene. These ML trees were inferred using 132 amino acid sites in the multiple alignment in Additional file 7: Data S2, assuming JTT+Γ_4_ model (shape parameter α = 0.65). (a) The ML tree including sea lamprey *EmxB* as well as the jawed vertebrate data set. (b) The ML tree including hagfish *EmxB* as well as the jawed vertebrate data set. The numbers at nodes indicate bootstrap probabilities with 100 replicates for the ML and NJ methods, in order. Additional file 9: Table S3. Location of genes in the *Emx2*/*EmxB*-containing synteny. This table includes chromosomal locations (or locations on genomic scaffolds) and base positions of the genes in Figure 4a. Additional file 10:
**Data S4.** Multiple sequence alignment of deduced amino acid sequences of *Pdzd8* genes. The symbol ‘*’ indicates amino acid sites employed in the inference of the phylogenetic trees shown in Figure 4b. Accession numbers of the sequences included in this alignment are in Additional file 10: Data S4. Additional file 11: Table S5. Sequence dataset for counts of homopolymeric amino acid tracts. This table summarizes the details of the data sets prepared for sea lamprey, zebrafish and human. Additional file 12: Figure S3. Genome-wide frequencies of homopolymeric amino acid (HPAA) tracts with variable lengths in the sea lamprey, human, and zebrafish. The peptides with homopolymeric amino acid tracts (those with a stretch of no less than six, eight and ten consecutive residues) were extracted from public database and compared among the three vertebrates (see Methods for details). In the sea lamprey genome, Q and G tract-containing peptides were shown to be more than twice as frequent as in human and zebrafish genomes for all dataset we examined. Additional file 13: Table S6. Gene Ontology terms overrepresented among sea lamprey peptides with homopolymeric amino acid tracts. Gene Ontology terms revealed to be overrepresented both in Genome Consortium dataset and Ensembl dataset are listed. Additional file 14:
**Data S5.** Multiple sequence alignment of deduced amino acid sequences of Emx genes. Multiple sequence alignments produced by the three alignment programs ClustalW (a), MAFFT (b), or T-Coffee (c) are shown for a region containing the A-tract and the Q-tract in the lamprey Emx sequences that are indicated with gray background.

## Abbreviations

A: Alanine
Q: Glutamine
G: Glycine
P: Proline
OTU: Operational taxonomic unit
KH test: Kishino-Hasegawa test
SH: Shimodaira-Hasegawa test
NJ: Neighbor-joining
ML: maximum-likelihood
HPAA: Homopolymeric amino acid tract
