# Supplementary material for: Reevaluating Emx gene phylogeny: homopolymeric amino acid tracts as a potential factor obscuring orthology signals in cyclostome genes
Source: BMC Evol Biol. 2015 May 4;15:78. doi: 10.1186/s12862-015-0351-z (PMC4464114; doi:10.1186/s12862-015-0351-z)
Supplement: Additional file 1: Table S1. — Emx sequences used in this study. This table includes accession details of the sequences employed in phylogenetic tree inference for Figure 3 and Additional file 6: Figure S1. [file 12862_2015_351_MOESM1_ESM.pdf]

Additional file 1 (Table S1). Emx sequences used in this study

Taxon	Species	Gene name	Database	Accession ID	Notes
Cyclostomata	*Petromyzon marinus*	*EmxA*	GenBank	ACS91460	
	(sea lamprey)	*EmxB*	GenBank	ACS91461	
	Lethenteron japonicum	*LjEmx (EmxA)*	DDBJ	BAB13506	
	(Japanese lamprey)	*EmxB*		AB935430	
	Eptatretus burgeri	*EmxA*		AB935431	
	(hagfish)	*EmxB*		AB935432	
Gnathostomata	*Homo sapiens*	*Emx1*	GenBank	AAH45762	
	(human)	*Emx2*	GenBank	AAK95496	
	Monodelphis domesticus	*Emx1*	Ensembl 70		putative pseudogene, Viktorin et al., 2009; excluded from molecular phylogenetic tree inference
	(opossum)	*Emx2*	Ensembl 70	ENSMODP00000011730	
		Emx3	Ensembl 70	ENSMODP00000039292	
	Gallus gallus	*Emx1*	Ensembl 70	ENSGALP00000025888	
	(chicken)	*Emx2*	RefSeq	XP_421783	
	Xenopus tropicalis	*Emx1*	GenBank	AAH74580	
	(tropical clawed frog)	*Emx2*	GenBank	AAI69182	
		Emx3	Ensembl 70	ENSXETP00000062911	
	Latimeria chalumnae	*Emx1*	Ensembl 70	ENSLACP00000008106	
	(coelacanth)	*Emx2*	Ensembl 70	ENSLACP00000016479	
		Emx3	Ensembl 70		manually curated from Ensembl Pre, contig AFYH01039541.1 and AFYH01039542.1 central sequence unknown; excluded from molecular phylogenetic tree inference
	Oreochromis niloticus	*Emx1*	RefSeq	XP_003447816	
	(Nile tilapia)	*Emx2*	RefSeq	XP_003441112	
		Emx3	RefSeq	XP_003447667	
	Danio rerio	*Emx1*	GenBank	AAO25957	
	(zebrafish)	*Emx2*	GenBank	AAH92713	
		Emx3	GenBank	AAI65920	
	Gasterosteus aculeatus	*Emx1*	Ensembl 70	ENSGACP00000006428	
	(stickleback)	*Emx2*	Ensembl 70	ENSGACP00000004194	
		Emx3	Ensembl 70	ENSGACP00000021610	
	Lepisosteus oculatus	*Emx1*	Ensembl 74	ENSLOCP00000011406	N terminal sequence was manually curated with Ensembl, LG4: contig AHAT01015466.1
	(spotted gar)	*Emx2*	Ensembl 74	ENSLOCP00000011841	
		Emx3	Ensembl 74	ENSLOCP00000014949	
	Scyliorhinus canicula	*Emx1*	GenBank	AAM78422	
	(small-spotted catshark)	*Emx2*	GenBank	AAM78421	
		Emx3	GenBank	AAM78420	
	Leucoraja erinacea	*Emx1*	SkateBase		manually curated from SkateBase, Contig1755490, Contig254768, Contig401454
	(little skate)	*Emx2*	SkateBase		manually curated from SkateBase, Contig711073, Contig1603618
		Emx3	SkateBase		manually curated from SkateBase, Contig1744807, Contig1616832, Contig33556
Cephalochordata	*Branchiostoma floridae* (amphioxus)	*Emxb*	RefSeq	XP_002610876	Putnam et al., 2008
Echinoderm	*Strongylocentrotus purpuratus* (sea urchin)	*Emx*	RefSeq	XP_783008	
Arthropoda	*Pediculus humanus* (human louse)	*Emx*	RefSeq	XP_002425514	
