# Supplementary material for: Reevaluating Emx gene phylogeny: homopolymeric amino acid tracts as a potential factor obscuring orthology signals in cyclostome genes
Source: BMC Evol Biol. 2015 May 4;15:78. doi: 10.1186/s12862-015-0351-z (PMC4464114; doi:10.1186/s12862-015-0351-z)
Supplement: Additional file 2: Table S4. — Pdzd8 sequences used in this study. This table includes accession details of the sequences employed in phylogenetic tree inference for Figure 4b. [file 12862_2015_351_MOESM2_ESM.pdf]

Additional file 2 (Table S4). *Pdzd8* sequences used in this study

	Species	Database	Accession ID	Notes
Cyclostome	*Petromyzon marinus* (sea lamprey)	Ensembl	ENSPMAP00000007079	supplemented with ESTs
Jawed vertebrates	*Homo sapiens* (human)	Refseq	NP_776152	Ensembl ENSP00000334642
	Monodelphis domesticus (opossum)	Refseq	XP_001377292	Ensembl ENSMODP00000011737 has longer N-terminal region
	Gallus gallus (chicken)	Refseq	XP_426541.2	Ensembl ENSGALP00000042332
	Xenopus tropicalis (tropical clawed frog)	Refseq	NP_001072294.1	
	Latimeria chalumnae (coelacanth)	Ensembl 70	ENSLACP00000015340	N-end was predicted with support of an NCBI entry AFYH01067785.1
	Oreochromis niloticus (Nile tilapia)	Refseq	XP_003455037	Ensembl ENSONIP00000011508
	Danio rerio (zebrafish)	Refseq	XP_689774	Ensembl ENSDARP00000108510
	Lepisosteus oculatus (spotted gar)	Ensembl 74	ENSLOCP00000011824	
	Leucoraja erinacea (little skate)	SkateBase		manually curated from SkateBase (supplemented with ESTs)
Echinoderm	*Strongylocentrotus purpuratus* (sea urchin)	Refseq	XP_791331.3	
Arthropod	*Pediculus humanus* (human louse)	Refseq	XP_002430619	
Annelid	*Capitella teleta* (polychaete worm)	GenBank	ELT94329	
