# Supplementary material for: Reevaluating Emx gene phylogeny: homopolymeric amino acid tracts as a potential factor obscuring orthology signals in cyclostome genes
Source: BMC Evol Biol. 2015 May 4;15:78. doi: 10.1186/s12862-015-0351-z (PMC4464114; doi:10.1186/s12862-015-0351-z)
Supplement: Additional file 3: — Data S1. Manually curated Emx sequences of spotted gar, coelacanth and little skate. Sources of the sequences before curation are shown in Additional file 1: Table S1. [file 12862_2015_351_MOESM3_ESM.pdf]

Additional file 3 (Supplementary Data 1)

spotted gar Emx

>spotted_gar_Emx1
atgtttccgcctgcagcgaagcgctgtttcacaatcgagtccttggtggccaaggaaagtcctctga
ccgccgaggaacccatcagacccacagctctcagctactccaacccaacagatgccttcatgacggg
gttccagggccaggcgggcaggtccctgtactctggtccggagctggtcttccccgagacggtgaac
cacccgtctttgactgtgcacccccaccagctcgggtcttcgcatctgcagcacccgcactcgttct
tcggaacccagcaccgcgatcccattaacttttacccttgggttttacggaaccgattttcggaca
cagatttcaaggaaatgatgtttcccaggacgggctgttgctgcacggcccctttgccaggaaaccc
aaacggatccggaccgccttttccccgtctcagctcctgcggctagagagagccttcgagaagaacc
attatgtggtgggggccgagaggaagcagctagccaacagcctcagcctctccgaaacccaggtgaa
ggtgtggttccagaacaggaggaccaagtacaagcgccagaagctggaggaggagggcccagagtgc
cagcagaagaagaagggcacccatcacatcaacaggtggagaatcgccaccaaacaggccagctctg
aggacattgacgtcacgtccgatgactag

>spotted_gar_EMX1
MFPPAAKRCFTIESLVAKESPLTAEEPIRPTALSYSNPTDAFMTGFQGQAGRSLYSGPELVFPETVN
HPSLTVHPHQLGSSHLQHPHSFFGTQHRDPINFYPWVLRNRFFGHRFQGNDVSQDGLLLHGPFARKP
KRIRTAFSPSQLLRLERAFEKNHYVVGAERKQLANSLSLSETQVKVWFQNRRTKYKRQKLEEEGPEC
QQKKKGTHHINRWRIATKQASSEDIDVTSDD

coelacanth Emx

>coelacanth_Emx3
atgttccaacctgcaaccaaaaaatgtttcactatcgagtcgctggttggtaaggacactactagtt
cttcagctggggatgagcttataagacctacggctttaaaactcgccgactctgtctatccacctcc
ttttgggacctgttatcagagtagcgcgatgacgtttttttagttgcccggatctggtgtttcccgag
tcggccacacatgcatcgagccccgctgcttccctccaccaacagcacctccctgctcagcccttct
tttcaccgcatcaaggggaggctttgagctttttcccttgggtactcagaaacagatatctggggca
cagatttcaaggcaannnnnnnnnngtgaaggtgtggtttcagaatagaaggactaagcataaaagg
cagaaactggaagaggagtctccagaatcccaacagaaaaggaagggcggtcagcatataaaccgtt
ggagaatagctaccaggcagagcagtcctgatgacattgacgtgacctcagaggattaa

>coelacanth_EMX3
MFQPATKKCFTIESLVGKDTTSSSAGDELIRPTALKLADSVYPPPFGTCYQSSAMTFFSCPDLVFPE
SATHASSPAASLHQQHLPAQPFFSPHQGEALSFFPWVLRNRYLGHRFQGXXXXVKVWFQNRRTKHKR
QKLEEESPESQQKRKGGQHINRWRIATRQSSPDDIDVTSED

little skate Emx

>little_skate_Emx1
atgttccacgcgggcaccaagcgctgcttcacgatcgagtcgctggtggcgaaggagaacagcccgc
tgtcgccggacgagtcgctccgaccggccgctctcagctacccgaccagcgccgacggcttccccaa

cgccttccaggcgcccggccgctccctgtactcggggccggacctgatgtttcaggaggcagtcgct
cacccggcgctgcccgttcaccacccgcacccgctcagctcgcatcacctccagaccccgcacccct
tcttctcgcctcagcaccgcgacccccctcaatttctacccctgggtcctcaggaaccggctgttcgg
acacaggtttcaagggccggacggtgcgcaggagacaatgctacttcacggcccgtttgcccgcaag
cccaagcggatccgcacggccttctcaccgtctcagctgctgcgtctcgagagggccttcgagaaga
accactatgtggtgggagccgagaggaagcagctcgctggcagcctcagcctgtccgagacacaggt
gaaggtttggtttcagaacaggagaaccaagtacaagcggcagaagttggaggaggagggtcccgac
tcgcagcagaagaagaaaggttcacatcacgtcaaccgctggagactcgccaccaagcagtccagcc
ccgaggatattgacgtcatctcggaagactag

>little_skate_EMX1
MFHAGTKRCFTIESLVAKENSPLSPDESLRPAALSYPTSADGFPNAFQAPGRSLYSGPDLMFQEAVA
HPALPVHHPHPLSSHHLQTPHPFFSPQHRDPLNFYPWVLRNRLFGHRFQGPDGAQETMLLHGPFARK
PKRIRTAFSPSQLLRLERAFEKNHYVVGAERKQLAGSLSLSETQVKVWFQNRRTKYKRQKLEEEGPD
SQQKKKGSHHVNRWRLATKQSSPEDIDVISED

>little_skate_Emx2
atgttccagccaacacctaagcggtgttttaccattgaatctctagtagccaaggacagtccactgc
ctgcttcgagatcagaggaacctataaggccagccgccctcagctatgcgaactctagccccgtcaa
tccattcctgaacggcttccacaccagcggcagggcaatctacaacccggagctggtgttcgctgac
gcggtctctcatcaaaccaacacggcagtaccggttcacccagtccctccacatgctttagcagcgc
accctcttcaatcgtcccattctccacatcccttatttgcatcacaacaaagagatccttcgagttt
ctatccatggctaatacacaggtacagatatctgggacacaggtttcaaggaaacgagactagtccg
gaaagcttttattgcacaatgcgctcgccaggaagcccaaacgcatccgcacggccttctcaccgt
ctcagctgctgagactcgagcacgcctttgagaaaaatcactatgtggtgggcgcagagaggaaaca
actggcccacagtctcagtttaacagaaactcaggtaaaagtgtggttccagaatagaagaacgaaa
tttaaacggcagaagttggaggaggaagggacagatgcacaacagaagaaaaaggggacccatcatg
tcaatcgttggagacttgcgaccaaacagtcgagccctgaggagatcgatgtcacctcggacgatta
g

>little_skate_EMX2
MFQPTPKRCFTIESLVAKDSPLPASRSEEPIRPAALSYANSSPVNPFLNGFHTSGRAIYNPELVFAD
AVSHQTNTAVPVHPVPPHALAAHPLQSSHSPHPLFASQQRDPSSFYPWLIHRYRYLGHRFQGNETSP
ESFLLHNALARKPKRIRTAFSPSQLLRLEHAFEKNHYVVGAERKQLAHSLSLTETQVKVWFQNRRTK
FKRQKLEEEGTDAQQKKKGTHHVNRWRLATKQSSPEEIDVTSDD

>little_skate_Emx3
atgttccagcctccggccaagagatgcttcaccatcgaggctctggtaggcaaagacaacgcgtcca
ctctgggggatgaaccgctccgaccgacagctctgagatacgccgactcgctgcacgcctcccactt
cggaagtggctttcagaacgccggcaggactctctacagcagccccgagctcttgtttcccgacccg
gtaacccacgcggccagccccgctctctcaattcacccaggcaacgcacaccatctgtccgcacagt
ctttctttgcgccccagcagagagaagcactaagtttttacccatgggttttaaggaacagatacct
gggtcacagatttcaaggtggcgagggaggagcggagagcctacttctccacggccctttcaccagg
aagcctaagcggattcgaacagcgttctctccgtcccagctcctgagactagagagagctttcgaga
aaaaccactacgtggtgggcgccgagcgaaaacaattagcaagcagcttgtgtctcacggaaaccca
ggtaaaggtgtggtttcagaacagaaggaccaagtacaaacgacagaagctggaggaagagagcccc
gacacgcagcagaaacgaaaagggacacaacacgtgagcaggtggagaatggccaccaggcagacca
gcccggaggacattgatgtgatttcagaagattag

>little_skate_EMX3
MFQPPAKRCFTIEALVGKDNASTLGDEPLRPTALRYADSLHASHFGSGFQNAGRTLYSSPELLFPDP

VTHAASPALSIHPGNAHHLSAQSFFAPQQREALSFYPWVLRNRYLGHRFQGGEGGAESLLLHGPFTR
KPKRIRTAFSPSQLLRLERAFEKNHYVVGAERKQLASSLCLTETQVKVWFQNRRTKYKRQKLEEESP
DTQQKRKGTQHVSRWRMATRQTSPEDIDVISED
