# Supplementary material for: Reevaluating Emx gene phylogeny: homopolymeric amino acid tracts as a potential factor obscuring orthology signals in cyclostome genes
Source: BMC Evol Biol. 2015 May 4;15:78. doi: 10.1186/s12862-015-0351-z (PMC4464114; doi:10.1186/s12862-015-0351-z)
Supplement: Additional file 4: — Data S3. Manually curated Pdzd8 sequences of coelacanth, little skate and sea lamprey. Sources of the sequences before curation are shown in Additional file 2: Table S4. [file 12862_2015_351_MOESM4_ESM.pdf]

Additional file 4 (Supplementary Data 3)

coelacanth PDZD8

```
>coelacanth_pdzd8
atgatttatttaattctcctttcggtgtttgcgggggctttctttacgctgctgttggagtttttgt
tactgtatagaaggaagcctgagcctgtgcccagaactgtgcaatatgtcaagccagtgcctgatcc
attactgaaggattactttaataataataaaaatagtgattcaggccagcagcagcaggattttgct
gcacctacgccttcaaaacatcaagaagctgctggcgtgaaaccgcaaccggaatctgcaaacccct
tcaaagcggagagctgtcattctctaaatgcgatatttttatttttgttcagggagcttagagatac
tcctttggttaggcactgggtgaccaagaagattaaggtcgaatttgaagagctgctgcagactaaa
actgctggaaggttactagagggggctcagtttaagggatatttctttgggcaatgctttgcctgtat
ttaagacggctcaacttctcaaacctgtggcttgcaatgaagatggaatgccagaggagctgaattt
tgagatagacattgagtacaatggagggttccacctggccattgatgttgatctggtctttgggaaa
tctgcttatctctttgtgaagatgactagagtggtgggcagactgaggttgcagttcacacgtctac
cttttacccactggtcattctcgtttttggaggatccccttatagatttcgaagtgaagtctcagtt
tgaagggaggcctatgcctcagttaacctctattattgtcaatcagctgaagaaagtcgtgaagagg
aagcatactttgccaaattacaaaatcagnnnnnnnnnnnnngttgagttgatcaaaggaaattcac
aaagcgtaggactgactttccgtcaaattcaagctagtgatggagatgcagggcatgtctgcattga
aactgtaactccaaattcacctgctgcagttgctgaccttcaaaggggagatcgtctcatagctatt
ggaggtactaaagtaacttcatctgttcaagtactgaagttaatcaagcaggctggtgacagggtca
tggtatattatgaaagacctgttggccatcagaatcaacatggatcagagagttttggccagctgga
agatactggttatatgttgcaatcatgtccccaaagttatgaagaagatactgtgagcataactaac
ttagacagtggtgatggcaaagatgtggactctgaatttgaagagttagtgtgtgaagtaaagccaa
caaatgagttcaaagaggagccattacccacagtcacccaaagtccaaaacggtcagtagccacact
tgctgttaaaccattaggaactatatctcccattctaaatcgcaaactaaatttggtgaattatcca
gcaccccttaaaccacagcaaaaagaaggtgccaagcctacacaatataaaggttctgagcatcaag
agggaacaccacaaccaaacaaaacatcacaaggatctaccaataagcctcctgttccccctagacc
tcatattaaacctaatttggcttcctctgagagccagtgtgtgctagagacgggagacacatttgta
gagaaaactgaaaagaccccaactccttcaagtaatggagagaagtcagcagagaaaacgtcagcaa
aaaatattgatcttattgaagacttaggaacatcaaagacatgtacaagtaaacaagaagcagttaa
agataagatttctgaaagttcgtgcagcacaaaagacagcatggaagaccgtcagacatgggagtct
tcagaaataccatatcgtaatcgatatggcaaatggaacaaagctacttgcttatttgaggtagaaa
atcatcataagtatcttaatgttgcactgtggtgtaaagacccatttaaagtgggggatcttatctg
tttagggcatataagtataaagcttgaagaagttgccttggaatgtttatctacatcatcaatggaa
taccaaactacattcaggttaaatgcacctgaaccaaaagctgtggtcagtagaactgctctgagaa
tcctaagtactcataaaggatttaatgaaaaactttgttatggagatatcactttgaatttcacata
cttgaaagaaggtgaatcggaaaattcaagttttgtgatagaaaaagaaagggaatgtaatgtacaa
gaagaagtccctgtattccagaaagaggaacctaatttagggcaaatgtttttatggaaaacaagc
ataattttcaagatactcagtttcagaatccaacttggtgtgatcactgtaaaaagaaggtgtggac
taaagcagcatctcagtgtatgctttgtgcctatgtgtgtcataaaaaatgccaagaaaagtgtcta
gcagaaaatccattttgtgttgctactgaaagaagagttgattttgaatcgaagtcaactataaaca
gaaccactggtttaaccaggcatattttaaacacaagttcacgtttattgaacctgcgacaagcgcc
aaaggctcgcctagctgaacaaggggctgaggctgtagaaccatcaccaaagcacactccaaacact
tcagataatgaaagtagtgacactgagacatatagtggcagcagtccttctaaacgagcaacaagta
caggcataaagttagcaagaaaagaaggggtctcgatgacagtgtttttattgctgtaaaagaaat
tggtagagatctttatagaggcttatctactgaagaaagatctcagaaattagaatttatgttagac
aaactgcagaatgaaatagaccaagaactagagcataacaatttactgttaaaagaagagaagggg
ccactgatgcaagaaagaaatcgctgctttgtgcagcattatctaaatcaagtgaacgactccaggc
tttaacacttcttgtgatccactatcgagcaggcattgaagacctggagtcccttgaaagtatgtca
tcagatcagcaatcaagaaaaacaagatgttcagaagaatcatctctaactacagacgtgtatgaaa
atgaggttccaaatcagatggacactcaaacatttaataatatacccgatgagcagatttttgatga
```

atctttctcatag

>coelacanth_PDZD8
MIYLILLSVFAGAFFTLLLEFLLLYRRKPEPVPRTVQYVKPVPDPLLKDYFNNNKNSDSGQQQQDFA
APTPSKHQEAAGVKPQPESANPFKAESCHSLNAIFLFLFRELRDTPLVRHWVTKKIKVEFEELLQTK
TAGRLLEGLSLRDISLGNALPVFKTAQLLKPVACNEDGMPEELNFEIDIEYNGGFHLAIDVDLVFGK
SAYLFVKMTRVVGRLRLQFTRLPFTHWSFSFLEDPLIDFEVKSQFEGRPMPQLTSIIVNQLKKVVKR
KHTLPNYKIXXXXVELIKGNSQSVGLTFRQIQASDGDAGHVCIETVTPNSPAAVADLQRGDRLIAIG
GTKVTSSVQVLKLIKQAGDRVMVYYERPVGHQNQHGSESFGQLEDTGYMLQSCPQSYEEDTVSITNL
DSGDGKDVDSEFEELVCEVKPTNEFKEEPLPTVTQSPKRSVATLAVKPLGTISPILNRKLNLVNYPA
PLKPQQKEGAKPTQYKGSEHQEGTPQPNKTSQGSTNKPPVPPRPHIKPNLASSESQCVLETGDTFVE
KTEKTPTPSSNGEKSAEKTSAKNIDLIEDLGTSKTCTSKQEAVKDKISESSCSTKDSMEDRQTWESS
EIPYRNRYGKWNKATCLFEVENHHKYLNVALWCKDPFKVGDLICLGHISIKLEEVALECLSTSSMEY
QTTFRLNAPEPKAVVSRTALRILSTHKGFNEKLCYGDITLNFTYLKEGESENSSFVIEKERECNVQE
EVPVFQKEEPNLGQMFFMENKHNFQDTQFQNPTWCDHCKKKVWTKAASQCMLCAYVCHKKCQEKCLA
ENPFCVATERRVDFESKSTINRTTGLTRHILNTSSRLLNLRQAPKARLAEQGAEAVEPSPKHTPNTS
DNESSDTETYSGSSPSKRATSTGIKLARKEGGLDDSVFIAVKEIGRDLYRGLSTEERSQKLEFMLDK
LQNEIDQELEHNNLLLKEEKGATDARKKSLLCAALSKSSERLQALTLLVIHYRAGIEDLESLESMSS
DQQSRKTRCSEESSLTTDVYENEVPNQMDTQTFNNIPDEQIFDESFS

little skate PDZD8

>little_skate_pdzd8
atgatttatataatcctgttgtctgctctggccggggctctgtgcgtggtggtgctgcagattttat
tgctgtaccggaacaaacccgaggctgtgccccggcatgtgcagtatgttaagcccgtggtcgagcc
ttcgttgaaggactatctcagcggcgccaaggagcggccggcttccccggaatcgaccagctcggcg
cccgggaacggcggcagcgacaagtcggacagctgccactttctcaacgccatctttctgtttctct
ttcgggagctccgcgacaccccgattgtccgccactggctgaccaagaagatcaaagtggagttcga
ggagctgctgcagacgcggacggcgggccggctactggaagggctgagcctgcgagacatctccctg
ggcaacgctctgcccgtgttgcggagcgcgcgcccgctccaaccgtcgcccgtcaccgccaacagca
gcggcggcggcggcaccagcaatagcagcggcgggcaggacgacgaggccggcggcgtgccagacga
gctcaacttcgaggtggagctcgagtacaacgggggcttccacctcgccatcgacgtggacctggtc
tttggcaaatcggcctacctattcgtcaagatgaggagggtggtgggccggctgcggctgcagttca
cccggcgccctttcacgcactggtccttcgccttcatggacgaccctttcatcgacttcgaggtcaa
atcgcagttcgagggccggcccatgccccagctcacctctatcatcgtcaaccagctcaagagggtc
atcaagcggaagcataccttgcccaactacaagatcaggtacaaaccattctttccataccaagttc
ttcctgccacagccgatacagtgaattgtgacctctatttacaacacgcgaacatgactgagggacg
cctaaaagtcactttggtagaatgcagcaggttatttatcctgggattgtatgaccgttctgaaacc
attcactgtactctggagttaagtagcgatgtgtggaaagagaagaccagaagctccatcaaaacgg
tagagctaattaaaggaaattcacaaagcgtaggactggctttccgccaaatccagggcaacgatgg
ggatgcagtgcatgttaccgtggaaactgtgaccccaaactcgccggccgcagctgctgacctccaa
aagggagatcgactcattgcaatcggaggaaccaaggtgacatcatctgtacatgttttgaagctta
taaagcaagctggtgacaaagtattggtttattacgaaagacctgttggtggacatcggaatcaaca
atgtggctcactccaggagaactgtggccagcttgaggacactgggtttagtgcacagtttggacaa
catgcatttgatgatgatggtattttagataatgttgacggtaaagacttagattctgaattcgaag
aattagtatgtcctgaaatcaaaccaccgaccataatcgtaaaggaggattttgcaccatctaatct
aagtccaaagcgttcagtagctacacttgccaccaagccactaggaaccatatcccccattctgaat
cgtaaattaaatttggggaattaccagattcccctgaaatcacagcaaaaagacgcagctaagtttg
cccagccaaaatgtttagacgtccctgatggagcacagcagccaagtaagccagcattaggatccac
aaataaacctccggtgcccccaaggcctcagataaagataatttcagctgcttctgaagcccagaac
ctgtctgaatctgcagacacagctacagaaaaacttgacaaatcggcaacatccatcaatagtgcag

agaaaagtattgaaaaaggtgtcagtaaaaatctagatcagatggaagatgcatctcagtccaggcc
aacagctagtaaacaagattccaaggagaaattaccagaaactacctgcagagtgcgcgatggggca
gaagggaataaaatctgggagtcaccagagatatcctatcgcaagagatttgtgaagtggaacaagg
caacgtgcgtgtttgacgttgaaggacgccacagatacatcaacgttgccgtgtggtgtaaagaacc
ttttaaatcaggtgaactcatgtgtttaggccatgcaagtataaaacttgaagagatagccatggaa
tgtctttgcactgcatcaatggagtttcagacaacctttcggcttaatgctcctgaaccaaaagctg
ttgtcagtagaactgcattacgaaatctaagcactcataagggattcaatgagaaactgtgctatgg
tgacgttacactaaattttacctatttaaaagagggtgatacagaaaactctagcattcaaatagaa
agggaaaaggaagatcatttacaggaggatattcctgtcatcattaaggaggaaactgatccctcgt
gtatggacaataagcacaatttccaagatactcagtttcagaatccaacctggtgtgattactgtaa
aaagaaagtctggaccaaagctgcttctcagtgcatgaactgtgcatatgtgtgtcataaaaagtgt
caggataagtgtctggcagaagcacttcttccaggtctcacagagagaagagctgatttggaagcca
agccgtcctttaacaggtcaacagggttaacacggcatatcatcaacaccagctcacgcttgctgaa
cctgagaccaggacaaagagctcgtcttgcggatcctagtgtagatttggtggagccttcacccaaa
caaactccaaatacatcagacaatgaaagcagtgacacagagacgtacagtgccagtagtccatcca
aacgggtggcttcttccatgggtaacaagttatcagtgaagaaagacggtggtctcgacgatagtgt
attgattgctgtaaaggagattggtcgagacctgtatcgtggattaccagccgatgaacgatttcaa
aaactgtacttgatgtcagaaaagctacagaatgaaatagagttggaactggaacagaattatgctc
tgcagaaagaagaaaaggattgcacagattctaaaaagaaatgtcatttgtctgctgctatttcaaa
gtcaattgaacggctacaagcccttacactgctcacaatccactacaaagcaggaattgatgattta
gaatttagcgactttgcatcttctgaacaacaggcaaaaaaagcctcaaagcttacagatgatattt
tatccctaacaacagacgatattgatgttggcagtcaggcagatgtgcaaccacttactgaagaatc
aaatgagcaaactatcgatgaaactgaatcaatttcttga

>little_skate_PDZD8
MIYIILLSALAGALCVVVLQILLLYRNKPEAVPRHVQYVKPVVEPSLKDYLSGAKERPASPESTSSA
PGNGGSDKSDSCHFLNAIFLFLFRELRDTPIVRHWLTKKIKVEFEELLQTRTAGRLLEGLSLRDISL
GNALPVLRSARPLQPSPVTANSSGGGGTSNSSGGQDDEAGGVPDELNFEVELEYNGGFHLAIDVDLV
FGKSAYLFVKMRRVVGRLRLQFTRRPFTHWSFAFMDDPFIDFEVKSQFEGRPMPQLTSIIVNQLKRV
IKRKHTLPNYKIRYKPFFPYQVLPATADTVNCDLYLQHANMTEGRLKVTLVECSRLFILGLYDRSET
IHCTLELSSDVWKEKTRSSIKTVELIKGNSQSVGLAFRQIQGNDGDAVHVTVETVTPNSPAAAADLQ
KGDRLIAIGGTKVTSSVHVLKLIKQAGDKVLVYYERPVGGHRNQQCGSLQENCGQLEDTGFSAQFGQ
HAFDDDGILDNVDGKDLDSEFEELVCPEIKPPTIIVKEDFAPSNLSPKRSVATLATKPLGTISPILN
RKLNLGNYQIPLKSQQKDAAKFAQPKCLDVPDGAQQPSKPALGSTNKPPVPPRPQIKIISAASEAQN
LSESADTATEKLDKSATSINSAEKSIEKGVSKNLDQMEDASQSRPTASKQDSKEKLPETTCRVRDGA
EGNKIWESPEISYRKRFVKWNKATCVFDVEGRHRYINVAVWCKEPFKSGELMCLGHASIKLEEIAME
CLCTASMEFQTTFRLNAPEPKAVVSRTALRNLSTHKGFNEKLCYGDVTLNFTYLKEGDTENSSIQIE
REKEDHLQEDIPVIIKEETDPSCMDNKHNFQDTQFQNPTWCDYCKKKVWTKAASQCMNCAYVCHKKC
QDKCLAEALLPGLTERRADLEAKPSFNRSTGLTRHIINTSSRLLNLRPGQRARLADPSVDLVEPSPK
QTPNTSDNESSDTETYSASSPSKRVASSMGNKLSVKKDGGLDDSVLIAVKEIGRDLYRGLPADERFQ
KLYLMSEKLQNEIELELEQNYALQKEEKDCTDSKKKCHLSAAISKSIERLQALTLLTIHYKAGIDDL
EFSDFASSEQQAKKASKLTDDILSLTTDDIDVGSQADVQPLTEESNEQTIDETESIS

sea lamprey PDZD8

>sea_lamprey_pdzd8
atgttttacgtgatagtcttttccgccttgttcggcatcgtctttaccttcatgctcgaagttttca
tctattaccgcctgggtttaaagccggccaagtctgttgtcgtaggcccgagggagcctacggtctc
gtctagacccggtggcggggaggcgaagggggtccccggggcgcaggggcaggggcaagtcacctct
tccccggacggtctcagtgccggcgagcctccgcccggaacgccagagagctgtgacttcttgaacg
tcctcttcctcttcttgttccgcgagctcagggacacgggcactgtgcgcaggtggctcatcaagaa

gattcgcgtggaatttgtggagctgctgcagagcaagacagtcggccggctgattgaagggctgagt
ttgcgcgatgtgtcactcggtgacactttgcccgtcttcagctccgtggtgatgcatgaaccagacc
ctgttcctgaaaaaaatgtccctgagcaattggatctcgaggtcgatgtggaatacagcgggggatt
tcacctggcggtggacgtcgacctggtgttcggcaagtccgcgtacctgtttgtgcgtgtggcgcgt
gtggctggccgcgtgcgcctgcgcttcagccacctgcccttcacgcactggtccttcgcctttatcc
gcgagcccgagcttgaattcgacgtcgagtcacggctggagggacggccgttgccccagctgacggc
cataatcgaaaggcagctgcgcagtgccatctgccgcaagcacacgctgcccaactacaaagtcaga
tccaagccgttctttcctgttcgagttgttccacgcgacatcgacctgtgcatgcaggacagcaagt
tgagcgagggtaggctgagggtggcgcttctggaatgcagcaggctgtacataccaggatcccagga
ccgggaagttgctgtgcactgcacattggagctgagtgctggccagtggaaagaacgagaaaggagt
gccgttcgagaggttgagatcgtgaaggtcggcacagcccccatcggcctcagtttccgccagaccc
agtccacagacggggagtcaagccaggtcctcgtggaggcgatagcggcaaactcccccgcgtccag
tgcaaacatacgtcgcggggaacggctggtgacaattggaggaacgaaagtggcaacttcaagccag
gcgtcgaaactgatcaagcaagcaggggagcgagtaattatcagcttggagcggacgtttggaggcg
tgcagccgcctcctggcagcgcctcgggccctctgccccacggcatgcatgtggcagatggttctgc
tattatcccagatgacgattcttccaaagacacagtctccctggggtccgttgaaagcaacgacgcg
agagagggagattccgacgtggacgagggtgaatgtggcgacggtttccccgagagcctgcgctcga
aagaggaaccgcctccgtcgctgctttctgttccaccgaccaaggatgagacaccgcagccgccctc
cacggccagccccagcaagcggcgcacaatggccaccctcggcagcatctcgccgatccttagccgc
cggctgcagcaatcctcgccgctccgttctctacacaagggctatgacgcggccaaattagggagca
ccacacctgcacagccagatggagagaaggtggctccgtctgcttctgctaagcccggcctagtgaa
tcagagcggtcggccgcccccgataccgcccaggccgcagcttagggccaccagcagtgcaccgctt
gggagccttcagtcgctcgcggaaacaggcgatgcaagcacgctcaagcccacggacaagggagggt
ccatggaacaagtggaacaagagaaactcggcgacgcgacggacagcggagcggtcggcgctagcgg
cctggcgtgtgcggaggagtgcacggatgagctgtgtgtcacgcaggagatggtataccactccaac
agggccgtgtggccggctcgcgccgcctcgcacaccttcgacgttgagagtcatcaccgctccctga
cggtcgctgtttggcgcaaggacccgctcaagccggactccctgcgctgcctgggctatgccagcgt
gccactcatggagatagcagcggagtgcctggccaccgcgtcctatgagcacgaggaggtgttcccg
ctgagcgctcccgaacccagggccaccgccaaccgcaccgccatgcgcaacgccaacgcaaaggaga
agcagaatctggagcaaccatattatggtgacgttcggctgcggtttgcctactgggtcgacgaagg
cggcgatggtttgacatcgtcgagcgcaaacgtggacgaagaccgcgatcttttcacaagagccgag
gaggttggccccgtgcccgccggggtgcgcgattgggacagcggcccacccgcagcacctccgccac
cattgccaccactgccgccatcgtcggggatgggtggagcggcgcgccacagcttctgcgacaccca
attccagaaccccacctactgcgactactgcaagaagaaggtgtggaccaaggcggcgacgcagtgc
tcgcgctgtgcctacgtgtgccacaagaagtgccaggagcgctgcttggaggagagcgagagcgggc
gctgcatgccgagggagagcagggagggagcccgcgagggcggcgccgctgggagcggcgccgccgc
tgccggcagtggtggcggcgacccaccggctcgcctctcaaacctcaagcagtcggtagcgatggca
cggtcccgtctgacgctggccgtgcccaggccgctgcggcaggccgggagccggctccgagccagtg
aggaggttgtcgtcgggccaacgcagtcgggggaacgtcggacaacgagagcagcgacaacgaggg
agggagcagcgggagtgcggtgggtcccactgggcagccatccgcttctgcgcgtgcgatggagcag
gcgtccctgccgggccggctcgaggacagcgtgtacctggccgtcaaggagatcggccgtgaaatct
tccgcggcatgccggccgaggagcggaggtccaaactggagcagatgctggacaagctgcagcagga
ggtggaggccgagctggagcagagaaacacgctcgagagggaggagcgccaggcgtgcgccgccggc
tcggatgcgaagaaggtggcgaacgtgacgcactcgttgcgcaagtccacggagaggctgcaggcac
tgacgctgctgtccatccactaccgcgctgggatggaggaagaggatcagtcggaagttggctgcgt
accgacgggtggcccttcgactgcacaccggctcccacaacctccccaggcggtgtcctccgtgttg
acccagctcccaccggacgtgcccttgcacccggcggaatcggagcagttccatacgctgcaggatc
cttcgttattcccgtcagcgccgcgggacgacctagcggtaattcagggagcagtggcagaggtgtc
tggggcgacgtgggaggaaggggatgcggaggaggcatgtgcagccgcgccggaggtttgcgaagac
ccgagtgaaactgggtttgcgttggacgtggaggaagatgatgaggaagttggagcaaagctgctgg
cttaa

>sea_lamprey_PDZD8
MFYVIVFSALFGIVFTFMLEVFIYYRLGLKPAKSVVVGPREPTVSSRPGGGEAKGVPGAQGQGQVTS
SPDGLSAGEPPPGTPESCDFLNVLFLFLFRELRDTGTVRRWLIKKIRVEFVELLQSKTVGRLIEGLS

LRDVSLGDTLPVFSSVVMHEPDPVPEKNVPEQLDLEVDVEYSGGFHLAVDVDLVFGKSAYLFVRVAR
VAGRVRLRFSHLPFTHWSFAFIREPELEFDVESRLEGRPLPQLTAIIERQLRSAICRKHTLPNYKVR
SKPFFPVRVVPRDIDLCMQDSKLSEGRLRVALLECSRLYIPGSQDREVAVHCTLELSAGQWKERERS
AVREVEIVKVGTAPIGLSFRQTQSTDGESSQVLVEAIAANSPASSANIRRGERLVTIGGTKVATSSQ
ASKLIKQAGERVIISLERTFGGVQPPPGSASGPLPHGMHVADGSAIIPDDDSSKDTVSLGSVESNDA
REGDSDVDEGECGDGFPESLRSKEEPPPSLLSVPPTKDETPQPPSTASPSKRRTMATLGSISPILSR
RLQQSSPLRSLHKGYDAAKLGSTTPAQPDGEKVAPSASAKPGLVNQSGRPPPIPPRPQLRATSSAPL
GSLQSLAETGDASTLKPTDKGGSMEQVEQEKLGDATDSGAVGASGLACAEECTDELCVTQEMVYHSN
RAVWPARAASHTFDVESHHRSLTVAVWRKDPLKPDSLRCLGYASVPLMEIAAECLATASYEHEEVFP
LSAPEPRATANRTAMRNANAKEKQNLEQPYYGDVRLRFAYWVDEGGDGLTSSSANVDEDRDLFTRAE
EVGPVPAGVRDWDSGPPAAPPPPLPPLPPSSGMGGAARHSFCDTQFQNPTYCDYCKKKVWTKAATQC
SRCAYVCHKKCQERCLEESESGRCMPRESREGAREGGAAGSGAAAAGSGGGDPPARLSNLKQSVAMA
RSRLTLAVPRPLRQAGSRLRASEEVVVGPTQSGGTSDNESSDNEGGSSGSAVGPTGQPSASARAMEQ
ASLPGRLEDSVYLAVKEIGREIFRGMPAEERRSKLEQMLDKLQQEVEAELEQRNTLEREERQACAAG
SDAKKVANVTHSLRKSTERLQALTLLSIHYRAGMEEEDQSEVGCVPTGGPSTAHRLPQPPQAVSSVL
TQLPPDVPLHPAESEQFHTLQDPSLFPSAPRDDLAVIQGAVAEVSGATWEEGDAEEACAAAPEVCED
PSETGFALDVEEDDEEVGAKLLA
