# Supplementary material for: Reevaluating Emx gene phylogeny: homopolymeric amino acid tracts as a potential factor obscuring orthology signals in cyclostome genes
Source: BMC Evol Biol. 2015 May 4;15:78. doi: 10.1186/s12862-015-0351-z (PMC4464114; doi:10.1186/s12862-015-0351-z)
Supplement: Additional file 5: Table S2. — ML analysis for validation of hagfish-lamprey orthology of EmxA and EmxB. This analysis was performed with exhaustive ML method as described in Methods. [file 12862_2015_351_MOESM5_ESM.pdf]

Additional file 5 (Table S2). ML analysis for validation of hagfish-lamprey orthology of *EmxA* and *EmxB*

Rank	Tree topology	log *L*	Δlog *L* ± SE	*P* -value		Supported hagfish-lamprey orthology
				1sKH	SH	
1	((((g1,g3),g2),(lampreyB,hagfishB)),(lampreyA,hagfishA),outgroup);	-1910.49	ML	1.00	1.00	*EmxA, EmxB*
2	((((g1,g3),(lampreyB,hagfishB)),g2),(lampreyA,hagfishA),outgroup);	-1911.33	0.84 ± 1.65	0.27	0.98	*EmxA, EmxB*
3	(((g1,g3),(g2,(lampreyB,hagfishB))),(lampreyA,hagfishA),outgroup);	-1911.33	0.84 ± 1.65	0.26	0.98	*EmxA, EmxB*
4	((((g1,g3),g2),(lampreyA,hagfishA)),(lampreyB,hagfishB),outgroup);	-1912.25	1.76 ± 2.31	0.21	0.97	*EmxA, EmxB*
5	(((g1,g3),g2),((lampreyA,hagfishA),(lampreyB,hagfishB)),outgroup);	-1912.25	1.76 ± 2.31	0.20	0.97	*EmxA, EmxB*
6	(((((g1,g3),g2),(lampreyB,hagfishB)),lampreyA),hagfishA,outgroup);	-1912.93	2.44 ± 2.91	0.19	0.94	*EmxB*
7	(((((g1,g3),g2),(lampreyB,hagfishB)),hagfishA),lampreyA,outgroup);	-1912.93	2.44 ± 2.91	0.19	0.94	*EmxB*
8	(((g1,g3),((lampreyA,hagfishA),(lampreyB,hagfishB))),g2,outgroup);	-1912.95	2.46 ± 2.58	0.17	0.96	*EmxA, EmxB*
9	((g1,g3),(g2,((lampreyA,hagfishA),(lampreyB,hagfishB))),outgroup);	-1912.95	2.46 ± 2.58	0.17	0.96	*EmxA, EmxB*
10	((((g1,g3),(lampreyA,hagfishA)),g2),(lampreyB,hagfishB),outgroup);	-1913.11	2.61 ± 2.87	0.19	0.95	*EmxA, EmxB*
11	((g1,g3),((g2,(lampreyB,hagfishB)),(lampreyA,hagfishA)),outgroup);	-1913.11	2.61 ± 2.87	0.19	0.95	*EmxA, EmxB*
12	((g1,g3),((g2,(lampreyA,hagfishA)),(lampreyB,hagfishB)),outgroup);	-1913.11	2.61 ± 2.87	0.19	0.95	*EmxA, EmxB*
13	(((g1,g3),(lampreyB,hagfishB)),(g2,(lampreyA,hagfishA)),outgroup);	-1913.11	2.61 ± 2.87	0.19	0.95	*EmxA, EmxB*
14	((((g1,g3),(lampreyA,hagfishA)),(lampreyB,hagfishB)),g2,outgroup);	-1913.11	2.61 ± 2.87	0.18	0.95	*EmxA, EmxB*
15	(((g1,g3),(lampreyA,hagfishA)),(g2,(lampreyB,hagfishB)),outgroup);	-1913.11	2.61 ± 2.87	0.18	0.95	*EmxA, EmxB*
16	(((g1,g3),(g2,(lampreyA,hagfishA))),(lampreyB,hagfishB),outgroup);	-1913.11	2.61 ± 2.87	0.17	0.95	*EmxA, EmxB*
17	((((g1,g3),(lampreyB,hagfishB)),(lampreyA,hagfishA)),g2,outgroup);	-1913.11	2.61 ± 2.87	0.17	0.95	*EmxA, EmxB*

This table includes only tree topologies with its standard error larger than the difference of log-likelihood from the ML tree (Δlog *L*).

Abbreviation: SE, standard error; 1sKH, One-sided Kishino-Hasegawa test; SH, Shimodaira-Hasegawa test; g1, gnathostome *Emx1*; g2, gnathostome *Emx2*; g3, gnathostome *Emx3*; cA, cyclostome *EmxA*; cB, cyclostome *EmxB*; lampreyA, lamprey *EmxA*; lamprey, lamprey *EmxB*; hagfishA, hagfish *EmxA*; hagfishB, hagfish *EmxB*.
