# Supplementary figures and images for: Reevaluating Emx gene phylogeny: homopolymeric amino acid tracts as a potential factor obscuring orthology signals in cyclostome genes
Source: BMC Evol Biol. 2015 May 4;15:78. doi: 10.1186/s12862-015-0351-z (PMC4464114; doi:10.1186/s12862-015-0351-z)

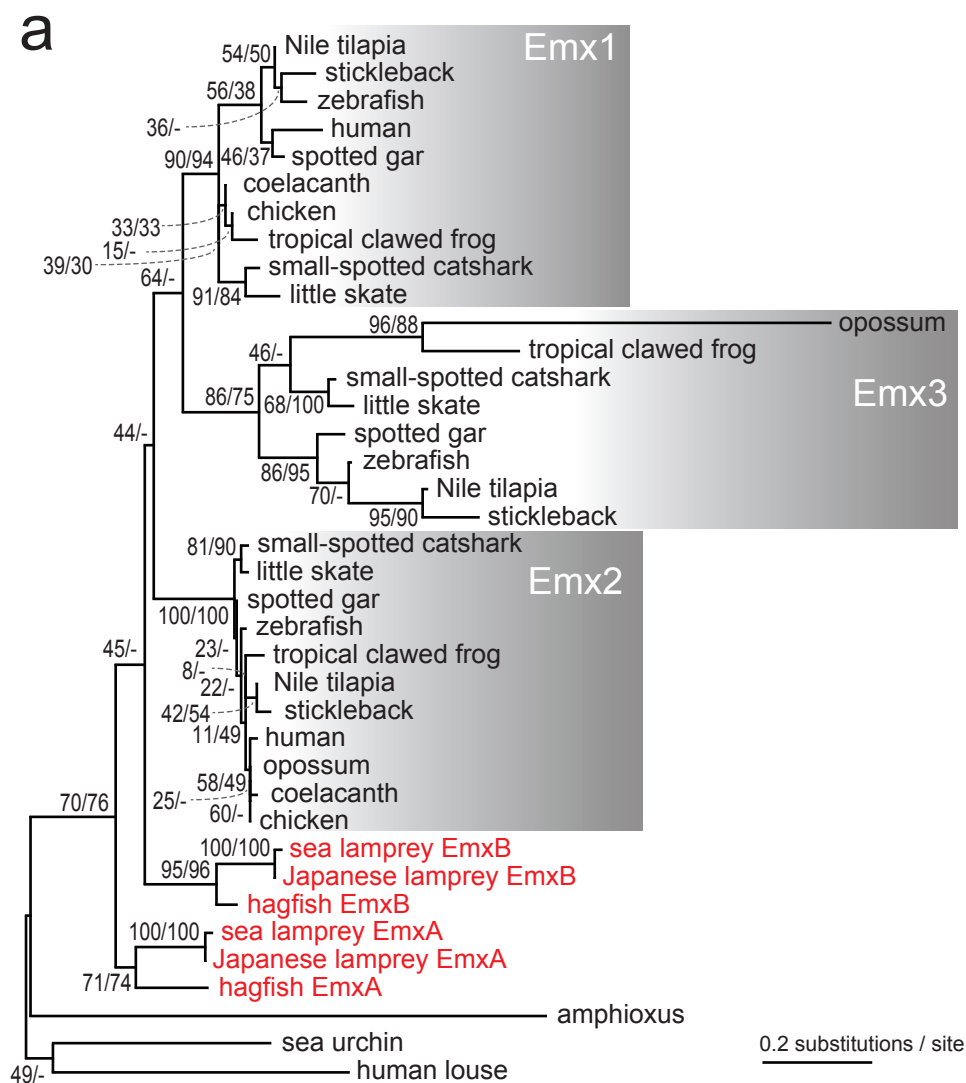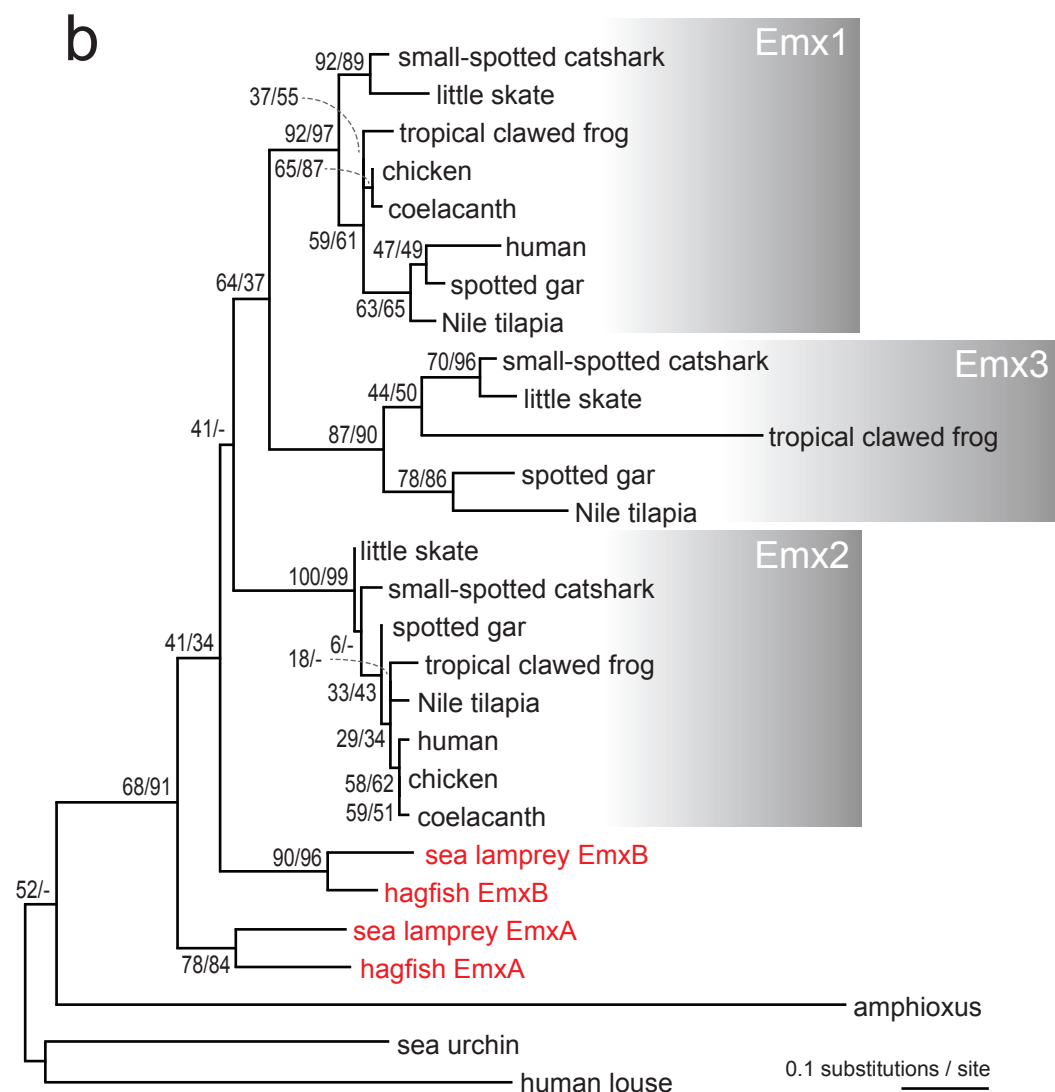

Supplement: Additional file 6: Figure S1. — ML trees for vertebrate Emx genes including both sea lamprey and hagfish. (a) ML tree with the enriched dataset with more vertebrate sequences. This ML tree was inferred using 130 amino acid sites in the multiple alignment in Additional file 7: Data S2, assuming the JTT model with the proportion of invariable sites taken into account (JTT+Γ4+I model) (shape parameter α = 0.65). (b) ML tree inferred with selected sequences. This ML tree was inferred using 132 amino acid sites in the multiple alignment in Additional file 7: Data S2, assuming JTT +Γ4+I model (shape parameter α = 0.58). The numbers at nodes indicate bootstrap probabilities with 100. [file 12862_2015_351_MOESM6_ESM.pdf]

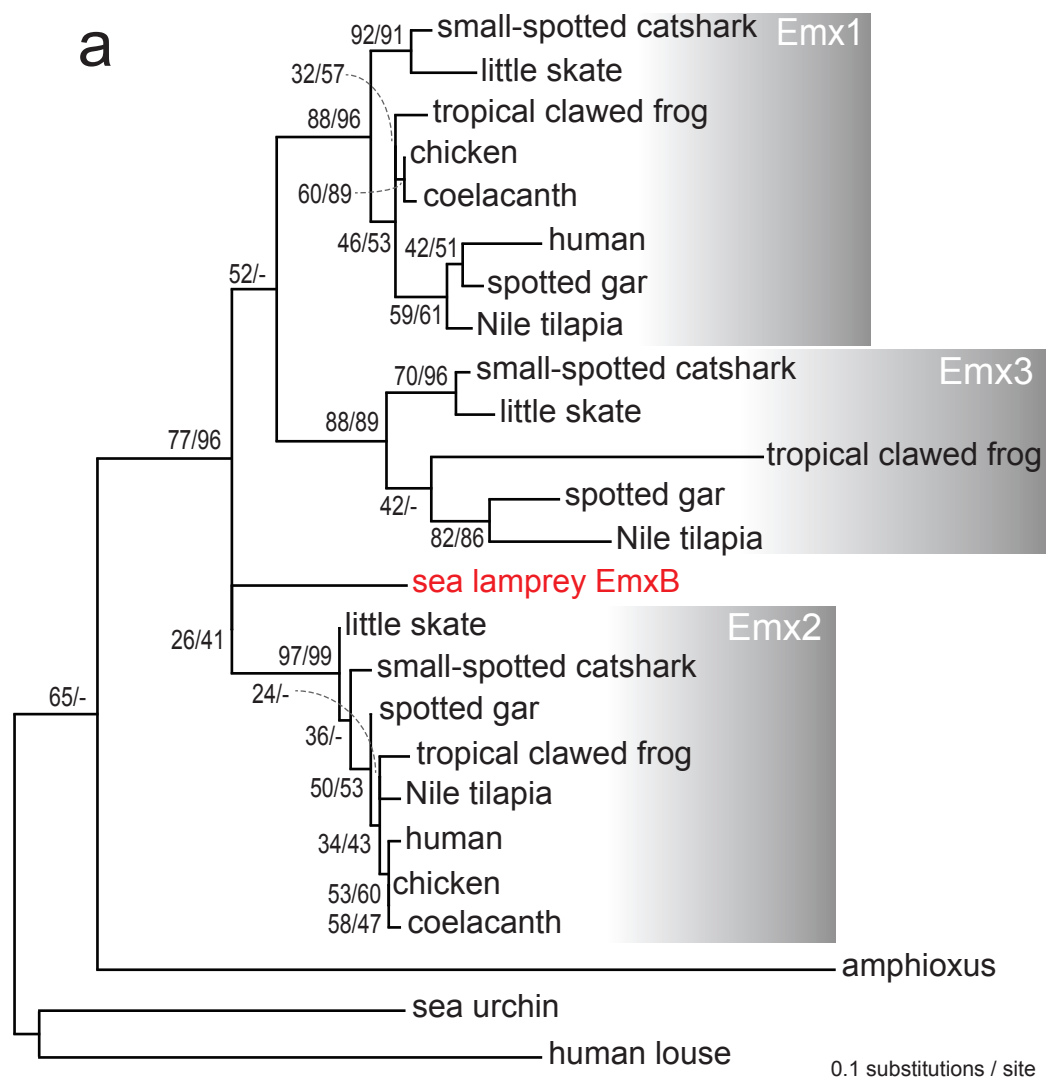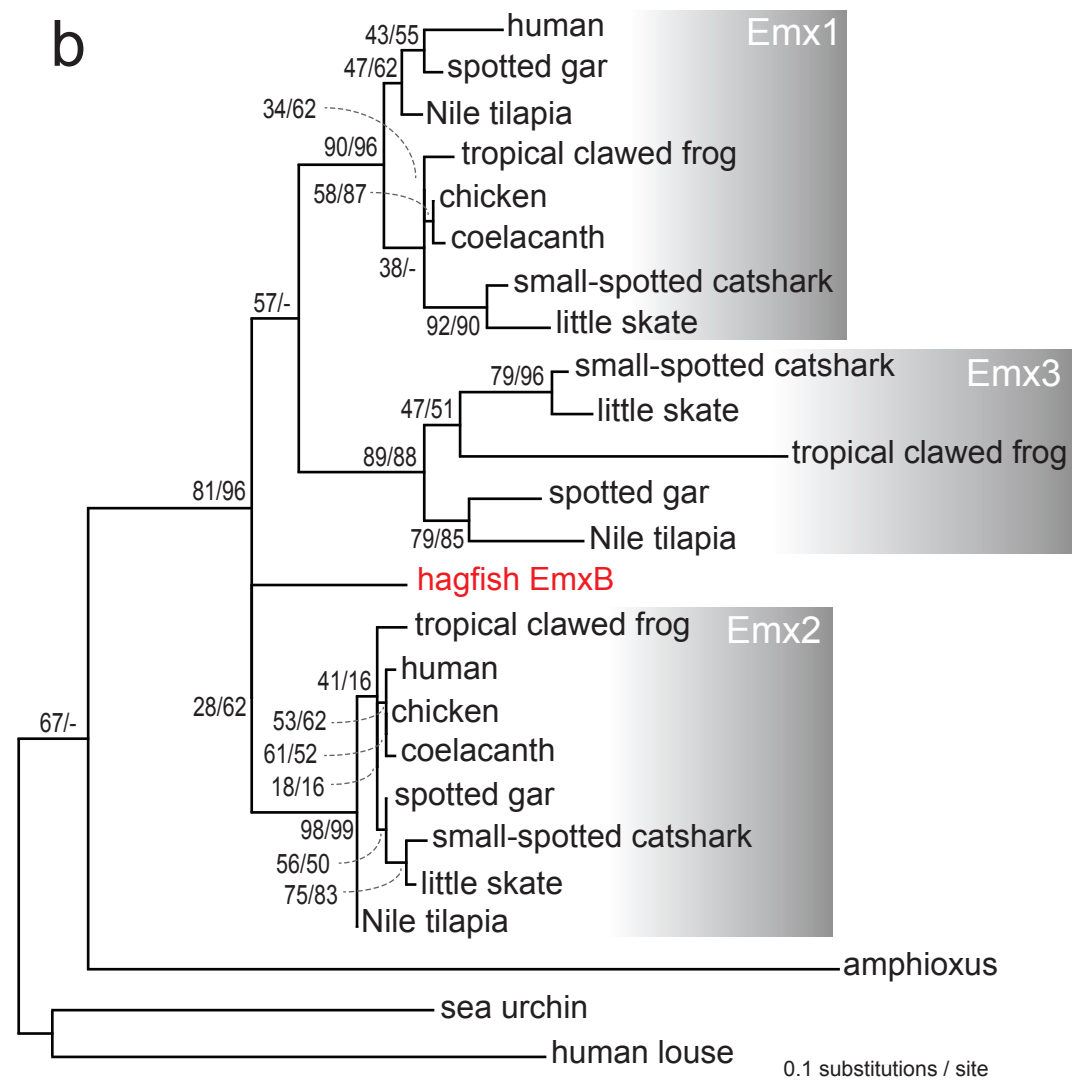

Supplement: Additional file 8: Figure S2. — ML trees for vertebrate Emx genes with only one cyclostome EmxB gene. These ML trees were inferred using 132 amino acid sites in the multiple alignment in Additional file 7: Data S2, assuming JTT+Γ4 model (shape parameter α = 0.65). (a) The ML tree including sea lamprey EmxB as well as the jawed vertebrate data set. (b) The ML tree including hagfish EmxB as well as the jawed vertebrate data set. The numbers at nodes indicate bootstrap probabilities with 100 replicates for the ML and NJ methods, in order. [file 12862_2015_351_MOESM8_ESM.pdf]

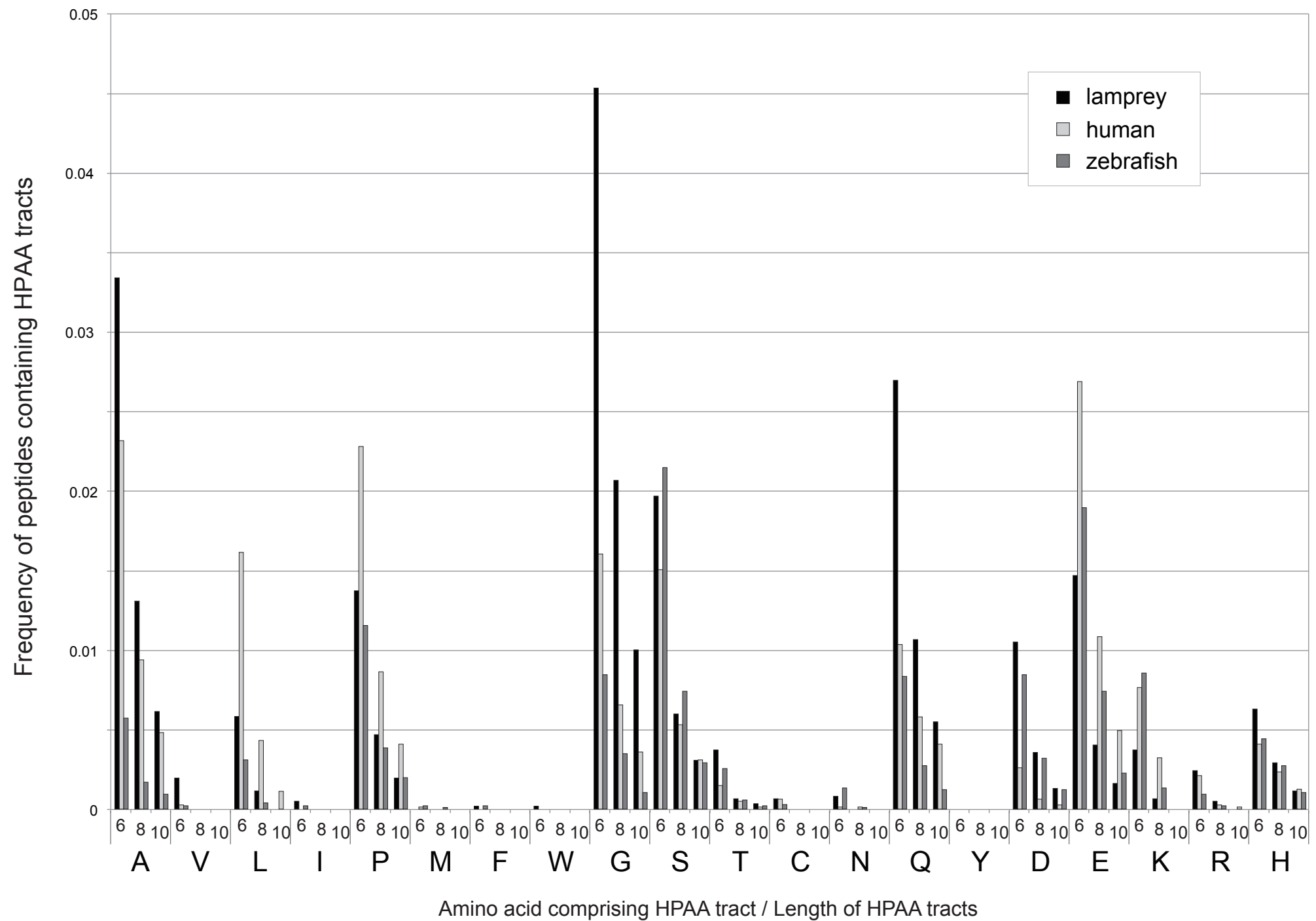

Supplement: Additional file 12: Figure S3. — Genome-wide frequencies of homopolymeric amino acid (HPAA) tracts with variable lengths in the sea lamprey, human, and zebrafish. The peptides with homopolymeric amino acid tracts (those with a stretch of no less than six, eight and ten consecutive residues) were extracted from public database and compared among the three vertebrates (see Methods for details). In the sea lamprey genome, Q and G tract-containing peptides were shown to be more than twice as frequent as in human and zebrafish genomes for all dataset we examined. [file 12862_2015_351_MOESM12_ESM.pdf]
