# Supplementary material for: Reevaluating Emx gene phylogeny: homopolymeric amino acid tracts as a potential factor obscuring orthology signals in cyclostome genes
Source: BMC Evol Biol. 2015 May 4;15:78. doi: 10.1186/s12862-015-0351-z (PMC4464114; doi:10.1186/s12862-015-0351-z)
Supplement: Additional file 7: — Data S2. Multiple sequence alignment of deduced amino acid sequences of Emx genes. The symbol ‘*’ indicates amino acid sites employed in the inference of the phylogenetic trees shown in Figure 3, Additional files 5: Table S2 and 6: Figure S1. The symbol ‘:’ indicates amino acid sites used in the inference of the phylogenetic trees shown in Figure 3, Additional 6: Figure S1b in Additional file 6: Figure S1, and Additional file 6: Figure S1 but not in that in Additional file 6: Figure S1a in Additional file 6: Figure S1. Accession numbers of the sequences included in this alignment are in Additional file 1: Table S1. [file 12862_2015_351_MOESM7_ESM.pdf]

Additional file 7 (Supplementary Data 2)

* indicates amino acid sites used in the inference of the phylogenetic trees shown in Figure S1a ,S1b, and S2 as well as Figure 3.

: indicates amino acid sites used in the inference of the phylogenetic trees shown in Figure 3, S1b, S2 but not in that in Figure S1a.

```
                                     10        20        30        40        50        60        70        80        90       100       110       120
                             =========+=========+=========+=========+=========+=========+=========+=========+=========+=========+=========+=========+
                                                                                          **********
            human louse Emx  --------------------------MPLAPIPMMSVP------------TKPKIGFSIDSIVGGTGSNRIQYSINNKTDRNHDKTIVDNCKIQTSKSSTFPNDEPEDEEDDEEDEIINP
             sea urchin Emx  ----------------------------MSAAELFRPPISGLSSSTTTTTS-KSSCFSIESLVSKDTNHT--------------------------------------------------
             amphioxus Emxb  ------------------------------MMAVLQ----------------PKSSFSIESLVSKDHLAR--------------------------------------------------
           sea lamprey EmxA  ---------------------------------MFQ-P------------P-TKRCFTIESLVAKDGGD---------------------------------------------------
      Japanese lamprey EmxA  ---------------------------------MFQ-P------------P-TKRCFTIESLVAKDGGD---------------------------------------------------
               hagfish EmxA  ---------------------------------MFQ-P------------V-AKRCFTIESLVAKESDPS--------------------------------------------------
           sea lamprey EmxB  ---------------------------------MFQ-P------------ATTKRCFTIESLVAKD------------------------------------------------------
      Japanese lamprey EmxB  ---------------------------------MFQ-P------------ATTKRCFTIESLVAKD------------------------------------------------------
               hagfish EmxB  ---------------------------------MFQ-P------------A-AKRCFTIESLVAKD------------------------------------------------------
                 human Emx1  MCLAGCTPRKAAAPGRGALPRARLPRTAPAAATMFQ-P------------A-AKRGFTIESLVAKDGGTG--------------------------------------------------
               chicken Emx1  ---------------------------------MFQ-P------------S-AKRCFTIESLVAKD------------------------------------------------------
  tropical clawed frog Emx1  ---------------------------------MFQ-P------------A-GKRCFTIESLVAKD------------------------------------------------------
            coelacanth Emx1  ---------------------------------MFQ-P------------T-AKRCFTIESLVAKD------------------------------------------------------
          Nile tilapia Emx1  ---------------------------------MFS-S------------A-GKRCFTIESLVAKE------------------------------------------------------
             zebrafish Emx1  ---------------------------------MFS-A------------T-GKRCFTIESLVAKE------------------------------------------------------
           stickleback Emx1  ---------------------------------MFS-S------------A-GKRCFTIESLVAKE------------------------------------------------------
           spotted gar Emx1  ---------------------------------MFP-P------------A-AKRCFTIESLVAKE------------------------------------------------------
small-spotted catshark Emx1  ---------------------------------MFH-P------------A-SKRCFTIESLVAKDH-----------------------------------------------------
          little skate Emx1  ---------------------------------MFH-A------------G-TKRCFTIESLVAKEN-----------------------------------------------------
                 human Emx2  ---------------------------------MFQ-P------------A-PKRCFTIESLVAKD------------------------------------------------------
               opossum Emx2  ---------------------------------MFQ-P------------A-PKRCFTIESLVAKD------------------------------------------------------
               chicken Emx2  ---------------------------------MFQ-P------------A-PKRCFTIESLVAKD------------------------------------------------------
  tropical clawed frog Emx2  ---------------------------------MFQ-P------------T-PKRCFTIESLVAKD------------------------------------------------------
            coelacanth Emx2  ---------------------------------MFQ-P------------T-PKRCFTIESLVAKD------------------------------------------------------
          Nile tilapia Emx2  ---------------------------------MFQ-P------------T-PKRCFTIESLVAKD------------------------------------------------------
             zebrafish Emx2  ---------------------------------MFQ-P------------T-PKRCFTIESLVAKD------------------------------------------------------
           stickleback Emx2  ---------------------------------MFQ-P------------A-PKRCFTIESLVAKD------------------------------------------------------
           spotted gar Emx2  ---------------------------------MFQ-P------------T-PKRCFTIESLVAKD------------------------------------------------------
small-spotted catshark Emx2  ---------------------------------MFQ-P------------T-PKRCFTIESLVAKD------------------------------------------------------
          little skate Emx2  ---------------------------------MFQ-P------------T-PKRCFTIESLVAKD------------------------------------------------------
               opossum Emx3  ---------------------------------MFT--------------S-ARRSFSIEALVGTPS-----------------------------------------------------
  tropical clawed frog Emx3  ---------------------------------MFL-P------------P-VRKGFSIEALVGPDR-----------------------------------------------------
          Nile tilapia Emx3  ---------------------------------MFQHG------------G-NKKCFTIESLVGKD------------------------------------------------------
             zebrafish Emx3  ---------------------------------MFQ--------------H-NKKCFTIESLVGKD------------------------------------------------------
           stickleback Emx3  ---------------------------------MFHHS------------S-GKKCFSIESLVGKE------------------------------------------------------
           spotted gar Emx3  ---------------------------------MYQ--------------P-SKKCFTIESLVGKD------------------------------------------------------
small-spotted catshark Emx3  ---------------------------------MFQ-P------------A-TKKCFTIEALVGKD------------------------------------------------------
          little skate Emx3  ---------------------------------MFQ-P------------P-AKRCFTIEALVGKD------------------------------------------------------
```

```
                                     130       140       150       160       170       180       190       200       210       220       230       240
                            =========+=========+=========+=========+=========+=========+=========+=========+=========+=========+=========+=========+

            human louse Emx GSPSSDVSNKFESRSPADLRYNGNRTPIDASPTNLIIKNNSAFRSGSPFPTEGKISSYRYPKTASPHRSTDQDISKFESSIHSNSPR-------SSISPASGERTSLSPKAS---LIKPS
             sea urchin Emx -----------------------------------------------------------------------NNHHRH-------HRA----------TP---TP-----------PTSPQ
            amphioxus Emxb -----------------------------------------------------------------------DGPPRTLPHPSQLLEL---------------SR-----------LCRTT
          sea lamprey EmxA ------------------------------------------------------------------------------------SVS-------AAATL-QQQQQQQQQQQQQQQQQQQP
    Japanese lamprey EmxA ------------------------------------------------------------------------------------SVS-------AAATLQQQQQQQQQQQQQQQQQQQQP
              hagfish EmxA ----------------------------------------------------------------------------------------------------------------------PN
          sea lamprey EmxB ------------------------------------------------------------------------------------CPA-------PTSRSE--QQQQQQQQQQHEAPLRPA
    Japanese lamprey EmxB ------------------------------------------------------------------------------------CPP-------PPSRSEQQQQQQQQQQQQHEAPLRPA
              hagfish EmxB ------------------------------------------------------------------------------------CPA-------PGGASNRPDE-----------PLRPA
                human Emx1 -----------------------------------------------------------------------GGTGGGGAGSHLLAAA----------AS---EE-----------PLRPT
              chicken Emx1 ------------------------------------------------------------------------------------TPL----------SS---EE-----------PLRPS
  tropical clawed frog Emx1 ------------------------------------------------------------------------------------NPL----------SS---EE-----------PLRPA
           coelacanth Emx1 ------------------------------------------------------------------------------------SPL----------TA---EE-----------PIRPT
         Nile tilapia Emx1 ------------------------------------------------------------------------------------NPL----------TA---ED-----------PIRPT
            zebrafish Emx1 ------------------------------------------------------------------------------------SPI----------TL---ED-----------PIRPT
          stickleback Emx1 ------------------------------------------------------------------------------------NPL----------IP---ED-----------PIRPT
          spotted gar Emx1 ------------------------------------------------------------------------------------SPL----------TA---EE-----------PIRPT
small-spotted catshark Emx1 ------------------------------------------------------------------------------------NPL----------SP---EE-----------PLRPA
         little skate Emx1 ------------------------------------------------------------------------------------SPL----------SP---DE-----------SLRPA
                human Emx2 ------------------------------------------------------------------------------------SPL-------PASRS---ED-----------PIRPA
              opossum Emx2 ------------------------------------------------------------------------------------NPL-------PASRS---ED-----------PIRPA
              chicken Emx2 ------------------------------------------------------------------------------------SPL-------PASRS---ED-----------PIRPA
  tropical clawed frog Emx2 ------------------------------------------------------------------------------------SPL-------PVSRS---EE-----------PIRPA
           coelacanth Emx2 ------------------------------------------------------------------------------------SPL-------PVSRS---ED-----------PIRPS
         Nile tilapia Emx2 ------------------------------------------------------------------------------------NPV-------PASRT---EE-----------PIRPA
            zebrafish Emx2 ------------------------------------------------------------------------------------NPL-------PSSRS---EE-----------PIRPA
          stickleback Emx2 ------------------------------------------------------------------------------------NHV-------PASRT---EE-----------PIRPA
          spotted gar Emx2 ------------------------------------------------------------------------------------NPL-------PASRS---EE-----------PIRPA
small-spotted catshark Emx2 ------------------------------------------------------------------------------------SPL-------PASRS---EE-----------PIRPA
         little skate Emx2 ------------------------------------------------------------------------------------SPL-------PASRS---EE-----------PIRPA
              opossum Emx3 ------------------------------------------------------------------------------------EPL----------VR---SA-----------PARPC
  tropical clawed frog Emx3 -------------------------------------------------------------------------------------------------TP---EE-----------PLRPT
         Nile tilapia Emx3 ------------------------------------------------------------------------------------GSS------HGGGSG---EE-----------PIRPT
            zebrafish Emx3 ------------------------------------------------------------------------------------SNS-------SNAAA---DE-----------PIRPT
          stickleback Emx3 ------------------------------------------------------------------------------------ASSRGPGSTGGAGSG---EE-----------PIRPT
          spotted gar Emx3 ------------------------------------------------------------------------------------ANS-------S--AG---DE-----------PVRPT
small-spotted catshark Emx3 ------------------------------------------------------------------------------------SPS-------T--LG---DE-----------PLRPT
         little skate Emx3 ------------------------------------------------------------------------------------NAS-------T--LG---DE-----------PLRPT
```

```
                                    250       260       270       280       290       300       310       320       330       340       350       360
                            =========+=========+=========+=========+=========+=========+=========+=========+=========+=========+=========+=========+
                                                                                                  *****
          human louse Emx       VLTPANV--------------------------------VPFGSEM-----------------------NAAA-AL-KGIYLPTDPVMHGVGHPSHHH-H---P-------HPLALAAAA
           sea urchin Emx       AAATASTMES-----------------------------NSLASSPFTPPATSRHGGIIKPMPTEASFFNTLS-GM-KGLYHP-EPIYHT-AAAAGP------PSHVGPIQHQLG-----
       amphioxus Emxb           ATTTIN-------------------------------------SGIPHVPYTQG-G-----IPVSLPSLGSLC-GGPRPVSVP-HYVTY--SAPTMVP-----PGLLSGAPPTV------
         sea lamprey EmxA       AYPIPGGANPLTCGA---------------------QPPHPFAAAAAAAAAAAA-S-------------RAGM--H-PELFFH-DAHH---LQPLLAV-----PAMHPSG-HHHH-----
   Japanese lamprey EmxA       AYPIPGGANPLTCGA---------------------QPPHPFAAAAAAAAAAAA-S-------------RAGM--H-PELFFH-DAHH---LQPLLAV-----PAMHPSGHHHHH-----
             hagfish EmxA       LLHVSYGGS------------------------------HSGIGSVHSGPHYGP-G-------------RPGYGPHQGDLFFPMEGVH-P-G-PVVPV-----PSM---ASHPMT-----
         sea lamprey EmxB       ALSFAGTHNHQHHQHQQHHHHSSASVSAAAAAAAAAAAAAAFVPAFPQP----T-A-------------RALY-PHPAELLYS-DPGHHP-GSAGGPLQV---PA------LP-P-----
   Japanese lamprey EmxB       ALSFAGSHNHQHHQHQQHHHHSSASVSAAAAAAAAAAAAAAFVPAFPQP----T-A-------------RALY-PHPAELLYS-DPGHHP-GSAGGPLQV---PA------LP-P-----
             hagfish EmxB       ALSFAGAPVH-------------------------SQPGTAFMPGFAQP----A-G-------------RTLY-PH-ADLMYP-EPGPH--HAASGPLPI---PA------LPVG-----
              human Emx1       ALNYPHPS----------------------------AAEAAFVSGFPAAAAAGA-G-------------RSLY-GG-PELVFP-EAMNH----PALTV-H---PA------HQLG-----
            chicken Emx1       ALNYPPP-----------------------------AASDAFPGGFQGAA---A-G-------------RALY-GG-TELVFP-EAVGH----PALPV-A---P-------HQLG-----
 tropical clawed frog Emx1       ALPYPGA------------------------------PAEAFVSGFPS-P---A-G-------------RSLY-NN-PELVFP-ETVSH----PPLTV-H---P-------HQLG-----
          coelacanth Emx1       AFSYPN-------------------------------PAEAFVNGFQT-T---A-G-------------RSLY-TS-PELMFP-EAVSH----PSLTV-H---P-------HQLG-----
        Nile tilapia Emx1       ALSYSNP------------------------------TTDALMNSYQAPP---P-A-------------RSLY-QS-PDLVFP-ETMNH----PSLTV-A---P-------HQLG-----
           zebrafish Emx1       ALSYSA-------------------------------PADSFLNGYQS-P---A-G-------------RALY-PN-PELVFS-ETVNH----APLSM-H---P-------HQLG-----
         stickleback Emx1       ALSYSNP------------------------------TTDALMNSYQN-P---P-A-------------RTLY-QS-PDLVFP-E--NH----PSLTV-A---P-------HQLG-----
         spotted gar Emx1       ALSYSN-------------------------------PTDAFMTGFQG-Q---A-G-------------RSLY-SG-PELVFP-ETVNH----PSLTV-H---P-------HQLG-----
small-spotted catshark Emx1       AVTYPP-------------------------------STDAFAGGLQA-----A-A-------------RSLY-PG-PELMFQ-EAVSH----PSLPV-H---P-------HQLG-----
        little skate Emx1       ALSYPT-------------------------------SADGFPNAFQA-----P-G-------------RSLY-SG-PDLMFQ-EAVAH----PALPVHH---P-------HPLS-----
              human Emx2       ALSYANS-----------------------------SPINPFLNGFHSAAAAAA-G-------------RGVY-SN-PDLVFA-EAVSHP-PNPAVPV-HPVPPP------HALA-----
            opossum Emx2       ALSYANS-----------------------------SPINPFLNGFHT----AA-G-------------RGVY-SN-PDLVFA-EAVSHP-PNPAVPV-HPVPPP------HALA-----
            chicken Emx2       ALSYANS-----------------------------SPMNPFLNGFHS-----T-G-------------RGVY-SN-PDLVFA-EAVSHP-PNPAVPV-HPVPPP------HALA-----
 tropical clawed frog Emx2       ALSYANS-----------------------------APMNPFLNGFHP-----T-G-------------RGVY-SN-PDLVFA-EAVSHP-PNPAVPV-HPVPPP------HALA-----
          coelacanth Emx2       ALSYANS-----------------------------APMNPFLNGFHS-----S-S-------------RSVY-NN-PDLLFA-EAVSHP-PNAAVPV-HPVPPP------HALA-----
        Nile tilapia Emx2       ALSYANS-----------------------------GQMNPFLNGFHS-----G-G-------------RGVY-SN-PDLVFA-EAVSHP-PNSTVPV-HPVAPP------HALA-----
           zebrafish Emx2       ALSYANS-----------------------------SQMNPFLNGFHS-----S-G-------------RGVY-SN-PDLVFA-EAVSHP-PNSAVPV-HSVPPP------HALA-----
         stickleback Emx2       ALSYANS-----------------------------GQMNPFLNGFHS-----G-G-------------RGVY-SN-PDLVFA-EAVSHQ-QNSAVPV-HSVAPP------HALA-----
         spotted gar Emx2       ALSYANS-----------------------------TQMNPFLNGFHS-----S-G-------------RGVY-SN-PDLVFA-EAVSHP-PNSAVPV-HPVPPP------HALA-----
small-spotted catshark Emx2       ALSYANP-----------------------------SSVNPFLNGFHT-----G-G-------------RALY-SN-PDLVFA-DAVAHQ-TSPAVPV-HPV-PP------HALA-----
        little skate Emx2       ALSYANS-----------------------------SPVNPFLNGFHT-----S-G-------------RAIY--N-PELVFA-DAVSHQ-TNTAVPV-HPV-PP------HALA-----
            opossum Emx3       ASATWT-----------------------------------------------A-A-------------RLLV-PE-PPVVPD-RGASGR-LGPA--------P----------------
 tropical clawed frog Emx3       ALKYPEPG-------------------------------HGLTPTPLR-----P-G-------------VRLL-GA-PDLFFP-EPAAPP-YGPSLGL-----PQ------HRI------
        Nile tilapia Emx3       ALRFPDSLHP-------------------------AAAAGVFGSCFQG-----SNG-------------RTLF----PDMVLQ-EPGTHAQGPGGLPL-H---P-------LQI------
           zebrafish Emx3       ALRFTESIHP-----------------------------SPFGSCFQN-----S-G-------------RTLYSSS-PEMMFT-DPATHS-TNSGLSL-R---H-------LQI------
         stickleback Emx3       ALRFSESLHP--------------------------AASGVFGSCFQG-----SGG-------------RTLY-STGPDVVLQ-EPGAH--GHGGLPL-H---P-------LQL------
         spotted gar Emx3       ALRFTESVHP-----------------------------TPFGSCFQN-----T-G-------------RTLY-ST-PDLMFS-EPGTHA-ASSALSL-H---P-------LQL------
small-spotted catshark Emx3       ALRYADSVHA-----------------------------SPFGSGFQN-----A-G-------------RTLY-GS-PELLFP-EPVAHA-ASPALSM-HPGNP-------HHL------
        little skate Emx3       ALRYADSLHA-----------------------------SHFGSGFQN-----A-G-------------RTLY-SS-PELLFP-DPVTHA-ASPALSI-HPGNA-------HHL------
```

```
                                    370       380       390       400       410       420       430       440       450       460       470       480
                           =========+=========+=========+=========+=========+=========+=========+=========+=========+=========+=========+=========+
                                                                     ********:** ** ***  ********:******       ****************************
          human louse Emx  QHFQAAGLAAAIAQHQQQPGGFLSSGPPSHLPPGHPGHH-----GPQIPPRDTYPLYPWLLSRHGRIF--SHRFPGGPDIPGFLL---QPF-RKPKRIRTAFSPSQLLKLEHAFEKNHYV
          sea urchin Emx   ------------LAHHHNMAAAAAAAAAQHNHPALAGVFGAHGLGGP--RRDGFGLYPWLLARN-RFLAAGHRPFPGDLPNGGFF-LQHPF-RKPKRIRTAFSPSQLLRLENAFEKNHYV
         amphioxus Emxb    --------------------------------PH-QSPV-----SPP--YQTHLPYNPWMLGGH-PPY--GHRLQGPDMGNGSLIHLQNPF-RKPKRIRTAFTPSQLLRLEHAFEKNHYV
       sea lamprey EmxA    -----------------------HH--LSHPPPSLPLFG-----GPQ--GRDAISLYPWLLHRP-RYL--GHRYPGADGNAESLL-LHSPFARKPKRIRTAFSPSQLLRLEHAFEKNHYV
  Japanese lamprey EmxA    -----------------------HH--LSHPPPSLPLFG-----GPQ--GRDAISLYPWLLHRP-RYL--GHRYPGADGSAESLL-LHSPFARKPKRIRTAFSPSQLLRLEHAFEKNHYV
           hagfish EmxA    -----------------------GQ--SLHPAPSIPLLG-----APH--SRDHFSLYPWLFHRP-PYL--GHRFRGADGGPETLL-LHGPFARKPKRIRTAFSPSQLLRLEHAFEKNHYV
       sea lamprey EmxB    -----------------------HH-HLQ---HS-HPLF-----GPP--QRDPMTFYPWLLNRH-RYL--GHRYPGPETGHEGLL-FPGPLARKPKRIRTAFSPSQLLRLEHAFEKNHYV
  Japanese lamprey EmxB    -----------------------HH-HLQ---HS-HPLF-----GPP--QRDPMTFYPWLLNRH-RYL--GHRYPGPETGHEGLL-FPGPFARKPKRIRTAFSPSQLLRLEHAFEKNHYV
           hagfish EmxB    -----------------------AH-PLQ---PSHHPLF-----GPP--QRDPMTFYPWLLNRH-RYL--SHRYPGPDSCAESLL-FPGPFARKPKRIRTAFSPSQLLRLEHAFEKNHYV
             human Emx1    -----------------------AS-PLQ---PP-HSFF-----GAQ--HRDPLHFYPWVL-RN-RFF--GHRFQASDVPQDGLL-LHGPFARKPKRIRTAFSPSQLLRLERAFEKNHYV
           chicken Emx1    -----------------------AS-PLP---HP-HSFF-----GPQ--HRDPLNFYPWVL-RN-RFF--GHRFQGSEVSQESLL-LHGPFARKPKRIRTAFSPSQLLRLERAFEKNHYV
tropical clawed frog Emx1  -----------------------AS-HLQ---HP-HSFF-----APQ--HRDPLNFYPWVL-RN-RFF--GHRFQGGDVSQESLL-LHGPFARKPKRIRTAFSPSQLLRLERAFEKNHYV
        coelacanth Emx1    -----------------------TP-HLQ---HP-HSFF-----GPQ--HRDPLNFYPWVL-RN-RFF--GHRFQGSEVSQESLL-LHGPFARKPKRIRTAFSPSQLLRLERAFEKNHYV
      Nile tilapia Emx1    -----------------------GS-HLQ---HP-H-FF-----GTQ--HRDPLNFYPWVL-RN-RFF--GHRFQGNDVSQDSLL-LHGPFARKPKRIRTAFSPSQLLRLERAFEKNHYV
         zebrafish Emx1    -----------------------SA-PLQ---HP-H-FF-----GTQ--HREPLNFYPWVL-RN-RFF--GHRFQGNDVSQDTLL-LHGPFARKPKRIRTAFSPSQLLRLERAFEKNHYV
       stickleback Emx1    -----------------------GSHHLQ---HP-H-FF-----GTQ--HRDPLNFYPWVL-RN-RFF--GHRFQGNEVPQDSLL-LHGPFARKPKRIRTAFSPSQLLRLERAFEKNHYV
       spotted gar Emx1    -----------------------SS-HLQ---HP-HSFF-----GTQ--HRDPINFYPWVL-RN-RFF--GHRFQGNDVSQDGLL-LHGPFARKPKRIRTAFSPSQLLRLERAFEKNHYV
small-spotted catshark Emx1-----------------------TH-HLR---SH-HTFF-----APQ--HRDPLNFYPWVL-RN-RFF--GHRFQGSDAAHESML-LHGPFARKPKRIRTAFSPSQLLRLERAFEKNHYV
      little skate Emx1    -----------------------SH-HLQ---TP-HPFF-----SPQ--HRDPLNFYPWVL-RN-RLF--GHRFQGPDGAQETML-LHGPFARKPKRIRTAFSPSQLLRLERAFEKNHYV
             human Emx2    -----------------------AH-PLPSSHSP-HPLF-----ASQ--QRDPSTFYPWLIHRY-RYL--GHRFQGNDTSPESFL-LHNALARKPKRIRTAFSPSQLLRLEHAFEKNHYV
           opossum Emx2    -----------------------AH-PLPSSHSP-HPLF-----ASQ--QRDPSTFYPWLIHRY-RYL--GHRFQGNETSPESFL-LHNALARKPKRIRTAFSPSQLLRLEHAFEKNHYV
           chicken Emx2    -----------------------AH-PLPASHST-HPLF-----ASQ--QRDPSTFYPWLIHRY-RYL--GHRFQGNETSPESFL-LHNALARKPKRIRTAFSPSQLLRLEHAFEKNHYV
tropical clawed frog Emx2  -----------------------AH-PLPTSHSP-HPLF-----ASQ--QRDPSTFYPWLIHRY-RYL--GHRFQGNDTSAESFL-LHNALARKPKRIRTAFSPSQLLRLEHAFEKNHYV
        coelacanth Emx2    -----------------------AH-PLPSSHTP-HPLF-----ASQ--QRDPSTFYPWLIHRY-RYL--GHRFQGNETSPESFL-LHNALARKPKRIRTAFSPSQLLRLEHAFEKNHYV
      Nile tilapia Emx2    -----------------------AH-PLSSSHSP-HPLF-----ASQ--QRDPSTFYPWLLHRY-RYL--GHRFQGNETSPESFL-LHNALARKPKRIRTAFSPSQLLRLEHAFEKNHYV
         zebrafish Emx2    -----------------------AH-PLSSSHSP-HPLF-----ASQ--QRDPSTFYPWLIHRY-RYL--GHRFQGNETSPESFL-LHNALARKPKRIRTAFSPSQLLRLEHAFEKNHYV
       stickleback Emx2    -----------------------AH-PLSSSHSP-HPLF-----ASQ--QRDPSTFYPWLLHRY-RYL--GHRFQGNETSPESFL-LHNALARKPKRIRTAFSPSQLLRLEHAFEKNHYV
       spotted gar Emx2    -----------------------AH-PLSSSHSP-HPLF-----ASQ--QRDPSTFYPWLIHRY-RYL--GHRFQGNETSPESFL-LHNALARKPKRIRTAFSPSQLLRLEHAFEKNHYV
small-spotted catshark Emx2-----------------------GH-PLQSSHSP-HPLF-----TSQ--QRDASSFYPWLIHRY-RYL--GHRFQGNEASPESFL-LHNALARKPKRIRTAFSPSQLLRLEHAFEKNHYV
      little skate Emx2    -----------------------AH-PLQSSHSP-HPLF-----ASQ--QRDPSSFYPWLIHRY-RYL--GHRFQGNETSPESFL-LHNALARKPKRIRTAFSPSQLLRLEHAFEKNHYV
           opossum Emx3    ---------------------------------------------PG--PRDPCSCCPWLL-RS-RLW--TPSFSEIH-SAENLM-LCGPFTRKPKRIRTAFSPSQLLRLERAFEKNHYV
tropical clawed frog Emx3  ----------------------------------------------L--HRDPLSFCPWIL-RH-RLP--DSFFLGEENTPDNYL-LHGPFSRKPKRIRTAFSPSQLLRLEGAFEKNHYV
      Nile tilapia Emx3    --------------------------------PP-QSFF-----SPQ--HRDALSFYPWVL-RS-RYL--GHRFQGEDSSPENLL-LHGPFSRKPKRIRTAFSPSQLLRLERAFEKNHYV
         zebrafish Emx3    --------------------------------PT-QPFF-----SPH--QRDTLNFYPWVL-RN-RYL--GHRFQGDDSSPENLL-LHGPFSRKPKRIRTAFSPSQLLRLERAFEKNHYV
       stickleback Emx3    --------------------------------PP-QGFF-----SPQ--HREALGFYR-VL-RS-RYL--GHRFQGEDSSPENLL-LHGPFSRKPKRIRTAFSPSQLLRLERAFEKNHYV
       spotted gar Emx3    --------------------------------PA-QPFF-----GPH--QRETLNFYPWVL-RN-RYL--GHRFQGEDSSPENLL-LHGPFSRKPKRIRTAFSPSQLLRLERAFEKNHYV
small-spotted catshark Emx3--------------------------------PA-QSFF-----APQ--QREALSFYPWVL-RN-RYL--GHRFQGSEGGAEGLL-LHGPFTRKPKRIRTAFSPSQLLRLERAFEKNHYV
      little skate Emx3    --------------------------------SA-QSFF-----APQ--QREALSFYPWVL-RN-RYL--GHRFQGGEGGAESLL-LHGPFTRKPKRIRTAFSPSQLLRLERAFEKNHYV
```

```
                                       490       500       510       520       530       540       550       560       570       580
                                 =========+=========+=========+=========+=========+=========+=========+=========+=========+=========+
                                 ***************************************          *****************   **
           human louse Emx       VGAERKQLAQSLSLTETQVKVWFQNRRTKHKRMQQEEEAKAQQQSSSTGSSKNSHHVNKWKVETQN---SSQS---TTASDGTQYIEYDEDEIGSSGEEA
            sea urchin Emx       VGAERKQLAASLNLTETQVKVWFQNRRTKYKRIKSEEEG-EEEP-----KKKGSHHVNRWMETQQ---SIEER----ERD--------------------
           amphioxus Emxb        VGQERKHLAQSLSLTETQVKVWFQNRRTKHKREQQGDDGRDSPT-----KHRGAHHVSRWRQATQQ---LTTTVEHTSAPDGHDETKSTSSCSDAGSR--
         sea lamprey EmxA        VGAERKQLASSLSLSETQVKVWFQNRRTKYKRQKLEEEGPESPQ-----KKKSSHHINRWRQATNQ---GSGDEIDVTSDD-------------------
    Japanese lamprey EmxA        VGAERKQLASSLSLSETQVKVWFQNRRTKYKRQKLEEEGPESPQ-----KKKSSHHINRWRQATNQ---GSGDEIDVTSDD-------------------
             hagfish EmxA        VGAERKQLAGSLTLTETQVKVWFQNRRTKYKRQKMEEEGPESPH-----KKKGSHHINRWRLATNQ---SSGDEIDVTSDD-------------------
         sea lamprey EmxB        VGSERKQLASSLSLSETQVKVWFQNRRTKHKRQKLEEEGPDEQQ-----KKKGTHHVNRWRMATKQ---PSSEDIDVTSDD-------------------
    Japanese lamprey EmxB        VGSERKQLASSLSLTETQVKVWFQNRRTKHKRQKLEEEGPDEQQ-----KKKGTHHVNRWRMATKQ---PSSEDIDVTSDD-------------------
             hagfish EmxB        VGSERKQLASSLSLTETQVKVWFQNRRTKHKRQKLEEEGPDEHQ-----KKKGTHHINRWRMATKQ---TSSEDIDVTSEN-------------------
               human Emx1        VGAERKQLAGSLSLSETQVKVWFQNRRTKYKRQKLEEEGPESEQ-----KKKGSHHINRWRIATKQ---ANGEDIDVTSND-------------------
             chicken Emx1        VGAERKQLASSLSLSETQVKVWFQNRRTKYKRQKLEEEGPDSEQ-----KKKGSHHINRWRLATKQ---SSGEDIDVTSND-------------------
 tropical clawed frog Emx1        VGAERKQLASSLSLSETQVKVWFQNRRTKYKRQKLEEEGPDSDQ-----KKKGSHHINRWRLATKQ---PNGEDIDVTSND-------------------
         coelacanth Emx1        VGAERKQLASSLSLSETQVKVWFQNRRTKYKRQKLEEEGSESDQ-----KKKGSHHINRWRLATKQ---SSPEDIDVTSND-------------------
        Nile tilapia Emx1        VGAERKQLANSLSLSETQVKVWFQNRRTKYKRQKLEEEGPDSQQ-----KKKGNHHINRWRIATKQ---TSSEDIDVTSED-------------------
           zebrafish Emx1        VGAERKQLANSLSLSETQVKVWFQNRRTKYKRQKLEEEGPECTQ-----KKKGNHHINRWRIATKQ---TGSEDIDVMSDA-------------------
         stickleback Emx1        VGAERKQLANGLSLSETQVKVWFQNRRTKYKRQKLEEEGPDSQQ-----KKKGNHHINRWRMATKQ---PGSEDIDVTSED-------------------
         spotted gar Emx1        VGAERKQLANSLSLSETQVKVWFQNRRTKYKRQKLEEEGPECQQ-----KKKGTHHINRWRIATKQ---ASSEDIDVTSDD-------------------
small-spotted catshark Emx1        VGAERKQLASSLSLSETQVKVWFQNRRTKYKRQKLKEEGPDSQQ-----KKKGSHHVNRWRLATKQ---SSPEAIDVTSED-------------------
        little skate Emx1        VGAERKQLAGSLSLSETQVKVWFQNRRTKYKRQKLEEEGPDSQQ-----KKKGSHHVNRWRLATKQ---SSPEDIDVISED-------------------
               human Emx2        VGAERKQLAHSLSLTETQVKVWFQNRRTKFKRQKLEEEGSDSQQ-----KKKGTHHINRWRIATKQ---ASPEEIDVTSDD-------------------
             opossum Emx2        VGAERKQLAHSLSLTETQVKVWFQNRRTKFKRQKLEEEGSDSQQ-----KKKGTHHINRWRIATKQ---ASPEEIDVTSDD-------------------
             chicken Emx2        VGAERKQLAHSLSLTETQVKVWFQNRRTKFKRQKLEEEGSDSQQ-----KKKGTHHINRWRIATKQ---ASPEEIDVTSDD-------------------
 tropical clawed frog Emx2        VGAERKQLAHSLSLTETQVKVWFQNRRTKFKRQKLEEEGSDSSQ-----KKKGAHHINRWRLATKQ---ASPEEIDVTSDD-------------------
          coelacanth Emx2        VGAERKQLAHSLSLTETQVKVWFQNRRTKFKRQKLEEEGTDSQS-----KKKGTHHINRWRIATKQ---ASPEEIDVTSDD-------------------
        Nile tilapia Emx2        VGAERKQLAHSLSLTETQVKVWFQNRRTKFKRQKLEEEGSESQQ-----KKKGSHHINRWRLATKQ---ASPEEIDVTSDD-------------------
           zebrafish Emx2        VGAERKQLAHSLSLTETQVKVWFQNRRTKFKRQKLEEEGSDSQQ-----KKKGTHHINRWRLATKQ---GSPEEIDVTSDD-------------------
         stickleback Emx2        VGAERKQLAHSLSLTETQVKVWFQNRRTKFKRQKLEEEGSESQQ-----KKKGSHHINRWRMATKQ---GSPEEIDVTSDD-------------------
         spotted gar Emx2        VGAERKQLAHSLSLTETQVKVWFQNRRTKFKRQKLEEEGSDSQQ-----KKKGTHHINRWRLATKQ---SSPEEIDVTSDD-------------------
small-spotted catshark Emx2        VGAERKQLAHSLSLTETQVKVWFQNRRTKFKRQKLEEEGTDAQQ-----KKKGTHHVNRWRLATKQ---SSPEEIDVTSDD-------------------
        little skate Emx2        VGAERKQLAHSLSLTETQVKVWFQNRRTKFKRQKLEEEGTDAQQ-----KKKGTHHVNRWRLATKQ---SSPEEIDVTSDD-------------------
             opossum Emx3        VGAERKQLANSLCLTETQVKVWFQNRRTKHKRQKMDEECPTAEE-----KRKPGQLKSQWQVAARQ---GTDEDIDVTSED-------------------
 tropical clawed frog Emx3        VGAERKQLANSLCLTETQVKVWFQNRRTKHKRQKLEEECPESQQ-----KRKSTQHVSRWRMATCQ---TSPKDIDVTSDD-------------------
        Nile tilapia Emx3        VGAERKQLASGLCLTETQVKVWFQNRRTKHKRQKLEEESPEAQQ-----KRKGSQHVSRWRAATQQ---AGGEDVDVISED-------------------
           zebrafish Emx3        VGAERKQLANGLCLTETQVKVWFQNRRTKHKRQKLEEESPDPQQ-----KRKGSQHVSRWRVATQQ---GSPEDIDVISED-------------------
         stickleback Emx3        VGAERKQLASALCLTETQVKVWFQNRRTKHKRQKLEEESPEAQQ-----KRKGSQHINRWRAATQQPGGGGGEDVDVLSED-------------------
         spotted gar Emx3        VGAERKQLANGLCLTETQVKVWFQNRRTKHKRQKLEEEAPESQQ-----KRKGNQHVNRWRVATRQ---GSPEDIDVISED-------------------
small-spotted catshark Emx3        VGAERKQLANSLCLTETQVKVWFQNRRTKYKRQKLEEESPDTQQ-----KRKGTQHVNRWRMATRQ---TSPEDIDVISED-------------------
        little skate Emx3        VGAERKQLASSLCLTETQVKVWFQNRRTKYKRQKLEEESPDTQQ-----KRKGTQHVSRWRMATRQ---TSPEDIDVISED-------------------
```
