# Supplementary material for: Reevaluating Emx gene phylogeny: homopolymeric amino acid tracts as a potential factor obscuring orthology signals in cyclostome genes
Source: BMC Evol Biol. 2015 May 4;15:78. doi: 10.1186/s12862-015-0351-z (PMC4464114; doi:10.1186/s12862-015-0351-z)
Supplement: Additional file 9: Table S3. — Location of genes in the Emx2/EmxB-containing synteny. This table includes chromosomal locations (or locations on genomic scaffolds) and base positions of the genes in Figure 4a. [file 12862_2015_351_MOESM9_ESM.pdf]

Additional file 9 (Table S3). Location of genes in the *Emx2*/*EmxB*-containing synteny

Species	Human	Chicken	Coelacanth	Spotted gar	Sea lamprey
Chromosome or scaffold	Ch. 10	Ch.6	JH126834.1	LG5	GL476962
Slc18a2	ENSG00000165646 (119000604-119038941)	ENSGALG00000009289 (28676708-28694550)	ENSLACG00000013037 (800748-855817)	ENSLOCG00000009666 (19377666-19403643)	Manually curated, partial (1-38497)
Pdzd8	ENSG00000165650 (119040000-119134978)	ENSGALG00000029095 (28696248-28747446)	ENSLACG00000013505 (867403-870159)	ENSLOCG00000009680 (19408954-19468897)	ENSPMAG00000006427 (41039-105529)
Emx2 or EmxB	ENSG00000170370 (119301955-119309056)	ENSGALG00000009302 (28814903-28820582)	ENSLACG00000014520 (1044239-1050042)	ENSLOCG00000009698 (19494583-19499269)	ENSPMAG00000006507 (985773-1154765)
Rab11fip2	ENSG00000107560 (119764427-119806114)	ENSGALG00000009304 (29003777-29047647)	ENSLACG00000015968 (1414165-1459974)	ENSLOCG00000009709 (19645245-19685141)	ENSPMAG00000006463 (466060-478524)

The numbers in parentheses indicate ranges of the individual genes in nucleotide positions.
