# Supplementary material for: Reevaluating Emx gene phylogeny: homopolymeric amino acid tracts as a potential factor obscuring orthology signals in cyclostome genes
Source: BMC Evol Biol. 2015 May 4;15:78. doi: 10.1186/s12862-015-0351-z (PMC4464114; doi:10.1186/s12862-015-0351-z)
Supplement: Additional file 10: — Data S4. Multiple sequence alignment of deduced amino acid sequences of Pdzd8 genes. The symbol ‘*’ indicates amino acid sites employed in the inference of the phylogenetic trees shown in Figure 4b. Accession numbers of the sequences included in this alignment are in Additional file 10: Data S4. [file 12862_2015_351_MOESM10_ESM.pdf]

Additional file 10 (Supplementary Data 4)

* indicates amino acid sites used in the inference of the phylogenetic trees shown in Figure 3b.

```
                                 10        20        30        40        50        60        70        80        90       100       110       120
                         =========+=========+=========+=========+=========+=========+=========+=========+=========+=========+=========+=========+
                                *************************   ***
     polychaete worm     --M----IAIILCSIFVGIALTLIAQVLFLYRWFFSLP----VEGPPWKPQSEPYCLPKELLDAI------------------------------------------------------R
         human louse     --MFISWILTVLCSICLGVLITLIFQYYVYIKYFQQQG----ATIPEKKIKLEPFQLPKVLLDVI-------------------------------------------------------
          sea urchin     --M----FFIILLSILFGGVLMLAIQASWLYWYIKRQP----KEEVPRKHLYDRVQQVGELQD---------------------------------------------------------
         sea lamprey     --M----FYVIVFSALFGIVFTFMLEVFIYYR-LGLKPAKSVVVGPREPTVSSRPGGGEA------KGVPGAQGQGQVTSS---------------------------------PDGLSA
               human     MGL----LLMILASAVLGSFLTLLAQFFLLYR-RQPEPPADEAARAGEGFRYIKPVPGLLLREYL----YGGGRDEEPSGA------------------APEGGATPTAA----P-----
             opossum     MEL----LLIIAVSALAGSFLTIFFQFLLFYCSRPKKP----VAASSADLIYTSPVPGLHLRDYL----YGGFGKKRKSSG---------------------------------SGVGHR
             chicken     MLP----VYTILLSALAGCLLTLLLQLLLLYR-KAPQPPG--GIGAEDGLVYARVAAGRSLKDYL-HGTEPVGGQAAPEPAPATATASASAPAASAPSAGPEPGTKPAQQ----PEP---
tropical clawed frog     --M----VYAALVSALAGCLLTLLLQLLLLFRHRRPEP-------LARPPPPRPVQPDASLRDYL------------------------------------------------------K
         coelacanth     --M----IYLILLSVFAGAFFTLLLEFLLLYR-RKPEP-------VPRTVQYVKPVPDPLLKDYFNNNKNSDSGQQQQDFA-APTPSKHQEAAGVK------------------PQP---
        Nile tilapia     --M----IYLILVSAAFGAVVTLIVQFLLIYR-RSPEP-------VGRTVQYVKVVPGNALKDYF-NSHHAEAGQQQDSTT--CTASKQPEAASTRQQEAAVTGGGPKQQ---PPPPSQS
           zebrafish     --M----IYLILLSAFSGAVVTLLLQLLLLYR-RSPEP-------VARTVQYVKVVPDPALKDYF-SSQQADSAPQQPDSP--SPVSKQPEAASPKQQETPVPGSSPKQQPSSPPPPSLG
         spotted gar     --M----IYLIFISAFLGAIATLLLQFLLLYR-RSPEP-------TARTVQYVKTVPDPALKDYF-INKHAESGPQQPENA---ASKQQDGIALSRQQEGALPSSSPKQ-----PPRQQN
        little skate     --M----IYIILLSALAGALCVVVLQILLLYR-NKPEA-------VPRHVQYVKPVVEPSLKDYLSGAKERPASPESTSSA---------------------------------P-----

                                130       140       150       160       170       180       190       200       210       220       230       240
                         =========+=========+=========+=========+=========+=========+=========+=========+=========+=========+=========+=========+
                                 ***************************************** *****************************                            *************
     polychaete worm     DPNLISKRESCIAVNLIFQMLFRELKDSKIVRRWVMRRMQLEFTELLYT-TTGKLLDQITVRDYNLGDTLPVIRYVSVLD-VNMKDD-------------------SLLEEVTLLVDLTY
         human louse     HCNQLVTKESCVSLNLMLQFLFHELRDTEKIRLWFRKKLCIEFEELLSKSTIGKMVGNIKIVEIDLGSEFPVLNNISIQDVKVNKDY-------------------SHIDVLDLKLDVNY
          sea urchin     PSSILFKKESCNTANALISFLFNELKDTLALRRWIIKKMNVEFGELLNAKTAGKIMEQITVRDYCVGPTFPTLTGVTLMKFTTVEGT-------------------DMPETVDVAVDIDY
         sea lamprey     GEPPPGTPESCDFLNVLFLFLFRELRDTGTVRRWLIKKIRVEFVELLQSKTVGRLIEGLSLRDVSLGDTLPVFSSVVMHEPDPVPEK-------------------NVPEQLDLEVDVEY
               human     ETPAPPTRETCYFLNATILFLFRELRDTALTRRWVTKKIKVEFEELLQTKTAGRLLEGLSLRDVFLGETVPFIKTIRLVRPVVPSATGEPDGPEG------EALPAACPEELAFEAEVEY
             opossum     TSKMSRAVETCYFLNAIILFLFRELRDTTETRRWVTKKIKVEFQDLLQSKTAGRVLEGLSLREVYLGQAVPFVRTVRLLRPVVPSYT----GEPEASTKSHEELPTACPEELIFELDIEY
             chicken     GPEPSSSEETCHFLNAIFLFLFRELRDTALVRNWVTKKIKVEFEELLQTKMTGKVLEGLSLRDVYLGNVLPVFKAVRLIRPVVCSEE-------------------GCPEELGFEVDLEY
tropical clawed frog     AEPGGPAAESCQFLNTIFLFLFRELRDTALLRRWLSKKIRVELEELLQSRTAGRLLEGLSLRDISLGEALPVFRSVRLL---SAPGE-------------------PLPDELQFEMEMEY
          coelacanth     ESANPFKAESCHSLNAIFLFLFRELRDTPLVRHWVTKKIKVEFEELLQTKTAGRLLEGLSLRDISLGNALPVFKTAQLLKPVACNED-------------------GMPEELNFEIDIEY
        Nile tilapia     EPIDAGKAETCHFLNAIFLFLFRELRDTPVVRHWLTKKIKVEFEELLQTKTAGRLLEGLSLKDFSLGNSLPVFKTAKLMKPVHVNED-------------------GMPEELNFEVDIEY
           zebrafish     DPQHSSKAETCDFLNAIILFLFRELRDTPVVRHWITKKIKVEFEELLQTKTAGRLLEGLSLRDVSLGNSVPVFKTARLMKPVAVNED-------------------NMPEELNFEVDLEY
         spotted gar     DSAHTSKPETCNFLNAIFLFLFRELRDTPLVRHWVTKKIKVEFEELLLTKTAGRLLEGLSLRDISLGNSLPVFKTAKLMKPVSCNDD-------------------GMPEELNFEVDLEY
        little skate     GNGGSDKSDSCHFLNAIFLFLFRELRDTPIVRHWLTKKIKVEFEELLQTRTAGRLLEGLSLRDISLGNALPVLRSARPLQPSPVTANSSGGGGTSNSSGGQDDEAGGVPDELNFEVELEY
```

```
                            250       260       270       280       290       300       310       320       330       340       350       360
                   =========+=========+=========+=========+=========+=========+=========+=========+=========+=========+=========+=========+
                   ********************************************************************************************
   polychaete worm NGGFQLAIDLDMVFGKSAYLSVKVTKLSGLARLQFSRNPYTHWSFSFVDEPVMEFNVESHYEGRPMPQITSLIINQIRRSIRKKHTLPNYKLRYKPFFTKPEE----QTPPQDIYLHGNK
       human louse SGGFRLVVEANMLLGKAALISIQINELYGEGRLEFTRLPYSHWSFTFFNEPKIELHVESYFQGRPFQQITNFISSQIKKTLKKKHTLPNYKIRYKPFFTSSQN-----------STQPEQ
        sea urchin GGGFRVSVDVDLVFGTSAYVSVTVTKLKGRARLEFTRNPYSHWSFSFYEEPELEFDIQTHFEGRTVHQLGSLILNQLRKTMRKKHTLPAYKIRSKPFFPKPTPP-----SDNDFRVHDSK
       sea lamprey SGGFHLAVDVDLVFGKSAYLFVRVARVAGRVRLRFSHLPFTHWSFAFIREPELEFDVESRLEGRPLPQLTAIIERQLRSAICRKHTLPNYKVRSKPFFPVRVVP-----RDIDLCMQDSK
             human NGGFHLAIDVDLVFGKSAYLFVKLSRVVGRLRLVFTRVPFTHWFFSFVEDPLIDFEVRSQFEGRPMPQLTSIIVNQLKKIIKRKHTLPNYKIRFKPFFPYQTLQGF-EEDEEHIHIQQWA
           opossum NGGFHMAIDVDLVFGRSAYLFVKLARMKGKIQLIFTRTPFTHWAFSFVEEPVMDLEVQSQFEGHPMPQLANIIINQLKKVIKRRHTIPNYKIRFKPFFPYEMT----EESEEILYIQQYG
           chicken NGGFHLAIDADLVFGKSAYLFVKLSRVMGKLKLVFTRLPFTHWYFSFMDDPLIVFEVKSQFEGRPMPQLTSIIVNQFKKVIKRKHTLPNYKIRYKPFFPFQVSSPD-EYEDRDLYIQDLP
tropical clawed frog SGGCRLAIDVELVFGKSASLSVRLGRVIGRLRLVFSRLPFPHWAFCFLPDPTIDFHVQSQFEGRPMPQLTSIIVTQLKKVIKRKHTLPNYKIRYKPFFPIQVQPPETDLEERSLFLQDMG
        coelacanth NGGFHLAIDVDLVFGKSAYLFVKMTRVVGRLRLQFTRLPFTHWSFSFLEDPLIDFEVKSQFEGRPMPQLTSIIVNQLKKVVKRKHTLPNYKIXX-------------------------
      Nile tilapia NGGFHLAIDVELVFGKSAYLFVKMRRVVGRLRLQFTRVPFSHWSFSFLEDPLVDFEVKSQFEGRPLPQLTSIIVNQLKRVIKKKHTLPNYKIRYKPFFPFQVQPLHGSACDLDLSVHDSR
         zebrafish NGGFHLAIDVDLVFGKSAYLFVKMTRVAGRLKLQFTRMPFTHWSFSFLEDPLIDFEVKSQFEGRPLPQLTSIIVNQLKRVIKRKHTLPNYKIRYKPFFPFQVQPPLMSSCDLDISIRDTL
       spotted gar NGGFHLAIDVDLVFGKSAYLFVKMTRVVGRLRLQFTRLPFTHWSFSFLEDPLIDFEVKSQFEGRPLPQLTSIIVNQLKRVIKKKHTLPNYKIRYKPFFPFQVQPLLGQTSDLDISVQDSR
      little skate NGGFHLAIDVDLVFGKSAYLFVKMRRVVGRLRLQFTRRPFTHWSFAFMDDPFIDFEVKSQFEGRPMPQLTSIIVNQLKRVIKRKHTLPNYKIRYKPFFPYQVLPATADTVNCDLYLQHAN

                            370       380       390       400       410       420       430       440       450       460       470       480
                   =========+=========+=========+=========+=========+=========+=========+=========+=========+=========+=========+=========+
                                                                         ***************      *******************  ************************
   polychaete worm VSIGQLEVTVVDCNRLV---EITEEGFIYVSLAVDSLPWTEIMSSRRKIWVTHELEIVKDKSHNIGLTFREDFILGRFEEMVVIESVTAKSPAS-KSDLKRNDLLLSIGGVKVINSKQAV
       human louse IPNGQLHMIIHKVTRLNEIAPEMSE--VFCTSGIGRVPWIDIINYGKNNYYLIDIVITKSVFQTMGFVFKQEYLEDKNQNCILVEDLNQNIPSSREVGLKREDIIVFIDGKKITSINQVQ
        sea urchin ITLGRMQVTVIECSRLPV-SKETMNSELYITLCLDDHAWTHEDIEQRFHGDSLEIEVPKGNMKNVGVTFSR----GLYNKEVVVASISPESTVA-QTDLRKGDVVLDIDGVKVTSARQAA
       sea lamprey LSEGRLRVALLECSRLYIPGSQDREVAVHCTLELSAGQWKE-----RERSAVREVEIVKVGTAPIGLSFRQTQSTDGESSQVLVEAIAANSPAS-SANIRRGERLVTIGGTKVATSSQAS
             human LTEGRLKVTLLECSRLLIFGSYDREANVHCTLELSSSVWEE-----KQRSSIKTVELIKGNLQSVGLTLRLVQSTDGYAGHVIIETVAPNSPAA-IADLQRGDRLIAIGGVKITSTLQVL
           opossum LKEGRLKISLLECSRLMIFGSYEREANIHCTLELSSSIWEE-----KQRTSVKVVEVVKGNLPSVGLTLRLVQSSDGCSGHVVIETVAPHSPAA-LADLQRGDRIVAIGGIKITSTVQVL
           chicken LTEGRLKVTLIECTRLFIFGSYEKETNVHCTMELSSSVWEE-----KSRSSIKTAELVKGNLQSVGLTLRLVQSKEDDAGHVVIETVTPNSPAA-AADLQRGDRLLAIGSVKITSTVQVL
tropical clawed frog LTEGRLKVSVTECSRLLIFGSYDQETYIHCTLELSASPWKE-----KTRTSIKTVELIKGNSPSFGLIM--VQARDGDTGHVCVESVTPNSPAA-SVDLKKGDRLIAIGGIKVTSSIQAS
        coelacanth ----------------------------------------------------XXVELIKGNSQSVGLTFRQIQASDGDAGHVCIETVTPNSPAA-VADLQRGDRLIAIGGTKVTSSVQVL
      Nile tilapia LVEGRLKVSLIECSRLFILGSYDRETYVHCTLELSSHQWKE-----KARSSIKKTEIIKGTCGSVGMTFRHVTASEGDAVHVCIETVLPNSPAA-LAELQKGDRLIEIGGVKVTSSVQVP
         zebrafish LVEGRLRVTLVECSRLFILGSYERETYVHCTFELSSDEWRE-----KTRSSIKETEVIKGPSGSVGMTFRHVPASDGDTVHVSIETVTPNSPAA-LADLQRGDRLIAIGGVKVTSSVQVP
       spotted gar LVEGRLRVMLVECGRLFILGSYDRETYVHCTLELSSSEWKE-----KARSSIKTAELVKGSSQSVGLTFRQVPANEGDAVHVSIETVVPSSPAA-LADLQKGDRLIAIGGVKVTSSVQVS
      little skate MTEGRLKVTLVECSRLFILGLYDRSETIHCTLELSSDVWKE-----KTRSSIKTVELIKGNSQSVGLAFRQIQGNDGDAVHVTVETVTPNSPAA-AADLQKGDRLIAIGGTKVTSSVHVL
```

```
                            490       500       510       520       530       540       550       560       570       580       590       600
                   =========+=========+=========+=========+=========+=========+=========+=========+=========+=========+=========+=========+
                   *******************                                                      *******
    polychaete worm KLMKQATDRFVVKIERSSS---------LKGGVPSSLL---------------------------NSVSEVKVEEDVLSFKV-E-----ASDAP-------------ELKQTEVSDDYVN
        human louse KYIKSAAVQFTIRAERKIR-----------------------------------------------------DKVIENFND-------------------------------------
          sea urchin KVVKQAKEKFVIKVQRPMN---------MMESLPATSAAYDQPDL--------------------RSVPDTTADNDYEDFINVN-----IMNEL-------------GIKDEDEVDGSQP
        sea lamprey KLIKQAGERVIISLERTFG-GVQPPPGSASGPLPHGMHVADGSAIIPD---DDSSKDTVSLGSV-ESNDAREGDSDVDEGECGDGFPESLRSKEEPPPSLLSVPPTKDETPQPPSTASPS
              human KLIKQAGDRVLVYYERPVG-QSNQG-A----VLQDNFGQLEEN-FLSSSCQSGYEEEAAGLT---VDTESRELDSEFEDLAS-D-----VRAQN-------------EFKDEA-QSLSHS
            opossum KLIKQAGEKVMVCYERPVG-QNNIG-S----SSHESFTQEE---FLTT--PPNDDDIMPGLL---MEAERREADLEFEDLAS-Y-----LKSQN-------------YSKDED-QTISSS
            chicken KLIRQAGDKVLVFYERPVG-YNQHG-S----ALQDGFGQLEDTAFLTQQ-----EDDQVSVS---ADVDSRDFDSEFEDLAC-E---------S-------------PDQKED-MPTTPS
tropical clawed frog KLIKQAGDRTLVFYERPAG-QNQSA-G----SLPENLGQLEDPAFAASAFPQTFEEDSVSFI---VDPENKELDSEFEDLAN-E-----VKPPG-------------ESKEDS-VSVTQS
        coelacanth KLIKQAGDRVMVYYERPVG-HQNQH-G------SESFGQLEDTGYMLQSCPQSYEEDTVSITNL-DSGDGKDVDSEFEELVC-E-----VKPTN-------------EFKEEPLPTVTQS
      Nile tilapia KLLKQAGEKVLVLYERPVR-HQTPSLG-NQAALQEGLVQLEEIGFMSQ--QAGYEEEPAPISTLSDLSDSKDIDSEFEELIV-E-----SKPSGGSAGQNSSTTSISENKEDFLLTVNQS
         zebrafish KLLKQAGERVIVLYERPVR-HHVPT-G-GLGMLQETLGPMEEPSYLPQ--PGGYEEDPAPITTM-DISENKDNDSEFEELNV-E-----SKTAA--------APVTIDTKEDFLLSVNQS
        spotted gar KLLKQAGDKVIVLYERPVR-HQMPPVG----VLQEGFSQFEEPSYLSQ---PVYEEDPATITTL-DPADSKDVDSEFEELVC-D-----IKSTN------------TEAKEESLLTVNQS
        little skate KLIKQAGDKVLVYYERPVGGHRNQQCG----SLQENCGQLEDTGFSAQFGQHAFDDD--GIL---DNVDGKDLDSEFEELVCPE-----IKPPT-------------IIVKEDFAPSNLS

                            610       620       630       640       650       660       670       680       690       700       710       720
                   =========+=========+=========+=========+=========+=========+=========+=========+=========+=========+=========+=========+
                    ******      ***   ******                ****                  *******
    polychaete worm ITVADHSEVSIP-SLDATTQEESSSNASPV----------SSRRSFSIL------------PSSSGLFHRKRSTSSRGSPEVKDKVTTDPKVTIEAQK--VKAKLSINTDFST-------
        human louse -NIESHSLPSSPLSLS---LRRRKGSES------------DGCSINSTL------------SSSSNKSQMKRPASDIGKP---------------------------------------
          sea urchin GSIAAQRL--KVPTLDSPVVQRKNKQPTTA----------DGRRLSSCLDYETVKQLSTQQDNEDRLRNLKRVIKETIHPTPRDERPKEDPVSAVPMM--MKTEAMRIPDVDL-------
        sea lamprey KRRTMAT-----LGSISPILSRRLQQSS---PLRSLHKGYDAAKLGSTT--------PAQPDGEKVAPSASAKPGLVNQSGRPPPIPPRPQLRATSSAPLGSLQSLAETGDAS-------
              human PKRVPTTLSIKPLGAISPVLNRKLAVGSHPLPPKIQSK--DGNKP-PPL------------KTSEITDPAQV-SKPTQGSAFKPPVPPRPQAKVPLPS--ADAPNQAEP-DVL-------
            opossum PKHCLVSVA-KPSGPASPVLNLKSGGIS------------RSVKS-MTP------------KISDSSDIGQASAKATQVSLFKPSQSPKQQRKFFPPS-IDDSSNQAESD----------
            chicken PKRSVVILAAKPLGTISPILNRKLHLGNYQTTSKMQQK--DGAKL-ATP------------RTSDSSDTSQLSGKQSQGSGTKPPVPPRPQIRPTLPT--NETQNLLETGGVS-------
tropical clawed frog PKRTVATLYTKPLGSVSPILNRKANLPSHQTPSKTQPK---VNKT-APP------------ETQDSTPQNRAS------PANKPPVPPRPQVKLTLTS--CENPSAPETGEAT-------
        coelacanth PKRSVATLAVKPLGTISPILNRKLNLVNYPAPLKPQQK--EGAKP-TQY------------KGSEHQEGTPQPNKTSQGSTNKPPVPPRPHIKPNLAS--SESQCVLETGDTF-------
      Nile tilapia PKRTVANLASKPLGTISPILNRKLNLVGLQSPLKPQPK--ESPKPSPQK------------TSSDPGEVLQRPTVPPPPPPSRPPVPPRPQIKLTSAS--SET-SLLESVDSAAAGNVSA
         zebrafish PKKTVANLA-KPLGSISPILNRRLNL---QSPLKTQPK--ESPKP-PTL------------KNAEPSEQPQRPTVPPPPPPARPPVPPRPHIKVTSAS--SEAQSLVEGNEPT-------
        spotted gar PKRTVVNLATKPLGTISPILNRKLNLASLQSPLKPQPK--EVSKP-PLS------------KSSEVSEAPQLQSKPPQTVPNRPPVPPRPHIKLTLAS---ETQCLLDSGDAP-------
        little skate PKRSVATLATKPLGTISPILNRKLNLGNYQIPLKSQQK--DAAKF-AQP------------KCLDVPDGAQQPSKPALGSTNKPPVPPRPQIKIISAA--SEAQNLSESADTA-------
```

```
                               730       740       750       760       770       780       790       800       810       820       830       840
                        =========+=========+=========+=========+=========+=========+=========+=========+=========+=========+=========+=========+
                                                                                                                    ***********     ************
     polychaete worm    ------------------------------RRKCKSESNLGSQTVENPAVDSISVAE--STASSNVSEASEIPEAFELLK-----------TQFVRAAQDPVFQ----EKFAFDIAERHA
         human louse    -----------------------------------------------------------SNSLIETINEEEAENILKLYK-----------SKVLFNKQIIQFE----EVVICPVKNDDR
          sea urchin    -----------------------------------DAISVSSLTSQMEDVDAASVGP--SVSSAPAGSEAAPPPVDDIQE-----------TYLVAAEANPEWD----ETFPFQVGENDN
         sea lamprey    ----------TLKPTDKGG---------------------SMEQVEQEKLGDATDSG--AVGASGLACAEECTDELCVTQ------------EMVYHSNRAVWPARA-ASHTFDVESHHR
               human    ----------VEK-PEKVVPP-PLVDKSA-EKQ----------AKNVDAIDDAAAPK--QFLAKQEVAKDVTSETSCPTKDSSDDRQTWESSEILYRNKLGKWTRTR-ASCLFDIEACHR
             opossum    ----------SEK-PDKIL----------------------------------------YSTQAFDQTEDSILEST--HKDSADD-QTWESSEIPYSHKLGKWTRMK-GTCIFDIEAYHK
             chicken    ----------SEK-TEKPPPSSGNGDKCT-EKV----------VKNNDQTEDPTLSK-VATIPKQDALKDGISENARGHKDGGDDHRTWESPEIPYRVRQGRWPKARTSSCLFEVEGSHK
tropical clawed frog    ----------VEK-PERPPPPSLNGDKQP-EKS----------AKTTETVDEAPVQK--PSSVKQDVAKDKISESSCSTKDSVDDPHIWESKEILYRNKQGRWT--R-SSCLFDVENYHR
          coelacanth    ----------VEK-TEKTPTPSSNGEKSA-EKTS---------AKNIDLIEDLGTSK--TCTSKQEAVKDKISESSCSTKDSMEDRQTWESSEIPYRNRYGKWN--K-ATCLFEVENHHK
        Nile tilapia    ASSITKTTSASEKSPEKVQRSGPGEDKPATEKACVKQMEAKQSSKAMDTSEEVLPSTVTSSSSKADQAKDKASESSSSTRDSVEDHSLWESTETMFRNQTAHWS--K-ASVVFEVESNHK
           zebrafish    ----------VEKSPEKTQPNTGNGEKTV-EKIPVKPPEPKPVSKHPEPTEDILN---IPATNKQDSAKDKISESSSNTRDSVDEQGLWESSETMYRNRTARWN--K-ASVIFEVESNHK
         spotted gar    ----------QEK-PERPPPPLSNGDKPA-EKQG---------GKPAEQSEELGL----SNLNKQDLTKDKVCESSCSTKDSVEDNTVWESSEATYRKQIARWN--K-ASSVFEVESHHK
        little skate    ----------TEK-LDKSATSINSAEKSI-EKGV---------SKNLDQMEDASQSR--PTASKQD-SKEKLPETTCRVRDGAEGNKIWESPEISYRKRFVKWN--K-ATCVFDVEGRHR

                               850       860       870       880       890       900       910       920       930       940       950       960
                        =========+=========+=========+=========+=========+=========+=========+=========+=========+=========+=========+=========+
                        *************           ***************      ****************   ******              **********
     polychaete worm    YLHVCIWCKIPEKLDKQQRVIKPERDILLGHTSLSLMEIALECLNTVQGEVQMTRKLTPGDVKASVSQSQ-----GALHPGFD-ERLCYGDVTLDIHHIP---------NHLSKSDRRTI
         human louse    FININVKYNECNSN----------TDKLLGFVNIPLSEV------LNAYDTLRCLKLKPPD---SFSASNVRNHPLLSHNGFI-PLLCYGDIILTLQFHP---------------SPDVI
          sea urchin    FLNVCIWNRLGMAVG--------ERDMLIGYTSVCLMDVALQCVSTLSGEHLELFKLQPPEQRGGATRIASQMH-LFTHPGFD-SSLSYGDISLFFHHSPFEDPPKPTKEGGAERPDYSP
         sea lamprey    SLTVAVWRKDPLKPD---------SLRCLGYASVPLMEIAAECLATASYEHEEVFPLSAPEPRATANRTAMRN--ANAKEKQNLEQPYYGDVRLRFAYWV---------DEGGDGLTSSS
               human    YLNIALWCRDPFKLG---------GLICLGHVSLKLEDVALGCLATSNTEYLSKLRLEAPSPKAIVTRTALRN--LSMQKGFN-DKFCYGDITIHFKYLK---------EGESDHHVVT-
             opossum    YLNVALWCRDPFKLG---------DLICLGHVSIKLEEIALGCLATSNLEYLTKFQLNAPTPKAMASRSTLRN--LSMKTGFN-ENFCYGDITIQFKYLK---------EGEHEQLLDL-
             chicken    YLNVALWCRDPFKAG---------GFICLGYASIKLEEIALDCIATSSMEYVRTFKLSAPTPKAAVTRTALRN--LTTHKGFN-EKFCYGDIALHFKYFK---------EGEVDDSNFL-
tropical clawed frog    YLNVALWCRDSFKTG---------SLCCVGHISIRLEDIALECLATASLEYLTSFKLNPTEPRASVSRTALRN--LSMHKGFN-EKYCYGDVTLNFKYLM---------EGESETSSML-
          coelacanth    YLNVALWCKDPFKVG---------DLICLGHISIKLEEVALECLSTSSMEYQTTFRLNAPEPKAVVSRTALRI--LSTHKGFN-EKLCYGDITLNFTYLK---------EGESENSSFV-
        Nile tilapia    YLNVALWCKDPFKLG---------SLICLGHVSLQLEHIALECMATSSGEYQSTFRLGAPEPRANVSRTALRS--LSTHKGFN-EKLCYGDVTLNFCYLA---------EGELDYPVGL-
           zebrafish    FLNVALWCKNPFKLG---------SLLCLGHVSLRLEHLALECISTSSAEYQSTFRLCAPEPRASVSRTALRS--LSTHKGFN-EKLCYGDVTLNFTYLA---------DGESDLSSGL-
         spotted gar    YLNIAVWCKDPFKIG---------SLLCLGHVSLKLEHIALECLSTSSMEFQTTFRLNAPEPKANVSRTALRN--LSTHKGFN-EKLCYGDITLNFSYLS---------EGESEHSSGL-
        little skate    YINVAVWCKEPFKSG---------ELMCLGHASIKLEEIAMECLCTASMEFQTTFRLNAPEPKAVVSRTALRN--LSTHKGFN-EKLCYGDVTLNFTYLK---------EGDTENSSIQ-
```

```
                              970       980       990      1000      1010      1020      1030      1040      1050      1060      1070      1080
                     =========+=========+=========+=========+=========+=========+=========+=========+=========+=========+=========+=========+
                       ******                  ******                        ***************************************************
    polychaete worm  HRVKEENERITELE----KIAEPEPSAVIKRR-----------------SLGKPGEEGKHDFVSAHFQVATYCNFCHKKIWLKTAFQCKVCSMVCHKKCTEKCQAQTT---CAKDGFHFK
        human louse  GNVKSVPEII-------------EPSSVTPES----------------ETPSQDFEERKHDFVKTHFQTSTQCEFCGRKIWLKDAEKCKICGMTCHKKCVAKCKANKI---CV-------
         sea urchin  YDAKGKPKVSVEEK----------GKPVVVKK-----------ITRPEELSLHGQVGQPHKLVGTQFSSPTRCDFCGKKVWTKYALQCLICKLICHKKCSEKTQANIP---C--------
        sea lamprey  ANVDEDRDLFTRAE-------EVGPVPAGVRDWDSGPPAAPPPPLPPLPPSSGMGGAARHSFCDTQFQNPTYCDYCKKKVWTKAATQCSRCAYVCHKKCQERCLEESESGRCM-------
              human  -NVEKEKEPHLVEE-----------VSVLPKE--------------EQFVGQMGLTENKHSFQDTQFQNPTWCDYCKKKVWTKAASQCMFCAYVCHKKCQEKCLAETS--VCG-------
            opossum  --LKKERETHLSEE-----------EDAFNRD--------------EYFDGQLGSAENKHAFQDTQFQNPTWCDYCKKKVWTKAASQCVYCAYVCHKKCQEKCFSENP--ICI-------
            chicken  --LEKEKECHLEEE-----------VNVLQKE--------------DTYLGQIVLTENKHNFQDTQFQNPTWCDYCKKKVWTKAASQCMLCAYVCHKKCQEKCLAETP--FCV-------
tropical clawed frog  --SERERDVGFQDD-----------GSSLQKE--------------ELPVPPMIFAESKHNFQDTQFQNSTLCDYCKRKVWTKAASQCINCGYVCHKKCQEKCLNENP--YCV-------
        coelacanth  --IEKERECNVQEE-----------VPVFQKE--------------EPNLGQMFFMENKHNFQDTQFQNPTWCDHCKKKVWTKAASQCMLCAYVCHKKCQEKCLAENP--FCV-------
       Nile tilapia  --VERDTEGSVQEEDL----REREPVPAAPRE--------------DLTYSALQLQEVRHNFQDTQFQNPTWCEYCKKKVWTKAASQCMVCAYVCHKKCQEKCLTENP--FCV-------
          zebrafish  --TERERKGSLQEEDLKDREKEREQVLMVTRD--------------EPIYSGMQIGEMRHNFQDTQFQNPTYCEYCKKKVWTKAASQCMICSYVCHKKCQEKCLLEHP--YCV-------
        spotted gar  --VEREGECGVQEE-----------ELLLPKE--------------EVGLSAMQNSEVKHNFQDTQFQNPTWCDYCKKKVWTKAASQCMICTYVCHKKCQEKCLSEHP--FCI-------
       little skate  --IEREKEDHLQED-----------IPVIIKE--------------ETDPSCM---DNKHNFQDTQFQNPTWCDYCKKKVWTKAASQCMNCAYVCHKKCQDKCLAEAL--LPG-------

                             1090      1100      1110      1120      1130      1140      1150      1160      1170      1180      1190      1200
                     =========+=========+=========+=========+=========+=========+=========+=========+=========+=========+=========+=========+
                                                                                                  *************
    polychaete worm  EPRQST------P----PALKKVIQNKDTSSTSSKESSGSPKTSFLSKLRKEGSKAVFPAKGVQAIPVTIAPSSSFVEGMDRKRHNSAPDVDQELSDGGAQGLDLL-----SEREDQHLV
        human louse  -PRSDSTVGNIGENYNYEEDGVRASLTPEPGSSSRESTPQPTPT--------------------------------KKRLGNLLATVASR---------------------GLKRVGSAN
         sea urchin  -----------------------------DRNRSARRADQPSPT------------------------------R-ELGQTRPLDSAAQT--------------LV-----G--------
        sea lamprey  -PRESR------E-------GAREGGAAGSGAAAAGSGGGDPPA------------------------------R-LSNLKQSVAMARSR--------------LTLAVPRPLRQA----
              human  -ATDRR------IDRTLKNLRLEGQETLLGLPPRVDAEASKSVN------------------------------K-TTGLTRHIINTSSR--------------LL-----NLRQ-----
            opossum  -GAGKK------SNRLFKNLRLEGQENIIGLQSRADSEASKSAG------------------------------K-SAGLTKHLLNTSSR--------------FL-----NLRQ-----
            chicken  -GAEKR------HDRTAGGCRAEGQETSQAASVRVDSESKS-VN------------------------------R-TAGLTRHIINTSTR--------------LL-----NLRQ-----
tropical clawed frog  -ASDKR----------------------------VDPESKSLGN------------------------------R-TTGITRHIINTSSR--------------LL-----NLRQ-----
        coelacanth  -ATERR----------------------------VDFESKSTIN------------------------------R-TTGLTRHILNTSSR--------------LL-----NLRQ-----
       Nile tilapia  -ATAER------R--------------------GADLEAKSTIN------------------------------RATTGLTRHIINTSSR--------------LL-----NLRQ-----
          zebrafish  -AASDR------R--------------------GADPEAKSTIN------------------------------RATTGLTRHIINTSSR--------------LL-----NLRQ-----
        spotted gar  -PADRR----------------------------VDLEAKSTMN------------------------------R-ATGLTRHIINTSSR--------------LL-----NLRQ-----
       little skate  -LTERR----------------------------ADLEAKPSFN------------------------------R-STGLTRHIINTSSR--------------LL-----NLRP-----
```

```
                              1210      1220      1230      1240      1250      1260      1270      1280      1290      1300      1310      1320
                        =========+=========+=========+=========+=========+=========+=========+=========+=========+=========+=========+=========+
                                            **********************            **  ***    ** ******        **************************************
      polychaete worm   VNSLPRTRSSSTLEDL---IKENAPPALAGDEYDQNDDSETT-SSADSVDSEDDEE-LNLLKFYERQKASSSQN----ADELVVTAAKEMGRELYANMSPEERREKLDHMVSKLQQEIDQ
          human louse   NLTLPGDASSNSQSR----SLPPSPQQSPSPSRKSSLPLELS------------EE-ISELLQALMENGSDSGD--------MMDFAKETGKTLYEDLKLDERKDKINEMINKLNVAINA
           sea urchin   ---VAGRRGTPH--------PSPNPSPAPSPHESEEEEDEEV-DVEAMV-----RR-LSKLKRRLRQLNAAEGNRVDDTDAMVMTAVREMGRELFIDSPVDERKEKLEAMIEKLQTEIDE
          sea lamprey   ---GSRLRASEE--------VVVGPTQSGGTSDNESSDNEGG-SSGSAVGPTGQPS-ASARA--MEQ-ASLPGR----LEDSVYLAVKEIGREIFRGMPAEERRSKLEQMLDKLQQEVEA
                human   ---VSKTRLSEPGTD----LVEPSPKHTPNTSDNEGSDTEVC-GPNSPS-----KR-GNSTG--IKL-VRKEGG----LDDSVFIAVKEIGRDLYRGLPTEERIQKLEFMLDKLQNEIDQ
              opossum   ---GSR-RTPDQINE----LAEPSPKHTPTTSDNEMSDHEVC-GLSSPC-----KK-GTDRR--IKL-ARKERG----LDDSVFIAVKEIGRDLYRGLPIEERFQKLEFMLDKLQNEIDQ
              chicken   ---VPKARLSEQGTE----IVEPSPKHTPNTSDNESSDTEIS-GPSSPS-----KR-ASGSG--IKL-VRKEGG----LDDSVFIAVKEIGRDLYRSLPTEERFQKLEFMLDKLQNEIDQ
tropical clawed frog   ---APKLRQPEQGCD----LVEPSPKHTPNTSDNESSDTETC-GSSSPS-----KR-ASGGS--GKL-ARRDGG----LDDSVFIAVKEIGRDLYRGLPTEERIQKLEFMLDKLQNEIDQ
          coelacanth   ---APKARLAEQGAE----AVEPSPKHTPNTSDNESSDTETY-SGSSPS-----KR-ATSTG--IKL-ARKEGG----LDDSVFIAVKEIGRDLYRGLSTEERSQKLEFMLDKLQNEIDQ
         Nile tilapia   ---VPKARFTEQVADVVPGVVEPSPKHTPNTSDNESSDTETYTSGASPS-----KQ-PAGSGGSSKL-VRKEGG----LDDSVFIAVKEIGRDLYRGLPTDERSQKLELMLDKLQQEIDQ
            zebrafish   ---VPKARLAEQVADMGSGVVEPSPKHTPNTSDNESSDTETY-TGASPS-----KQPAGSSG--SKL-VRKEGG----LDDSVFIAVKEIGRDLYRGLPTDERSQKLELMLDKLQQEIDQ
          spotted gar   ---VPKARLTEQGGD----MVEPSPKHTPNTSDNESSDTETY-MGGSPS-----KR-PVTSG--NKL-VRKEGG----LDDSVFIAVKEIGRDLYRGLPTDERSQKLEFMLDKLQHEIDQ
         little skate   ---GQRARLADPSVD----LVEPSPKQTPNTSDNESSDTETY-SASSPS-----KRVASSMG--NKLSVKKDGG----LDDSVLIAVKEIGRDLYRGLPADERFQKLYLMSEKLQNEIEL

                              1330      1340      1350      1360      1370      1380      1390      1400      1410      1420      1430      1440
                        =========+=========+=========+=========+=========+=========+=========+=========+=========+=========+=========+=========+
                        ***************    ************************************** **
      polychaete worm   ESENRTELARMEREL----GLPTQRAVARAKLEKSDEKIQSLALLMLHYCAGLQHCIEQQEIEIDKS------MENECSENEMQGLVVVDK-------------------------PA--
          human louse   EADTRTTLVREEQVE----KDQTNKAKIAFLIGKSDEKTQALAILMLHFCAGLQHIQDLED----------QEKENEMAEEIKLPPTSSSS------------SSFGEIK----------
           sea urchin   EADRQLRLAKEVQLS----RRRKEKLALEGALAQSEERMQTLANLMLHYCAGIQHCNETL------------------------------------------------------------
          sea lamprey   ELEQRNTLEREERQACAAGSDAKKVANVTHSLRKSTERLQALTLLSIHYRAGMEEEDQSEVGCVPTGG---PSTAHRLPQPPQAVSSVLTQLPPDVPLHPAESEQFHTLQDPSLFPSAPR
                human   ELEHNNSLVREEKET----TDTRKKSLLSAALAKSGERLQALTLLMIHYRAGIEDIETLESLSLDQH----SKKISKYTDDT---EEDLDN----EISQLIDSQPFSSISDDLF-GPS--
              opossum   ELEHNNSLAREEKET----TDSKKKAHLLSSLAKSGERLQALTLLMIHYKAGIEDLE-LETLYNTKK----AAKYTEETEEDL------HN----EIRQLIVTQPFDSLSDELF-EPS--
              chicken   ELENNNSLVKEEKEA----TEARKKALISAALAKSGERLQALTLLMIHYRAGIEDIESLESMSSDRQ----SKRVTKDSEEASIAEEV-DN----EDIGLTEAPTLNIISDESV-DPP--
tropical clawed frog   ELEHNNSLVKEEKDA----TEARRKALLSAALSKSSERLQALTLLMIHYRAGIEDIESLENSSLAQP----RKNAKCE-EEAVVAAEADNHDEDEDDVGQAESETLSCVSPEDKLPTS--
          coelacanth   ELEHNNLLLKEEKGA----TDARKKSLLCAALSKSSERLQALTLLVIHYRAGIEDLESLESMSSDQQ----SRKTRCS-EESSLTTDVYEN----EVPNQMDTQTFNNIPDEQIFDES--
         Nile tilapia   ELEHNNALLLEEREA----TDARRKALISTALAKSGERLQALTLLMIHYRAGIEDLESVESTSPSEQRGF-KGKEEGLEEEALLGTEVYDS----DICSPVDVPLLDDITEEQICVEA--
            zebrafish   ELEHNNSLSTEERDT----IDSRRKTLITAALAKSGERLQALTLLMIHYRAGIEDLESVESTSPSEQHGFPKAKSEGL-EEALMGTEVYDS----DMCSPVDVQMLDEITEEQICVEA--
          spotted gar   ELEHNNSLVNEEKET----SDARKKALIATALSKSGERLQALTLLMIHYRAGIEDLESIESTSPSEQ----QVKTKGT-EDTMVNAEVFEN----EMCSPVEAQMLDDITEEQICAEA--
         little skate   ELEQNYALQKEEKDC----TDSKKKCHLSAAISKSIERLQALTLLTIHYKAGIDDLEFSDFASSEQQ----AKKASKL-TDDILSLTTDDI----DVGSQADVQPLTEESNEQTIDET--
```

```
                               1450      1460      1470      1480      1490      1500
                         =========+=========+=========+=========+=========+=========

     polychaete worm     ------------------------------------------------------EGIV-
         human louse     -----------------------------------------------------------
          sea urchin     -----------------------------------------------------------
         sea lamprey     DDLAVIQGAVAEVSGATWEEGDAEEACAAAPEVCEDPSETGFALDVEEDDEEVGAKLLA
               human     ------------------------------------------------------ESV--
             opossum     ------------------------------------------------------EPL--
             chicken     ------------------------------------------------------QSVD-
tropical clawed frog     ------------------------------------------------------LEPEQ
          coelacanth     ------------------------------------------------------FS---
         Nile tilapia    ------------------------------------------------------LH---
           zebrafish     ------------------------------------------------------LP---
         spotted gar     ------------------------------------------------------MP---
        little skate     ------------------------------------------------------ESIS-
```
