# Supplementary material for: Reevaluating Emx gene phylogeny: homopolymeric amino acid tracts as a potential factor obscuring orthology signals in cyclostome genes
Source: BMC Evol Biol. 2015 May 4;15:78. doi: 10.1186/s12862-015-0351-z (PMC4464114; doi:10.1186/s12862-015-0351-z)
Supplement: Additional file 11: Table S5. — Sequence dataset for counts of homopolymeric amino acid tracts. This table summarizes the details of the data sets prepared for sea lamprey, zebrafish and human. [file 12862_2015_351_MOESM11_ESM.pdf]

Additional file 11 (Table S5). Sequence dataset for counts of homopolymeric amino acid tracts.

Species	Source	Number of sequences					# of HPAA tracts
		Peptides	Genes	Homolog set*	Non-redundant sequences	HPAA tract-containing peptides	
Consortium dataset							
Sea lamprey	Genome Consortium	24271	24271	6204	6204	898 (14.5%)	1359
Human	NCBI Refseq	36312	19969	14867	8123	989 (12.2%)	1462
Zebrafish	NCBI Refseq	27216	26504	10908	10673	940 (8.8%)	1354
NCBI mRNA dataset							
Sea lamprey	NCBI mRNA	1088	N/A	170	158	43 (27.2%)	84
Human	NCBI Refseq	36312	19969	875	408	65 (15.9%)	92
Zebrafish	NCBI Refseq	27216	26504	570	560	53 (9.5%)	69
Ensemble dataset							
Sea lamprey	Ensemble	11442	10415	7153	6565	697 (10.6%)	915
Human	NCBI Refseq	36312	19969	16764	9138	1121 (12.3%)	1668
Zebrafish	NCBI Refseq	27216	26504	11923	11675	1072 (9.2%)	1529

*See Methods for the detail of the preparation of these data sets.

Abbreviation: HPAA, homopolymeric amino acid.
