# Supplementary material for: Reevaluating Emx gene phylogeny: homopolymeric amino acid tracts as a potential factor obscuring orthology signals in cyclostome genes
Source: BMC Evol Biol. 2015 May 4;15:78. doi: 10.1186/s12862-015-0351-z (PMC4464114; doi:10.1186/s12862-015-0351-z)
Supplement: Additional file 13: Table S6. — Gene Ontology terms overrepresented among sea lamprey peptides with homopolymeric amino acid tracts. Gene Ontology terms revealed to be overrepresented both in Genome Consortium dataset and Ensembl dataset are listed. [file 12862_2015_351_MOESM13_ESM.pdf]

Additional file 13 (Table S6). Gene Ontology terms overrepresented among sea lamprey peptides with homopolymeric amino acid tracts.

Homopolymeric amino acid	Overrepresented term	Gene symbols for human homologs
P (Proline)	structural constituent of cell wall	*FMNL2, REPS1, SF1, RAPH1, ASAP2, ZFHX3*
G (Glycine)	RNA polymerase II transcription factor activity	*SOX9, ONECUT2, ISL1, PURA, JUN,TAF6L*
	enzyme activator activity*	*AGAP3, SIPA1L1, JUN, TAOK1, MMP24*
Q (Glutamine)	RNA polymerase II transcription factor activity	*PURA, MITF, CNOT2, JUN, ZFHX3, SF1, FOXA2, RFX3*
	transcription activator activity	*CREBBP, MITF, GLI2, FOXA2, FUBP3, NCOA2, ARID1B*
	transcription repressor activity	*ZFHX3, SF1, NCOA2, SIN3A, RFX3*
	structural constituent of cell wall	*CREBBP, ZFHX3, SF1*
	neurotransmitter binding*	*HTR1F, SSTR1, CHRNA4, GABRA4, DRD2*
	amine binding*	*GOLGA4, DRD2, CHRNA4, HTR1F, PAK4*

The listed terms were overrepresented in both Genome Consortium dataset and Ensembl datasets (see Methods).

The table contains only GO terms assigned for Molecular Function ($p < 0.05$).

*Overrepresentation of these terms is unique to sea lamprey.
