# Supplementary material for: Reevaluating Emx gene phylogeny: homopolymeric amino acid tracts as a potential factor obscuring orthology signals in cyclostome genes
Source: BMC Evol Biol. 2015 May 4;15:78. doi: 10.1186/s12862-015-0351-z (PMC4464114; doi:10.1186/s12862-015-0351-z)
Supplement: Additional file 14: — Data S5. Multiple sequence alignment of deduced amino acid sequences of Emx genes. Multiple sequence alignments produced by the three alignment programs ClustalW (a), MAFFT (b), or T-Coffee (c) are shown for a region containing the A-tract and the Q-tract in the lamprey Emx sequences that are indicated with gray background. [file 12862_2015_351_MOESM14_ESM.pdf]

Additional file 14 (Supplementary Data 5)

(a) Multiple alignment with ClustalW

```
                                   70        80        90       100       110       120       130       140       150       160       170       180       190       200       210       220       230       240       250       260       270       280       290       300       310       320       330
                          =========+=========+=========+=========+=========+=========+=========+=========+=========+=========+=========+=========+=========+=========+=========+=========+=========+=========+=========+=========+=========+=========+=========+=========+=========+=========+=========+
          human louse Emx IVGGTGSNRIQYSINNKTDRNHDKTIVDNCKIQTSKSSTFPNDEPEDEEDDEEDEIINPGSPSSDVSNKFESRSPADLRYNGNRTPIDASPTNLIIKNNSAFRSGSPFPTEGKISSYRYPKTASPHRSTDQDISKFESSIHSNSPRS------SISPASGERTSLSPKASLIKPSVLTPANVVPFGSEMNAAAALKGIYLPTDPVMHGVGHPSHHHHPHPLALAAAAQHFQAAGLAAAIAQHQQQPGGFLSSGPPSHLPPGHPGHHGPQI
          sea urchin Emx  LVSKDTN-------------------------HTNNHHRHHRATPTP---------------------------PTSPQAAATASTMESNSL-------------------------------------------ASSPFTPPATSRHGGIIKPMPTEASFFNTLSGMKGLYHP----------------------------EPIYHTAAAAGPPSHVGPIQHQLGLAHHHNMAAAAAAAAAQHNHPALAGVFGAHGLGG----------
          amphioxus Emxb  LVSKDH----------------------------LARDGPPRTLPHP---------------------------SQLLELSRLCRTTATTT--------------------------------------------INSGIPHVPYTQG-----------GIPVSLPSLGSLCGGPR-------------------------PVSVPHYVTYSAPTMVPPGLLSGAPP------------------TVPHQSPVSPPY-------------
      sea lamprey EmxA LVAKDGGDS-VSAAATLQQQ------------QQQQQQQQQQQQQQQ---------------------------QPAYPIPGGANPLTC----------------------------------------------GAQPPHPFAAA-------AAAAAAAAASRAGMHPELFFH----------------------------DAHHLQPLLAVPAMHPSGH-HH---------------HHHLSHPPPSLPLFGGPQG------------
      sea lamprey EmxB LVAKD-----CPAPTSRSEQ------------QQQQQQQQQHEAPLR---------------------------PAALSFAGTHNHQHH----------------------------------------------QHQQHHHHSSASVS---AAAAAAAAAAAAAAFVPAFPQP----------------------------TARALYPHPAELLYSDPGH-HPGSAGGPL-QVPALPPHHHLQH---SHPLFGPPQ-------------
           human Emx1     LVAKDGGTGGGTGGGGAGSH------------LLAAAAS---EEPLR---------------------------PTALNYPHPSAAEAA----------------------------------------------FVSGFPAAAAAG-------------AGRSLYGGPELVFP----------------------------EAMNH------PALTVHPA-HQLGA-------------SPLQ---PPHSFFGAQH-------------
         chicken Emx1     LVAKD-----------------------------TPLSS---EEPLR---------------------------PSALNYPPP-AASDA----------------------------------------------FPGGFQGAAAG----------------RALYGGTELVFP----------------------------EAVGH------PALPVAP--HQLGA-------------SPLP---HPHSFFGPQH-------------
tropical clawed frog Emx1 LVAKD-----------------------------NPLSS---EEPLR---------------------------PAAL--PYPGAPAEA----------------------------------------------FVSGFP-SPAG----------------RSLYNNPELVFP----------------------------ETVSH------PPLTVHP--HQLGA-------------SHLQ---HPHSFFAPQH-------------
      coelacanth Emx1     LVAKD-----------------------------SPLTA---EEPIR---------------------------PTAFSYPNP---AEA----------------------------------------------FVNGFQ-TTAG----------------RSLYTSPELMFP----------------------------EAVSH------PSLTVHP--HQLGT-------------PHLQ---HPHSFFGPQH-------------
    Nile tilapia Emx1     LVAKE-----------------------------NPLTA---EDPIR---------------------------PTALSYSNPT--TDA----------------------------------------------LMNSYQAPPPA----------------RSLYQSPDLVFP----------------------------ETMNH------PSLTVAP--HQLGG-------------SHLQ---HPH-FFGTQH-------------
     spotted gar Emx1     LVAKE-----------------------------SPLTA---EEPIR---------------------------PTALSYSNP---TDA----------------------------------------------FMTGFQG-QAG----------------RSLYSGPELVFP----------------------------ETVNH------PSLTVHP--HQLGS-------------SHLQ---HPHSFFGTQH-------------
small-spotted catshark Emx1 LVAKDH----------------------------NPLSP---EEPLR---------------------------PAAVTYPPS---TDA----------------------------------------------FAGGLQ--AAA----------------RSLYPGPELMFQ----------------------------EAVSH------PSLPVH-P-HQLGT-------------HHLR---SHHTFFAPQH-------------
     little skate Emx1    LVAKEN----------------------------SPLSP---DESLR---------------------------PAALSYPTS---ADG----------------------------------------------FPNAFQ--APG----------------RSLYSGPDLMFQ----------------------------EAVAH------PALPVHHP-HPLSS-------------HHLQ---TPHPFFSPQH-------------
           human Emx2     LVAKD-----------------------------SPLPASRSEDPIR---------------------------PAALSYANSS-PINP----------------------------------------------FLNGFHSAAAAA-------------AGRGVYSNPDLVFA----------------------------EAVSHPPNPAVPVHPVPPP-HALAA-------------HPLPSSHSPHPLFASQQ-------------
         chicken Emx2     LVAKD-----------------------------SPLPASRSEDPIR---------------------------PAALSYANSS-PMNP----------------------------------------------FLNGFHS------------------TGRGVYSNPDLVFA----------------------------EAVSHPPNPAVPVHPVPPP-HALAA-------------HPLPASHSTHPLFASQQ-------------
tropical clawed frog Emx2 LVAKD-----------------------------SPLPVSRSEEPIR---------------------------PAALSYANSA-PMNP----------------------------------------------FLNGFHP------------------TGRGVYSNPDLVFA----------------------------EAVSHPPNPAVPVHPVPPP-HALAA-------------HPLPTSHSPHPLFASQQ-------------
      coelacanth Emx2     LVAKD-----------------------------SPLPVSRSEDPIR---------------------------PSALSYANSA-PMNP----------------------------------------------FLNGFHS------------------SSRSVYNNPDLLFA----------------------------EAVSHPPNAAVPVHPVPPP-HALAA-------------HPLPSSHTPHPLFASQQ-------------
    Nile tilapia Emx2     LVAKD-----------------------------NPVPASRTEEPIR---------------------------PAALSYANSG-QMNP----------------------------------------------FLNGFHS------------------GGRGVYSNPDLVFA----------------------------EAVSHPPNSTVPVHPVAPP-HALAA-------------HPLSSSHSPHPLFASQQ-------------
     spotted gar Emx2     LVAKD-----------------------------NPLPASRSEEPIR---------------------------PAALSYANST-QMNP----------------------------------------------FLNGFHS------------------SGRGVYSNPDLVFA----------------------------EAVSHPPNSAVPVHPVPPP-HALAA-------------HPLSSSHSPHPLFASQQ-------------
small-spotted catshark Emx2 LVAKD-----------------------------SPLPASRSEEPIR---------------------------PAALSYANPS-SVNP----------------------------------------------FLNGFHT------------------GGRALYSNPDLVFA----------------------------DAVAHQTSPAVPVHPVPP--HALAG-------------HPLQSSHSPHPLFTSQQ-------------
     little skate Emx2    LVAKD-----------------------------SPLPASRSEEPIR---------------------------PAALSYANSS-PVNP----------------------------------------------FLNGFHT------------------SGRAIY-NPELVFA----------------------------DAVSHQTNTAVPVHPVPP--HALAA-------------HPLQSSHSPHPLFASQQ-------------
tropical clawed frog Emx3 LVGPDR----------------------------------TPEEPLR---------------------------PTALKYPEPGHGLTP------------------------------------------------TPLRP-------------------GVRLLGAPDLFFP----------------------------EPAAPPYG------------PSLG-----------------------LPQHRILH-------------
    Nile tilapia Emx3     LVGKDG----------------------------SSHGGGSGEEPIR---------------------------PTALRFPDSLHPAAAAGV-------------------------------------------FGSCFQGS-----------------NGRTLF--PDMVLQ----------------------------EPGTHAQGPGGLPLHP----LQIPP----------------------QSFFSPQH-------------
     spotted gar Emx3     LVGKDA----------------------------NSS---AGDEPVR---------------------------PTALRFTESVHPTP-----------------------------------------------FGSCFQN------------------TGRTLYSTPDLMFS----------------------------EPGTHAASS-ALSLHP----LQLPA----------------------QPFFGPHQ-------------
small-spotted catshark Emx3 LVGKDS----------------------------PST---LGDEPLR---------------------------PTALRYADSVHASP-----------------------------------------------FGSGFQN------------------AGRTLYGSPELLFP----------------------------EPVAHAASP-ALSMHPGNP-HHLPA----------------------QSFFAPQQ-------------
     little skate Emx3    LVGKDN----------------------------AST---LGDEPLR---------------------------PTALRYADSLHASH-----------------------------------------------FGSGFQN------------------AGRTLYSSPELLFP----------------------------DPVTHAASP-ALSIHPGNA-HHLSA----------------------QSFFAPQQ-------------
```

(b) Multiple alignment with MAFFT

```
                                   70        80        90       100       110       120       130       140       150       160       170       180       190       200       210       220       230       240       250       260       270       280       290       300       310       320       330
                          =========+=========+=========+=========+=========+=========+=========+=========+=========+=========+=========+=========+=========+=========+=========+=========+=========+=========+=========+=========+=========+=========+=========+=========+=========+=========+=========+
          human louse Emx IVGGTGSNRIQYSINNKTDRNHDKTIVDNCKIQTSKSSTFPNDEPEDEEDDEEDEIINPGSPSSDVSNKFESRSPADLRYNGNRTPIDASPTNLIIKNNSAFRSGSPFPTEGKISSYRYPKTASPHRSTDQDISKFESSIHSNSPRSSISPASGERTSLSPK-----ASLIKPSVLTPANV--------------------------------VPFGSEMNAAA----------------A-LKGIYLPT------DPVMHGV--GHP-S
          sea urchin Emx  LVSKDTNHTN----------NH----------------------------------------------------------------------------------------------------------------------HRHHR-------ATP------------TPPTSPQAAATAST-----------------------------MESNSLASSPFTPPATSRHGGIIKPMPTEASFFNTLSGMK-GLYHPEPIYHTAAAAGP-P
          amphioxus Emxb  LVSKD------------------------------------------------------------------------------------------------------------------------------------------HLARDGPPRTLPHPSQLLEL----SRLCRTTATTTINS---------------------------------GIPHVPYT------QGGIPVSLP---S-LGSLCGGPRPVSVPHYVTYSAPTMVP--
      sea lamprey EmxA LVAKD------------------------------------------------------------------------------------------------------------------------------------------GGDSVSAAATLQQQQQQQQQQQQQQQQQQQPAYPIPGGANPLTCG---------------------AQPPHPFAAAAAAAAAA--------------A-ASRAGMHP-ELFFHDAHHL-----QP-L
      sea lamprey EmxB LVAKD------------------------------------------------------------------------------------------------------------------------------------------CPA----PTSRSEQQQQQQQQQQHEAPLRPAALSFAGTHNHQHHQHQQHHHHSSASVSAAAAAAAAAAAAAAFVPAFPQ-P----------------T-ARALYPHPAELLYSDPGHHPG---SA-G
           human Emx1     LVAKDGGTGG---------------------------------------------------------------------------------------GTGGGGAGS----------------------------------HLLAAA-------AS------------EEPLRPTALNYPHP----------------------------SAAEAAFVSGFPAAAAA-------------GA-GRSLYGGP-ELVFPEAMNHP-------A
         chicken Emx1     LVAKD------------------------------------------------------------------------------------------------------------------------------------------TPL-------SS------------EEPLRPSALNYPPP-----------------------------AASDAFPGGFQGAA----------------A-GRALYGGT-ELVFPEAVGHP-------A
tropical clawed frog Emx1 LVAKD------------------------------------------------------------------------------------------------------------------------------------------NPL-------SS------------EEPLRPAALPYPGA------------------------------PAEAFVSGFPS-P----------------A-GRSLYNNP-ELVFPETVSHP-------P
      coelacanth Emx1     LVAKD------------------------------------------------------------------------------------------------------------------------------------------SPL-------TA------------EEPIRPTAFSYPN-------------------------------PAEAFVNGFQT-T----------------A-GRSLYTSP-ELMFPEAVSHP-------S
    Nile tilapia Emx1     LVAKE------------------------------------------------------------------------------------------------------------------------------------------NPL-------TA------------EDPIRPTALSYSNP------------------------------TTDALMNSYQAPP----------------P-ARSLYQSP-DLVFPETMNHP-------S
     spotted gar Emx1     LVAKE------------------------------------------------------------------------------------------------------------------------------------------SPL-------TA------------EEPIRPTALSYSN-------------------------------PTDAFMTGFQG-Q----------------A-GRSLYSGP-ELVFPETVNHP-------S
small-spotted catshark Emx1 LVAKDH-----------------------------------------------------------------------------------------------------------------------------------------NPL-------SP------------EEPLRPAAVTYPP-------------------------------STDAFAGGLQA------------------A-ARSLYPGP-ELMFQEAVSHP-------S
     little skate Emx1    LVAKEN-----------------------------------------------------------------------------------------------------------------------------------------SPL-------SP------------DESLRPAALSYPT-------------------------------SADGFPNAFQA------------------P-GRSLYSGP-DLMFQEAVAHP-------A
           human Emx2     LVAKD------------------------------------------------------------------------------------------------------------------------------------------SPL----PASRS------------EDPIRPAALSYANS-----------------------------SPINPFLNGFHSAAAA-------------AA-GRGVYSNP-DLVFAEAVSHPP---NP-A
         chicken Emx2     LVAKD------------------------------------------------------------------------------------------------------------------------------------------SPL----PASRS------------EDPIRPAALSYANS-----------------------------SPMNPFLNGFHS------------------T-GRGVYSNP-DLVFAEAVSHPP---NP-A
tropical clawed frog Emx2 LVAKD------------------------------------------------------------------------------------------------------------------------------------------SPL----PVSRS------------EEPIRPAALSYANS-----------------------------APMNPFLNGFHP------------------T-GRGVYSNP-DLVFAEAVSHPP---NP-A
      coelacanth Emx2     LVAKD------------------------------------------------------------------------------------------------------------------------------------------SPL----PVSRS------------EDPIRPSALSYANS-----------------------------APMNPFLNGFHS------------------S-SRSVYNNP-DLLFAEAVSHPP---NA-A
    Nile tilapia Emx2     LVAKD------------------------------------------------------------------------------------------------------------------------------------------NPV----PASRT------------EEPIRPAALSYANS-----------------------------GQMNPFLNGFHS------------------G-GRGVYSNP-DLVFAEAVSHPP---NS-T
     spotted gar Emx2     LVAKD------------------------------------------------------------------------------------------------------------------------------------------NPL----PASRS------------EEPIRPAALSYANS-----------------------------TQMNPFLNGFHS------------------S-GRGVYSNP-DLVFAEAVSHPP---NS-A
small-spotted catshark Emx2 LVAKD------------------------------------------------------------------------------------------------------------------------------------------SPL----PASRS------------EEPIRPAALSYANP-----------------------------SSVNPFLNGFHT------------------G-GRALYSNP-DLVFADAVAHQT---SP-A
     little skate Emx2    LVAKD------------------------------------------------------------------------------------------------------------------------------------------SPL----PASRS------------EEPIRPAALSYANS-----------------------------SPVNPFLNGFHT------------------S-GRAIY-NP-ELVFADAVSHQT---NT-A
tropical clawed frog Emx3 LVGPD---------------------------------------------------------------------------------------------------------------------------------------------------RTP------------EEPLRPTALKYPEP-------------------------------GHGLTPTPLR------------------P-GVRLLGAP-DLFFPEPAAPPY---GP-S
    Nile tilapia Emx3     LVGKD------------------------------------------------------------------------------------------------------------------------------------------GSSH---GGGSG------------EEPIRPTALRFPDSLHP-------------------------AAAAGVFGSCFQG------------------SNGRTLF--P-DMVLQEPGTHAQ---GPGG
     spotted gar Emx3     LVGKD------------------------------------------------------------------------------------------------------------------------------------------ANS------SAG------------DEPVRPTALRFTES-----------------------------VHPTPFGSCFQN------------------T-GRTLYSTP-DLMFSEPGTHAA---SS-A
small-spotted catshark Emx3 LVGKD------------------------------------------------------------------------------------------------------------------------------------------SPS------TLG------------DEPLRPTALRYADS-----------------------------VHASPFGSGFQN------------------A-GRTLYGSP-ELLFPEPVAHAA---SP-A
     little skate Emx3    LVGKD------------------------------------------------------------------------------------------------------------------------------------------NAS------TLG------------DEPLRPTALRYADS-----------------------------LHASHFGSGFQN------------------A-GRTLYSSP-ELLFPDPVTHAA---SP-A
```

(c) Multiple alignment with T-Coffee

```
                                  110       120       130       140       150       160       170       180       190       200       210       220       230       240       250       260       270       280       290       300       310       320       330       340       350       360       370
                          =========+=========+=========+=========+=========+=========+=========+=========+=========+=========+=========+=========+=========+=========+=========+=========+=========+=========+=========+=========+=========+=========+=========+=========+=========+=========+=========+
          human louse Emx NKTDRNHDKTIVDNCKIQTSKSSTFP-------------------------------------NDEPEDEEDDEEDEIINPGSPSSDVSNKFE----------------SRSPADLRYNGNRTPIDASPTNLIIKNNSAF-------------------------RSGSPFPTEGKISSYRYPKTASPHRSTDQDISKFESSIHSNSPRSSISPASGERTSLSPKASLIKPSVLT-PANV---VPFGS-EMNAA---------AAL----
          sea urchin Emx  ------------------------HT-------------------------------------NNHH-----------RHHRA------TPTP----------------PTSPQAAATAST---------------------------------------------------------------------------------MES----------------NSLASSPFTPPATSRHGGIIKPMPTEASFFNTL---------SGM----
          amphioxus Emxb  -------------------------R-----------------------------------------------------------------DGPPRTLPHPSQLLELSRLCRTTATTTINSGI--------------P-----------------------------------------------------------------H---------------------------------------VPYTQ-GGIPVSLPSLGSLCGGPRPVS
      sea lamprey EmxA -------------------------AAATLQQQQQQQQQQQQQQQQQQQPAYPIPGGANPLTCG---------------------------AQ----------------PPHPFAAAAAAAA---------------------------------------------------------------------------------------------------------------------------------AA----------AAS----
      sea lamprey EmxB ---------------EQQQQQQQQQQ-------------------------------------H---------------------------EA----------------PLRPAALSFAGTH---------------NHQHHQHQQHHHHSSASVSAAAAAAAAAA-------------------------------------A-----------------------------------A---AAFVP-AFPQ----------PTA----
           human Emx1     -------------------------A-------------------------------------S---------------------------EE----------------PLRPTALNYPHPS---------------A-----------------------------------------------------------------A-----------------------------------E---AAFVS-GFPAAAA-A-----GAG----
         chicken Emx1     -------------------------S-------------------------------------S---------------------------EE----------------PLRPSALNYPPPA---------------A-----------------------------------------------------------------------------------------------------S---DAFPG-GFQGA---------AAG----
tropical clawed frog Emx1 -------------------------S-------------------------------------S---------------------------EE----------------PLRPAALPYPG-A---------------P-----------------------------------------------------------------------------------------------------A---EAFVS-GFPS----------PAG----
      coelacanth Emx1     -------------------------T-------------------------------------A---------------------------EE----------------PIRPTAFSYPNP----------------------------------------------------------------------------------------------------------------------A---EAFVN-GFQT----------TAG----
    Nile tilapia Emx1     -------------------------T-------------------------------------A---------------------------ED----------------PIRPTALSYSNPT---------------------------------------------------------------------------------------------------------------------T---DALMN-SYQAP---------PPA----
     spotted gar Emx1     -------------------------T-------------------------------------A---------------------------EE----------------PIRPTALSYSNPT-------------------------------------------------------------------------------------------------------------------------DAFMT-GFQG----------QAG----
small-spotted catshark Emx1 -------------------------S-------------------------------------P---------------------------EE----------------PLRPAAVTYPPS----------------------------------------------------------------------------------------------------------------------T---DAFAG-GLQA-----------AA----
     little skate Emx1    -------------------------S-------------------------------------P---------------------------DE----------------SLRPAALSYPTS----------------------------------------------------------------------------------------------------------------------A---DGFPN-AFQA-----------PG----
           human Emx2     -------------------------R-------------------------------------S---------------------------ED----------------PIRPAALSYANSS---------------P-----------------------------------------------------------------------------------------------------I---NPFLN-GFHSAAA-A-----AAG----
         chicken Emx2     -------------------------R-------------------------------------S---------------------------ED----------------PIRPAALSYANSS---------------P-----------------------------------------------------------------------------------------------------M---NPFLN-GFHS-----------TG----
tropical clawed frog Emx2 -------------------------R-------------------------------------S---------------------------EE----------------PIRPAALSYANSA---------------P-----------------------------------------------------------------------------------------------------M---NPFLN-GFHP-----------TG----
      coelacanth Emx2     -------------------------R-------------------------------------S---------------------------ED----------------PIRPSALSYANSA---------------P-----------------------------------------------------------------------------------------------------M---NPFLN-GFHS-----------SS----
    Nile tilapia Emx2     -------------------------R-------------------------------------T---------------------------EE----------------PIRPAALSYANSG---------------Q-----------------------------------------------------------------------------------------------------M---NPFLN-GFHS-----------GG----
     spotted gar Emx2     -------------------------R-------------------------------------S---------------------------EE----------------PIRPAALSYANST---------------Q-----------------------------------------------------------------------------------------------------M---NPFLN-GFHS-----------SG----
small-spotted catshark Emx2 -------------------------R-------------------------------------S---------------------------EE----------------PIRPAALSYANPS---------------S-----------------------------------------------------------------------------------------------------V---NPFLN-GFHT-----------GG----
     little skate Emx2    -------------------------R-------------------------------------S---------------------------EE----------------PIRPAALSYANSS---------------P-----------------------------------------------------------------------------------------------------V---NPFLN-GFHT-----------SG----
tropical clawed frog Emx3 -------------------------------------------------------------------------------------------EE----------------PLRPTALKYPEPG-------------------------------------------------------------------------------------------------------------------------HGLTP-TPLR-----------PG----
    Nile tilapia Emx3     -------------------------S-------------------------------------G---------------------------EE----------------PIRPTALRFPDSL---------------HPA-------------------------A-------------------------------------A-----------------------------------A---GVFGS-CFQG----------SNG----
     spotted gar Emx3     -------------------------A-------------------------------------G---------------------------DE----------------PVRPTALRFTESV---------------H-----------------------------------------------------------------------------------------------------P---TPFGS-CFQN-----------TG----
small-spotted catshark Emx3 -------------------------L-------------------------------------G---------------------------DE----------------PLRPTALRYADSV---------------H-----------------------------------------------------------------------------------------------------A---SPFGS-GFQN-----------AG----
     little skate Emx3    -------------------------L-------------------------------------G---------------------------DE----------------PLRPTALRYADSL---------------H-----------------------------------------------------------------------------------------------------A---SHFGS-GFQN-----------AG----
```
